# Compositionally Biased Regions Within Structured Protein Domains

**DOI:** 10.3390/biom16091222

**Published:** 2026-08-22

**Authors:** Paul M. Harrison

**Affiliations:** Department of Biology, McGill University, Montreal, QC H3A 1B1, Canada; paul.harrison@mcgill.ca

**Keywords:** compositional bias, low complexity, protein structure, intrinsic disorder, nucleotide binding, ligand binding, protein binding

## Abstract

In proteins, tracts compositionally biased by a subset of amino acids are often linked to intrinsic disorder. Such compositionally biased regions (CBRs) are sometimes analyzed for ‘sequence complexity’ (‘information entropy’) despite being an unlikely substrate for natural selection per se, and therefore not functionally implicated. Here, an algorithmic strategy applying compositional bias detection was designed to capture the wide diversity of CBRs in *structured* protein domains (termed ‘sCBRs’), ranging from trihomopeptides to >200 residues, with conservation and partner binding as functional lenses. sCBRs are common, with about 1/4th of domains harbouring them, but domains dominated by sCBRs over >50% of their lengths are rare (~1 in 200). Despite general assumptions, very short sCBRs are highly significantly sequence-conserved, and associated with ligand binding, even when common nucleotide/phosphate-binding or glycine-rich cases are disregarded. However, regardless of length, ~50% of cases are evolutionarily dynamic, undergoing clade-specific expansion/contraction. Protein-binding associations include aversions for short (≤16 residues) valine-rich regions in protein interfaces, and enrichments of longer alanine-rich cases (>16 residues). Only ~5% of cases are (at least partly) in intrinsically disordered loops, and are significantly shorter than sCBRs generally. Functional implications of sCBRs are discussed with many examples. The sCBR data might help with hypothesis generation and protein design/engineering.

## 1. Introduction

Proteins often have tracts that are simpler, more repetitive or biased towards a small subset of the amino-acid residues. These sequences are sometimes thus informationally less complex. Sequence complexity and the lack of it have been analyzed algorithmically since the work of Wootton and Milosavljevic in the early 1990s [1,2,3,4]. ‘Low-complexity’ refers to the parts of biologically sequences that can be algorithmically defined as having low information entropy, deviating far from a perfect sampling of all twenty amino acids, although the term was originally coined much earlier in the description of DNA fractions [5,6]. In the scientific literature, terms such as ‘low-complexity domain’ or ‘low-complexity region’ (LCR) are now applied with vernacular abandon. However, compositional bias is a more general notion, referring simply to an enrichment of a subset of residue types, and can range from mild skews in amino-acid content to pure homopeptide [7]. LCRs are often treated ontically as a basic feature category to annotate (along with features like ‘protein domains’, ‘intrinsically disordered regions’, ‘transmembrane regions’, and ‘signal peptides’), but they are neither predictable entities, nor conceptually definite (e.g., like *homology*—two proteins are either homologous or they are not). That is, one investigator’s lowness of complexity might not be another’s, and it is not possible to draw an imaginary boundary around the concept [7]. LCR definition is heavily dependent on parameter choice for any specific algorithm—different parameters cover wildly variant amounts of sequence and annotate widely diverse region lengths [7]; thus it is also not possible to derive LCR consensuses from different algorithms, in the same manner as for predictable features.

However, a partial solution to such problems can be achieved through analysis of the broad spectrum of compositional biases, and probing it through functional lenses [8]. Indeed, sequence complexity per se is unlikely to be under selection for function, whereas a bias for specific residues might have specific roles (e.g., the glutamine/asparagine enrichments in prion and prion-like domains [9]).

Lower sequence complexity and obvious compositional biases have been more associated with intrinsically disordered regions (IDRs) in proteins, than structured domains [10,11]. IDRs are protein tracts that remain unfolded during at least a part of their normal functioning. They are most often discovered from NMR spectroscopy data, or inferred from missing electron density in X-ray crystal structures. Low-complexity IDRs have been implicated in phase separation mechanisms [12,13,14], and have positional functional linkages [15]. Compositionally biased IDRs have specific functional roles, e.g., hundreds of hypothetical functional associations were discovered for such regions in *Saccharomyces cerevisiae* [8]. Such IDRs have a lot of diverse sequence characteristics whose significance has not yet been fully assessed or appreciated. They can have various types of patterning such as: repeats, alternating residue blocks or bands, compositional modules/motifs, and amino-acid runs or ‘homopeptides’ [16]. Such residue patterning in IDRs can have functional significance, e.g., Sup35p prion repeats are linked to chaperone-dependent prion maintenance, but not to prion nucleation or fibre growth [17]; charged residue blocks engender function in transcriptional regulators and nucleolar proteins [18,19]; homopeptides in transcriptional activators modulate expression levels [20,21]; and compositional modules in stress-response proteins such as the water stress sensor protein FLOE-1 in *Arabidopsis* have distinct roles [22].

The prevalence of LCRs has been surveyed in protein structures using the LCR annotator SEG in default mode [2,23,24] indicating that they tend to be exposed, and have a helical or coil secondary structure [23,24], although LCRs in structures solved through 2D-NMR spectroscopy look more like disordered LCRs [24]. Using information entropy windowing similar to default SEG, variable secondary-structure tendencies were observed for LCRs composed of different amino-acid types [25]. In another survey applying SEG with a set of parameters targeting regions that are a bit longer than default, and sequence alignment to long homopeptide sequences, most cases were observed to be non-structured [26]. Homopeptides per se are favoured in intrinsic disorder, but the extent of this is dependent on organism [27]; in *Plasmodia*, structural homopeptides are enriched in areas adjacent to intrinsic disorder [28].

Given the greater functional and evolutionary relevance of compositional bias, as opposed to sequence complexity, a strategy was designed to capture the wide diversity of compositionally biased regions in protein structures (termed sCBRs for ‘structural CBRs’), ranging from trihomopeptides to >200 residues in length, with conservation and partner binding applied as functional lenses. The chief goal of this survey is to assess the functional relevance of the gamut of sCBRs. sCBRs are common, occurring in one in four protein domains. Domains dominated by them (over a majority of their sequences) are rare, but often engender dramatic solutions to functional problems. Contrary to general assumptions about biased regions, shorter sCBRs are often implicated in highly sequence-conserved functional roles, even when the common nucleotide-binding-linked sCBRs are set aside. Insights are gained into compositional biases linked to protein and ligand binding, including novel findings for documented ligand-binding subsequences, such as a marked tendency of glycine-rich nucleotide-binding motifs to gain rather than lose glycines clade-specifically, and evidence for the key role of the glycines in such motifs in enabling backbone hydrogen bonding to the ligand.

## 2. Materials and Methods

### 2.1. Sequence and Structure Data

The complete set of >22,000 UniProt reference proteomes were obtained in May 2024 [29]. These were split into Kingdom Lists: metazoans, fungi, plants, other eukaryotes, bacteria, archaea and viruses. Also at the same time, the *astralscop40* data set (version 2.08) of protein domain structures and sequences was downloaded in May 2024 [30]. This is a benchmark set of protein domains extracted from the Protein DataBank (PDB) [31], such that no two domain sequences share >40% sequence identity, that is commonly used as a reference set of domains reduced for sequence redundancy. Both SEQRES record and ATOM record sequences were obtained for *astralscop40*, so that intrinsically disordered regions that manifest as sequence tracts with no electron density in X-ray crystallographic structures can be examined. The *astralscop40* set was submitted to a previously derived procedure to further reduce redundancy through application of all-against-all BLASTP, with an e-value threshold of ≤1 × 10^−4^, sorting on decreasing number of alignment hits, and progressively de-selecting as the list is searched, producing a reduced list termed *astralscop40R* [11,32]. Structural coordinates for the biological assemblies containing these protein domains were downloaded from the PDB (www.rcsb.org).

### 2.2. Compositional Biases

Compositionally biased regions (CBRs) in the domain structures were defined as regions with significant compositional bias according to the fLPS2 algorithm [33,34,35]. Briefly, this algorithm optimizes the boundaries of compositionally biased regions by minimizing binomial probabilities. Background amino-acid frequencies for binomial probability calculation are by default taken from the *astralscop40* data set. fLPS2 uses three parameters: minimum window size (***m***), maximum window size (***M***), and binomial *p*-value threshold (***t***). CBRs have a bias signature, which is a list of the biasing amino-acid types in decreasing order of prominence, e.g., *{AP}* denotes a region with a primary bias of alanine, and a secondary bias of proline. By default, CBRs are annotated by fLPS2 with a *p*-value threshold of 0.001.

**Figure 1:**
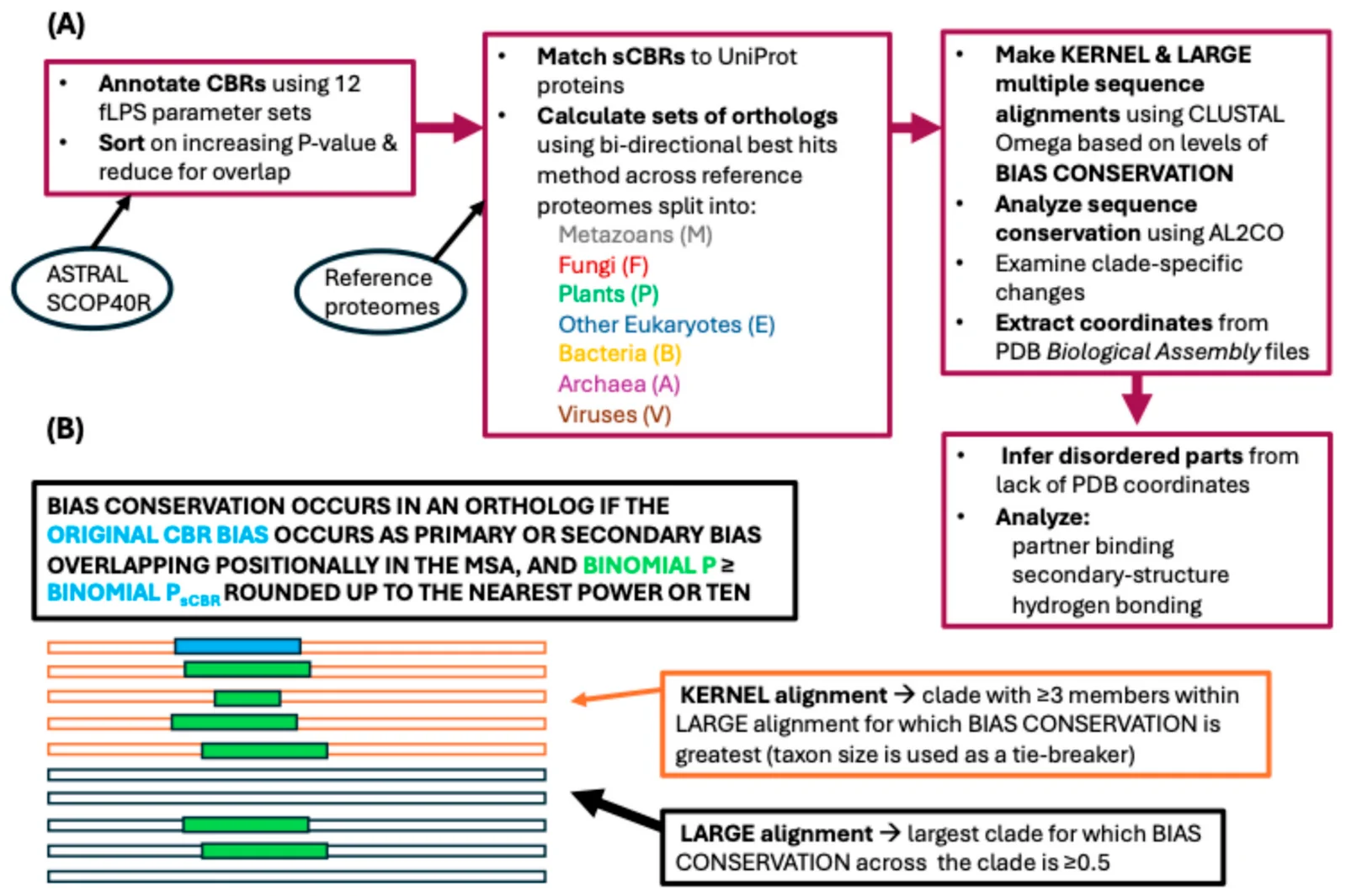
Method for finding structural compositionally biased regions (sCBRs). (**A**) The pipeline. (**B**) Graphical depiction of bias conservation assessment, and kernel and large alignments.

Since definition of boundaries depends on parameter choice [7], we apply a set of fLPS2 16 parameter sets (Table 1). This strategy was previously applied to labelling hundreds of hypothetical functions for CBRs that have intrinsic disorder propensity in budding yeast [8]. Here, the parameter sets have been derived from running the parameter chooser *fLPSparameters*, but they are adjusted to account for the shorter lengths and milder biases of CBRs within protein domains [7]. All annotations for each sequence were collated, sorted on increasing *p*-value, and reduced for overlap if they have the same primary bias (Figure 1A). Mainly C-rich CBRs were set aside since they are for a class of protein domain that are already well-studied, arising from high levels of disulphide bridges or metal-ion binding sites [36]. The actual frequencies of sCBRs are labelled according to the most restrictive parameters that find them (shortest target length and lowest sequence coverage) in Table 1. For example, about 1 in 14 domains has an sCBR labelled with the most restrictive parameters for target length = 6 and sequence coverage ≤ 5% in whole proteins. The parameter sets are labelled Len = x_Cov = y%, where region target length is *x* and data set coverage is *y*%, i.e., overall percentage of *whole* protein sequence residues from a benchmark set taken from UniProt, not structured domains per se. The frequencies in structured domains are much lower since the statistics for whole sequences comprise large tracts of intrinsic disorder, and they have also been filtered for bias conservation as described below [7,8].

### 2.3. Matching to UniProt Sequences and Ortholog Sets

Domain sequences with sCBRs were matched to source sequences from the UniProt reference proteins using BLASTP and a sequence identity threshold of ≥90% [32]. For each unique matched source protein, sets of orthologs across its Kingdom List were compiled using the bi-directional best hit method, as previously implemented for fungal proteomes [37].

### 2.4. Multiple Sequence Alignment, Bias Conservation and Sequence Conservation

sCBRs are defined as CBRs in structured protein domains. In particular, here the focus is on sCBRs that exhibit bias conservation (BC). Each set of orthologs was aligned using Clustal Omega (version 1.2) [38]. The resulting multiple sequence alignments (MSAs) were assessed for BC (Figure 1B). BC occurred in an ortholog if the original sCBR bias occurs as a primary or secondary bias in an orthologous CBR overlapping positionally in the MSA and with binomial P ≤ the P_sCBR_ rounded up to the nearest power of 10. For example, if P_sCBR_ = 7.7 × 10^−8^, then P should be ≤1 × 10^−7^. A *LARGE* alignment was extracted from the overall MSA such that it comprised sequences from the largest taxon across which BC is ≥0.5. A KERNEL alignment was extracted comprising the taxon with ≥3 members for which BC is greatest (with taxon size applied as a tie-breaker).

Sequence conservation was assessed using the AL2CO program with defaults [39].

### 2.5. Clade-Specific Size Changes

LARGE and KERNEL alignments were analyzed for clade-specific changes by trawling for clades ≥ 5 members containing the sCBR parent sequence that have a clade mean number of primary biasing residues significantly higher or lower than the whole alignment, using the distribution of the sample mean and a two-tailed *p*-value. A window of the size of the sCBR was searched on either side of it (i.e., 3× total sCBR length).

### 2.6. Secondary-Structure Prevalences

Secondary-structure assignments were extracted from the PDB files. To analyze these statistically, random samples of tracts of the same sizes as in the populations of sCBRs were generated, the overall proportions of each secondary-structure type calculated (F), and z-scores (Z) calculated for the distributions of the sample means (μ) for each F, e.g., Z_alpha-helix_ = (F_alpha-helix_ − μ_alpha-helix_)/σ_alpha-helix_. Two-tailed *p*-values were calculated.

### 2.7. Partner Binding

Binding to small-molecule ligands, and to protein or nucleic-acid partners was determined typically using a contact threshold of 5.0 Å to the nearest sCBR biasing residue. Thresholds of 4.0 Å and 6.0 Å were also checked, to assess threshold dependence. To assess the significance of binding and proximity, for each residue or group examined, 1000 simulated samples of polypeptide tracts from the relevant protein domains the same size as the sCBR were generated, and the actual values for distances and numbers of contacts below the threshold were compared to these samples to generate z-scores from the distribution of the sample mean. For comparison, for ligand binding only, randomly sampled distributions of the distance to the nearest ligand were calculated for the same groups and residues, yielding almost identical precedences of significance. For small-molecule ligands, possible false-positive ligands were filtered out using a list downloaded from the BioLiP database [40,41], and the remainder was cross-referenced for each PDB code with ligands listed in the PDBsum database [42].

### 2.8. Counting Hydrogen Bonds

Hydrogen bonds were counted with default HBPLUS (version 3.15) [43].

## 3. Results and Discussion

### 3.1. sCBRs Are Common in Protein Domains

**Figure 2:**
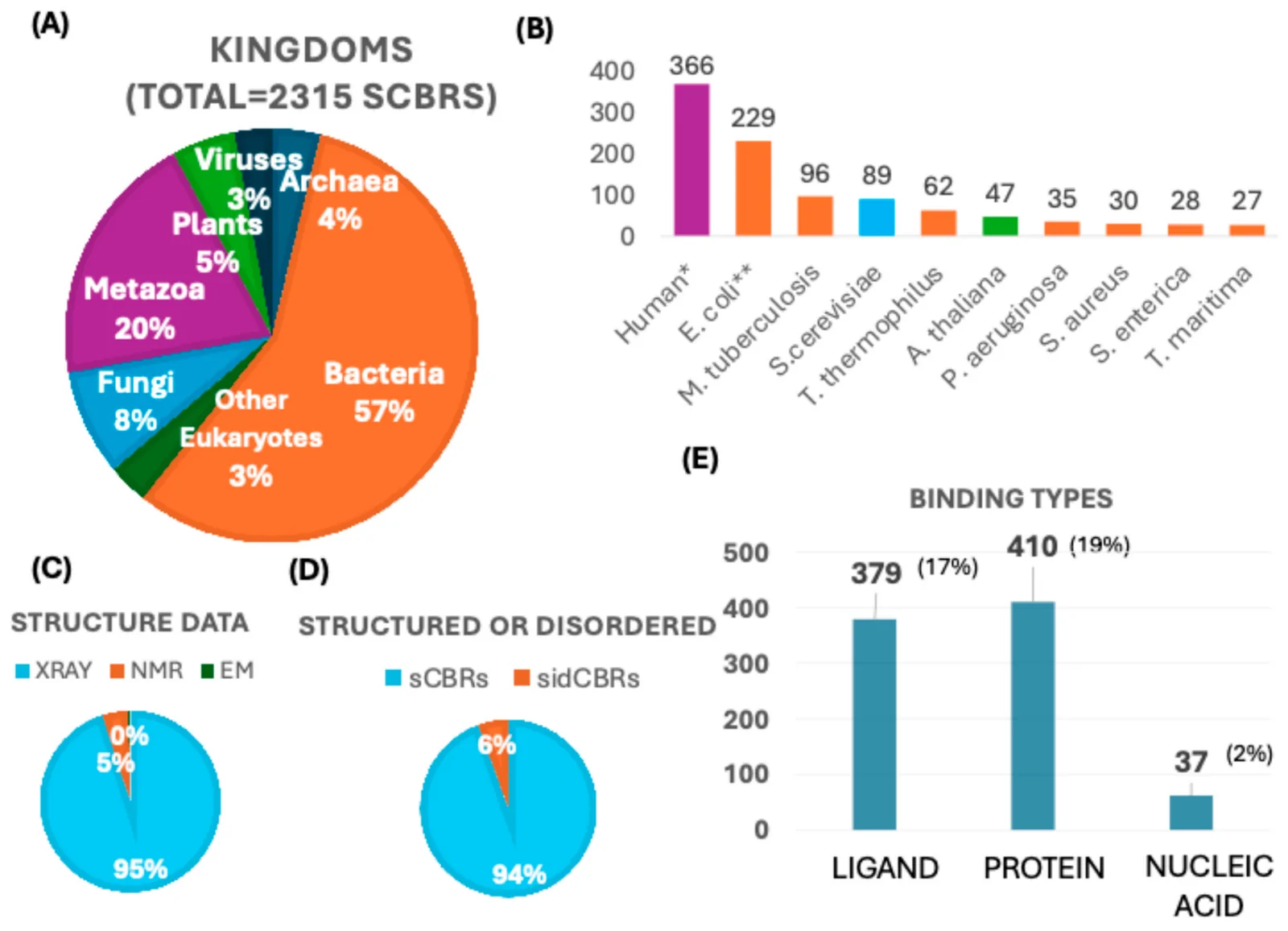
The data vista. (**A**) The sampling of kingdoms across the sCBR-containing domains. (**B**) The most prevalent species across the sCBR-containing domains. * contains cases assigned to identical great ape proteins. ** multiple strains. (**C**) Sources of the structural data (XRAY = x-ray crystallography, NMR = 2D-NMR spectroscopy, EM = electron microscopy). (**D**) sidCBRs = sCBRs containing intrinsic disorder. (**E**) sCBR binding types.

**Figure 3:**
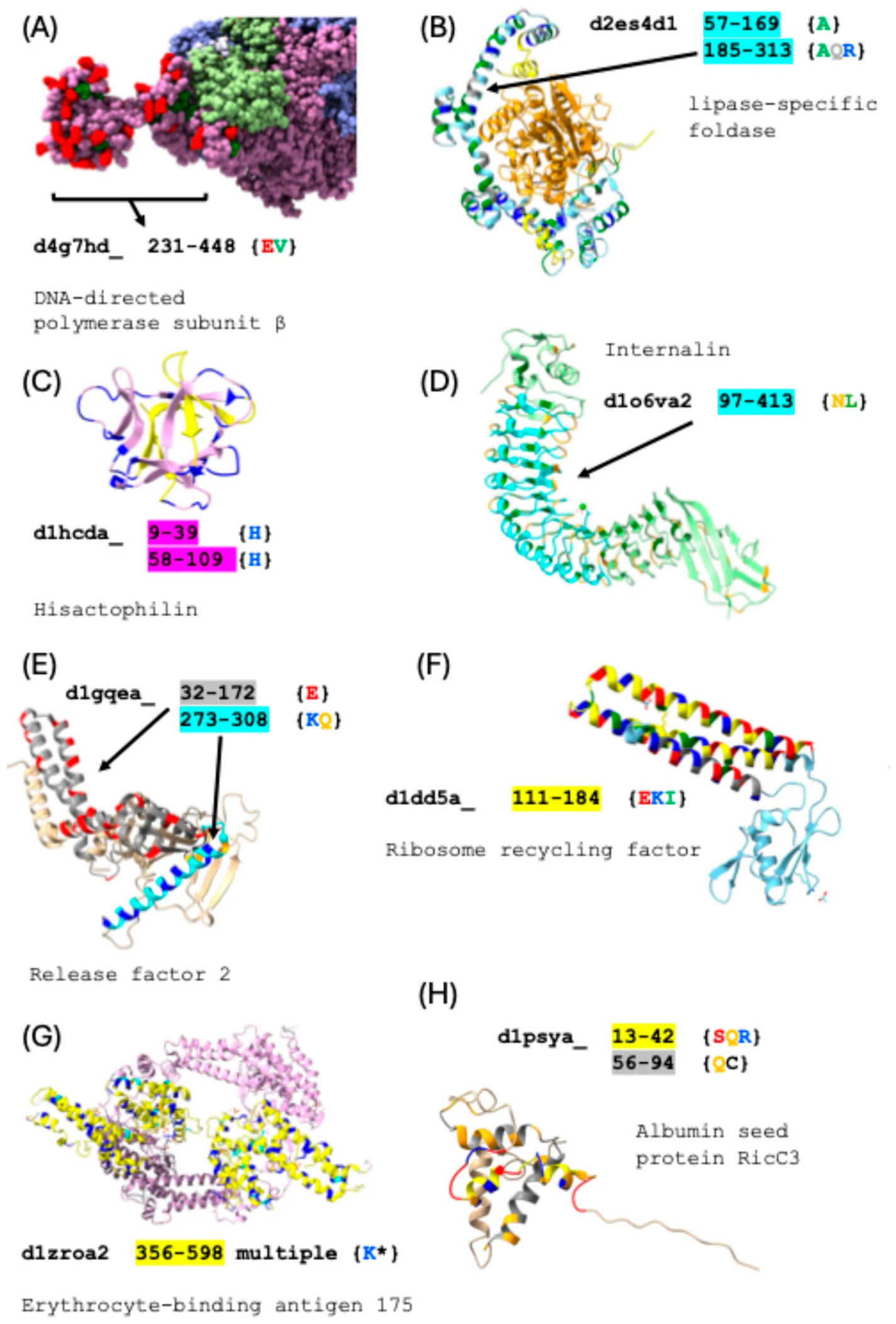
Gallery of long or domain-dominant sCBRs in the domains: (**A**) d4gh7hd, (**B**) d3es4d1, (**C**) d1hcda, (**D**) d1o6va2, (**E**) d1gqea, (**F**) d1dd4a, (**G**) d1zroa2, (**H**) d1psya. All are depicted as ribbon diagrams except for part (**A**), where space-filling is used to better depict the knob shape [60]. The colour coding is different in each panel, but is summarized in the annotating text.

Structural CBRs (sCBRs) are compositionally biased regions within protein domains. Here, specifically, bias-conserved sCBRs are surveyed (Figure 2). Bias conservation was determined as described in *Methods*. In the *astralscopr40R* set that we made, sCBRs are quite frequent, arising in ~1 in 4 (27%) of protein domains. They range in size from trihomopeptides (which are more often linked to intrinsic disorder than to structure [27]), to a 218-residue long {EV}-rich beta-sheet protruding knob in DNA-directed polymerase subunit beta (domain code d4g7hd_) (Figure 3A) [44,45]. For further analysis, the total set of 2185 sCBRs excluding the 129 sidCBRs (i.e., the ones that contain intrinsic disorder) were broken into sets of approximate thirds: LE6 length (≤6 residues, 37%), 7TO16 (7 ≤ length ≤ 16, 30%), and GT16 (length > 16, 32%). LE6 + 7TO16 together are termed LE16. The complete sCBR data set is provided in Supplementary File S1.

sCBRs occur across all the domains of life (Figure 2A). Proteins from humans and model organisms such as *E. coli*, *S. cerevisiae* and *A. thaliana* are prominent in the data set (Figure 2B). Since *astralscop* and the PDB generally are biased to whatever organisms are of greater interest to researchers, organismal prevalences cannot be analyzed statistically. The structure data is dominated by X-ray crystallography (Figure 2C); CBRs with any disorder ascertained from lack of X-ray electron density in the PDB coordinates (termed sidCBRs) are a small minority (~5%) (Figure 2D). Large sCBR subsets are involved in ligand or protein binding, but only 2% were associated with nucleic-acid binding (Figure 2E).

### 3.2. Protein Domains Dominated by Compositional Bias Are Rare (About 1 in 200)

Protein domains that are dominated by compositional bias (over the majority of their length, ≥0.5) are very rare, accounting for 39/1885 cases (2%) (Table 2, termed the Cov_≥0.5_ set). This is 0.5% of the domain data set, or approximately ~1 in 200 of them. This paucity is also evident if one uses the *p*-value threshold of 1 × 10^−10^ that was applied in analyses of prion-like domains [7] (Table 2). (These data exclude the already well-characterized cysteine biases linked to disulphide-rich or metal-ion-binding proteins, as these were set aside at the opening of the Section 2 pipeline.)

Such extreme bias is often associated with protein function (discerned for at least 10/39 cases in the Cov_≥0.5_ set from examination of source references). *Lif* is a lipase-specific membrane-associated foldase with a supporting arginine bias, and a dominant alanine bias enabling its helices to wrap around the lipase in an extended pincer shape (sCBR coverage = 0.86, domain code d2es4d1) (Figure 3B) [46,47]. Hisactophilin (sCBR coverage = 0.70, d1hcda_) is a uniquely histidine-rich protein that binds to actin filaments at acidic pHs, when it becomes polycationic [48] (Figure 3C). Three of the Cov_≥0.5_ set are dominated by lysine- and arginine-rich regions that are implicated in DNA/RNA binding. Two are leucine-rich-repeat (LRR) proteins that are actually more biased for asparagine (sCBR coverage = 0.78 and 0.71, d1jl5a_ and d1o6va2), *Listeria* internalin whose LRR binds around human E-cadherin [49,50], and the cytotoxin YopM from *Yersinia pestis* [51,52] (Figure 3D). Although some of the asparagines in these sCBRs contribute to LRR regular patterning, most are distributed irregularly. The 141-residue {E}-rich sCBR in *E. coli* release factor 2 (coverage = 0.50, d1gqea_) functions partially through RNA mimicry [53,54] (Figure 3E). Similarly, a highly biased glutamate/lysine-rich region (*p*-value = 3 × 10^−13^) in *Thermotoga maritima* ribosome recycling factor (coverage = 0.40, d1dd5a_) functions in the complete mimicry of a tRNA [55,56] (Figure 3F). Previously, it was noticed that structured regions with repetitive D/E tracts were enriched in proteins linked to DNA/RNA functions [57]. Erythrocyte binding antigen 175 from *Plasmodium falciparum* (coverage = 0.75, d1zroa2) binds to the glycoprotein glycophorin A, making use of several {K}-rich alpha-helical substructures [58] (Figure 3G). However, for most members of the *Cov*_≥0.5_ set, the functional significance of the sCBRs is unclear, e.g., albumin seed protein RicC3 has Q- and S-rich tracts (coverage = 0.55, d1psya_) of unknown significance [59] (Figure 3H).

### 3.3. sCBR Types, Secondary Structure and Sequence Conservation

**Figure 4:**
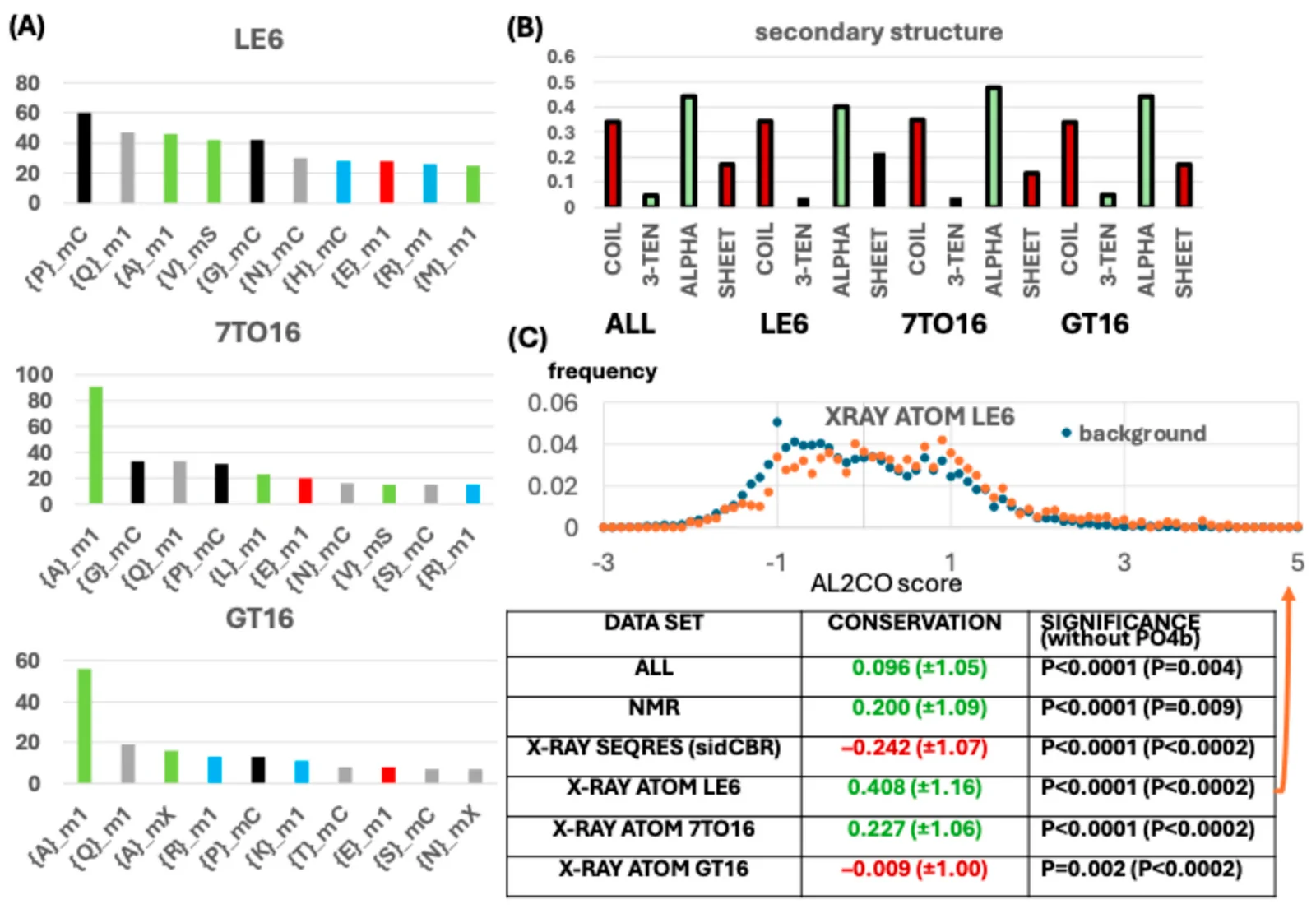
sCBR types, secondary structure, sequence conservation. (**A**) The prevalent sCBR types for the three lengths sets LE6, 7TO16, and GT16, colour-coded green for hydrophobic, blue for positive charge, red for negative charge, grey for polar, and black for proline or glycine. (**B**) Secondary-structure contents for ALL sCBRs, and for the three length sets. They are colour-coded red for significant depletion, green for significant enrichment and black otherwise. (**C**) An example of the distribution of AL2CO conservation scores for sCBRs (for the XRAY ATOM LE6 set), compared to its background distribution. The table lists all the conservation statistics, with *p*-values calculated using a t-test. The bracketed *p*-values are for the same calculation, but with known or potential nucleotide/phosphate-binding cases removed (labelled as ‘PO4b’ sCBRs).

sCBRs are segregated into types depending on length, predominant secondary structure and bias signature. Each is labelled *{X}_mY*, where *{X}* is the bias signature from the fLPS algorithm, and *mY* signifies the predominant secondary structure, taken from the PDB atom records, using the standard secondary-structure codes (1 = alpha-helix, 5 = 3-ten helix, S = beta-strand, C = coil, X = no predominance). sCBR types demonstrate a marked variation in prevalence dependent on length (Figure 4A), with {P}_mC and {Q}_m1 most abundant for length ≤ 6 residues, but with {P}_mC fading away at longer lengths, which are dominated by {A}_m1 biases. Regardless of length, sCBRs are enriched for alpha-helix, and depleted for beta-sheet, indicating that alpha-helices generally accommodate biased sequences better (Figure 4B).

About half of the sCBRs have a LARGE Kingdom multiple sequence alignment demonstrating bias conservation across a majority of the sequences (Supplementary Table S1; four example alignments are provided in Supplementary File S2). A majority of these have mean AL2CO conservation scores > 0.0 (Supplementary Table S1). Such sequence conservation was analyzed for the length sets for sCBRs wholly derived from X-ray crystallographic atomic coordinates, from 2D-NMR atomic coordinates, and also for sidCBRs (i.e., those containing sequence tracts not in the atomic coordinates, indicating intrinsic disorder) (Figure 4C). Contrary to the general conception that CBRs are less conserved, stemming from the LCR/CBR association with intrinsic disorder [10,61], shorter examples (≤16 residues) are actually significantly highly conserved (Figure 4C). It is only cases > 16 residues or the sidCBRs that are significantly less conserved than background. The longer sCBRs thus maintain biases despite less conservation of specific sequence patterns.

sCBRs were analyzed for clade-specific shifts in sCBR size, by counting changes in the number of primary biasing residues gained or lost within the sCBRs, or in an interval bounded by buffers of the same length as the sCBR at either end (see *Methods* for further detail). Only clades with ≥5 members in the multiple sequence alignments are considered.

In general, about half of the time there are clade-specific shifts in sCBR bias (Supplementary Table S2). These are generally expansions/enrichments of the sCBR with more biasing residues (~40% of sCBRs), with contractions/depletions being much less common (~8% generally of sCBRs). These results also indicate that the sCBRs boundaries are largely optimized by the algorithm, since a signal for rare contractions/depletions is discerned.

### 3.4. General Trends in Ligand and Protein Binding

**Figure 5:**
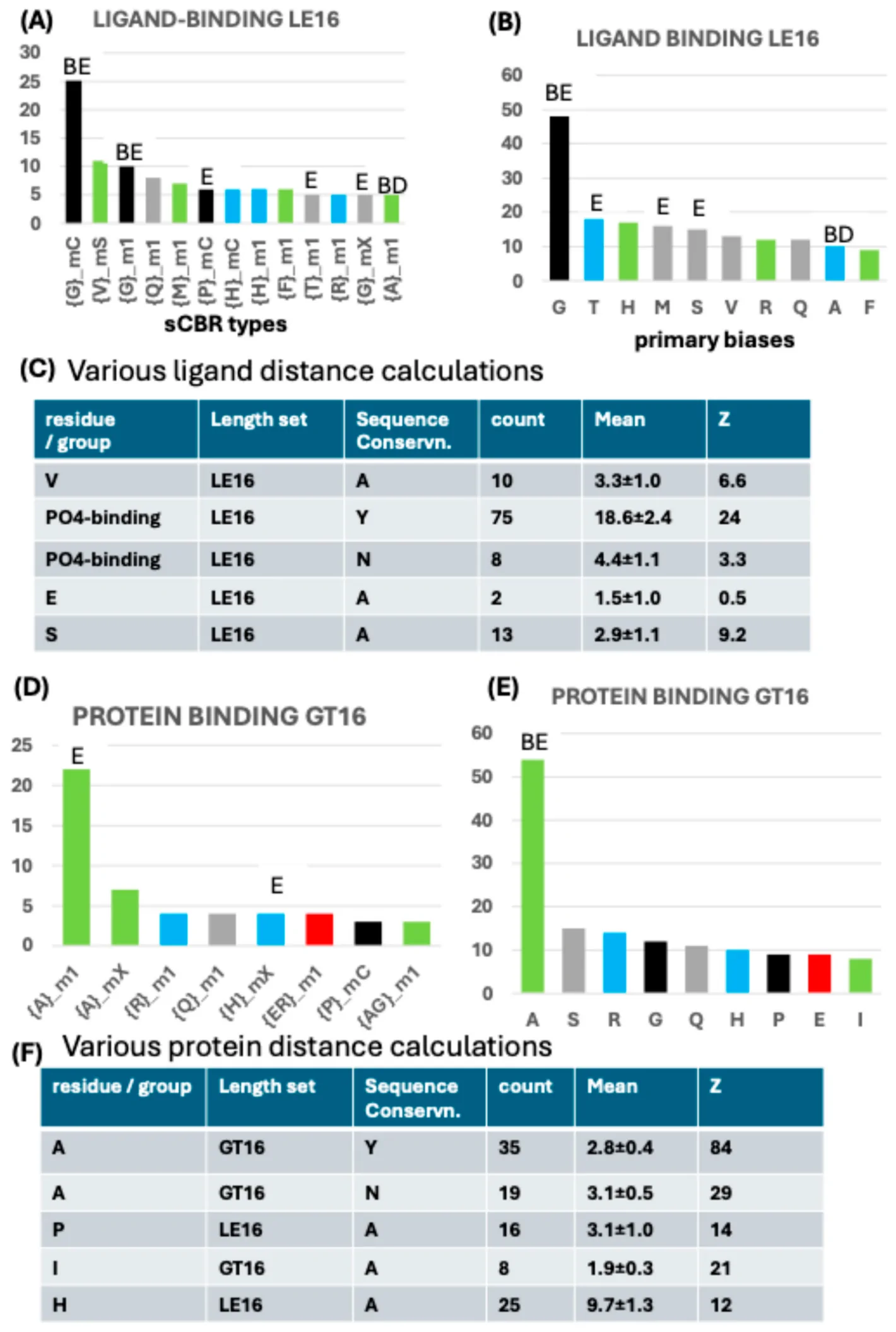
Ligand and protein binding. (**A**) For ligand binding, the prevalence of sCBR types in the length set LE16. They are labelled *{X}_mZ*, where X is the bias signature and Z is the predominant secondary-structure type (1 = alpha-helix, S = beta-strand, C = coil, X = no predominance). Enrichments are labelled E, or BE if significant after Bonferroni adjustment, and depletions are labelled D, or BD. (**B**) For ligand binding, the prevalence of primary residue biases for sCBRs in the length set LE16. Same format as part (**A**). (**C**) Some examples of the significance calculation for ligand binding. PO4b is the set of sCBRs that contact a nucleotide or phosphate-containing ligand, or a nucleotide analogue. For sequence conservation, ‘Y’ indicates yes, ‘N’ indicates no, and ‘A’ indicates ‘all’ (i.e., it is not considered). *Mean* is the expected number from the distance sampling calculations and Z is the z-score for the *count* relative to the *Mean*. More listings are in Supplementary Table S3. (**D**) For protein binding, the prevalence of sCBR types in the length set GT16. Same format as part (**A**). (**E**) For protein binding, the prevalence of primary residue biases for sCBRs in the length set LE16. Same format as part (**A**). (**F**) Some examples of the significance calculation for protein binding. Table format is the same as (**C**). More listings are in Supplementary Table S3.

Glycine-rich sCBRs are significantly enriched near ligands, due to many phosphate- or nucleotide-binding motifs (Figure 5A,B, Supplementary Table S3). However, in general sCBRs are significantly enriched near ligand-binding sites in structures, even if these nucleotide/phosphate-binding or glycine-rich cases generally are removed from the data (Figure 5C,D, Supplementary Table S3, part B). Also, the high levels of sequence conservation for sCBRs are maintained if such cases are removed (Figure 4C above).

Longer protein-binding sCBRs are enriched in alanine residues (the *GT16* length set, Figure 5E,F, Supplementary Table S3, part D). Alanine-rich alpha-helical regions are particularly prominent (Figure 5E). Their location in protein interfaces is also highly significant spatially (Supplementary Table S3, part F). However, there is one notable depletion, i.e., of valine-rich protein-binding sCBRs amongst the LE16 set, there being only two (both {V}_mS) (Supplementary Table S3, part C). This suggests that valine-rich areas are not easy to design into protein interfaces.

**Figure 6:**
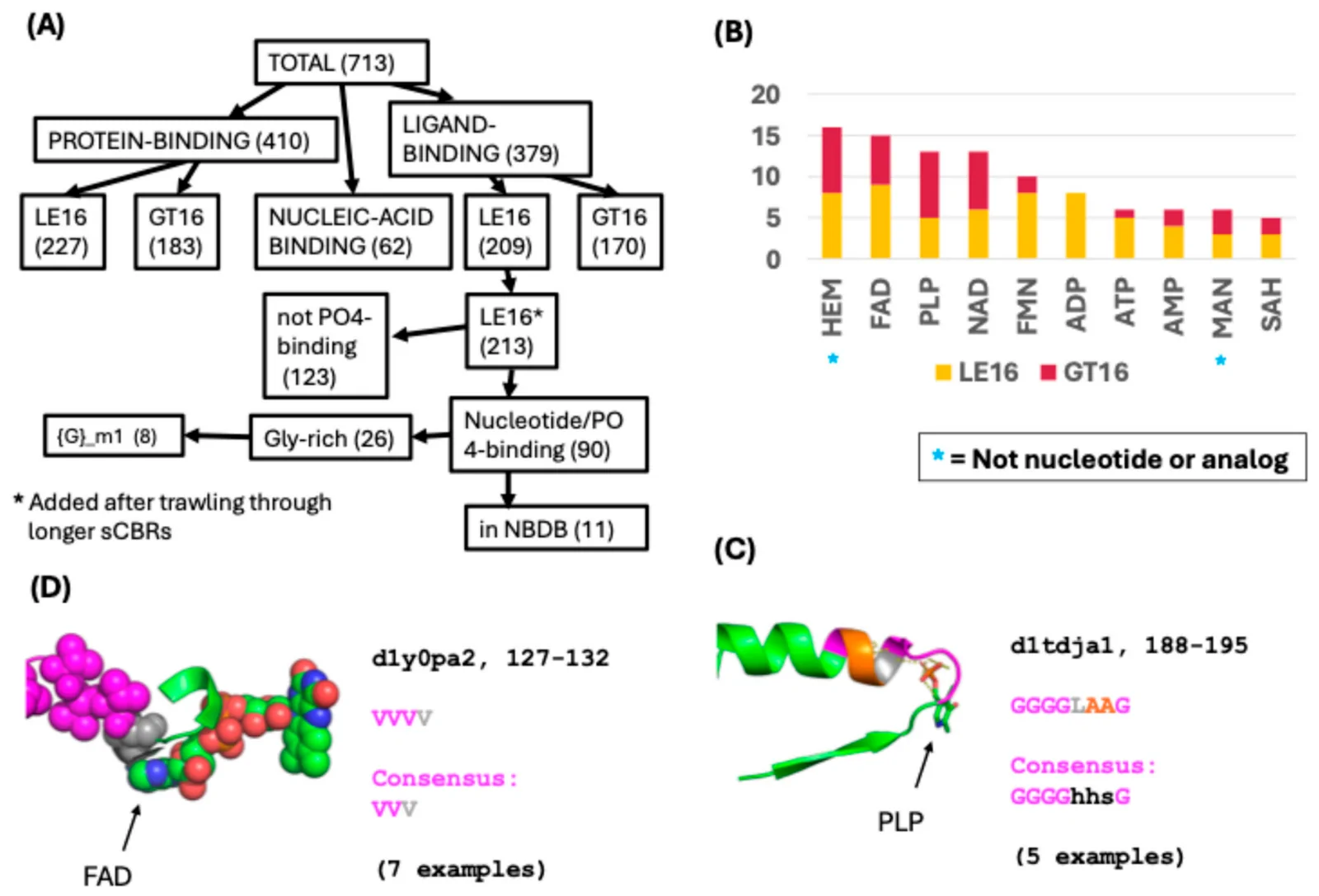
Ligand binding, particularly nucleotide/phosphate binding. Parts C and D are drawn using PyMOL (version 2.6) (http://pymol.org). (**A**) A hierarchical tree depicting the types of binding for sCBRs. (**B**) The occurrences of sCBR ligands in the LE16 and GT16 data sets. (**C**) Examples of a {G}-rich sCBR in a nucleotide-binding site. Consensuses are depicted. The letter h = hydrophobic, and s = small residue (according to the Taylor classification [74]). (**D**) The {V}-rich sCBR occurring at nucleotide-binding sites. A consensus is depicted.

Binding types and subtypes were schematized into a tree format (Figure 6A). We can clearly see that the most common ligand types are nucleotides or nucleotide analogues (Figure 6A,B). The most common individual ligand is heme, although the sCBRs involved in its binding are diverse in bias (Figure 6B). About 29% (26/90) of the nucleotide-binding motifs are glycine-rich (Figure 6A). In general, for sCBRs, glycine bias and/or phosphate binding are significantly associated with increased usage of backbone hydrogen bonds to ligands, as determined by Fisher’s exact tests (*p* ≤ 0.000006; Table 3A,B). Glycine residues do not shield the polypeptide backbone with a sidechain, making it more easily available for ligand binding through peptide hydrogen bonds. These data demonstrate this role of glycine clearly.

### 3.5. sCBRs That Are Mostly Nucleotide/Phosphate Binding

sCBRs involved in nucleotide binding (NB-sCBRs) were cross-referenced with the NBDB, an online database of nucleotide-binding motifs in proteins [62]. Despite this database being over ten years old, we still find 11 of the NB-sCBRs overlapping motifs in it, five of them being of the type *{G}_m1* (Figure 6A).

Examples of sCBRs that coincide with known nucleotide-binding sites are depicted in Figure 6C,D. The {G}_m1/mX sCBR containing a row of four glycines is observed in three pyridoxal-5′-phosphate-binding sites and two other cases unbound to ligands but superimposable. The example is from *E. coli* threonine deaminase (d1tdja1) [63,64]. The {V}_mS sCBR in Figure 6D is one of seven cases with a core VVV tripeptide, all of which are nucleotide binding (diverse nucleotides including small-molecule codes UD2, AHX, FAD and PLP). One of the valines abuts a hydrophobic patch on the nucleotide and forms an interlocking packing pattern with the other two valines behind it. An example from flavocytochrome C3 bound to FAD is illustrated (d1y0pa2) [65].

To check generally for pattern words in the sCBRs linked to ligand binding, they were clustered according to their patterns of biasing residues (with any other residue relabelled ‘X’) and secondary-structure strings (Supplementary Table S4), cases that are embedded in longer sCBRs were added in, and then pattern words with count ≥ 3 were checked for identical or similar ligands that are close to equivalent amino-acid residues. Nucleotide binding {G} and {V}-rich motifs discussed above are recovered as sequence word embeddings in several cases, with all cases binding nucleotides or nucleotide analogues.

**Figure 7:**
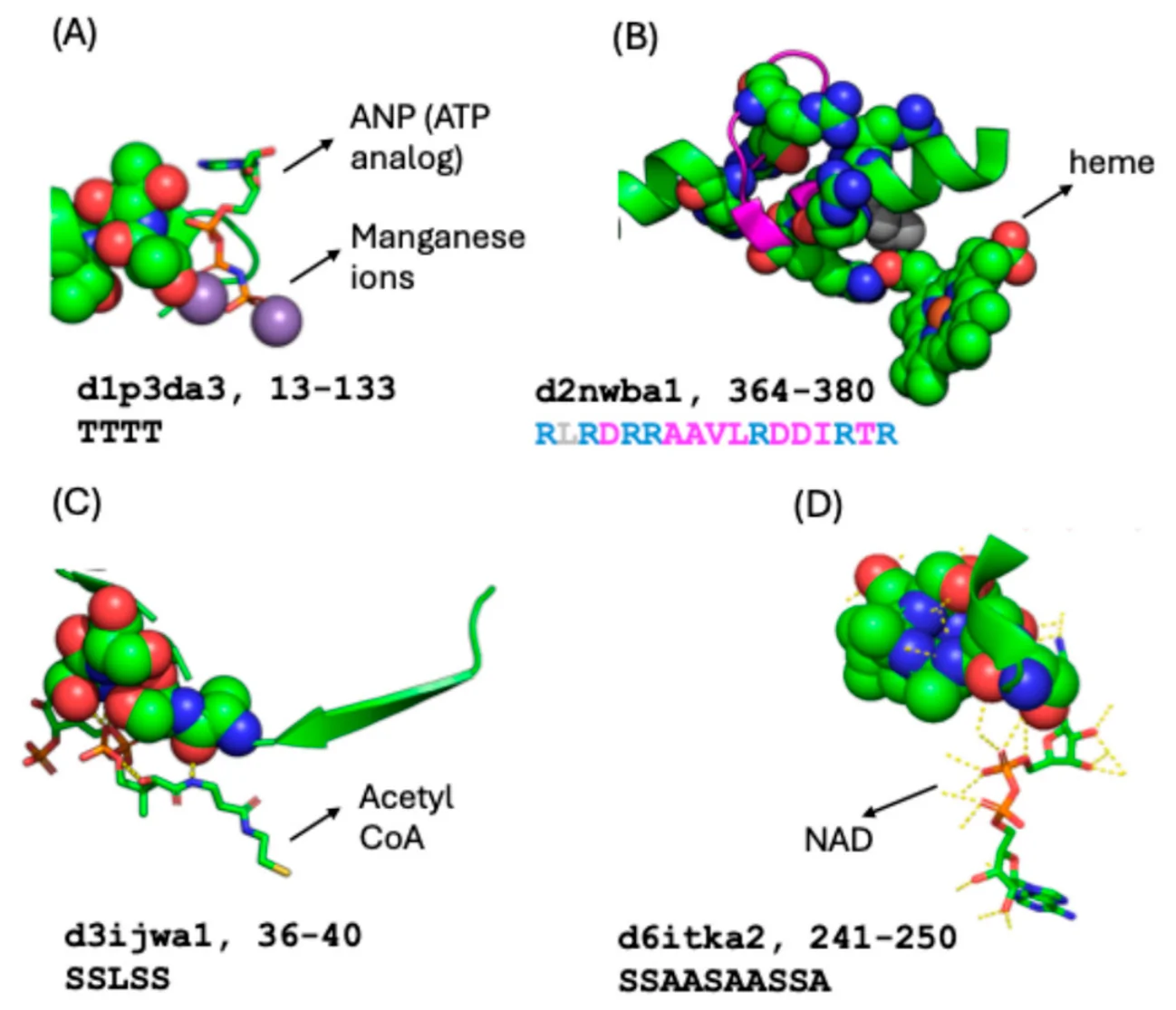
Further ligand-binding sCBR examples from the following domains: (**A**) d1p3da3, (**B**) d2nwba1, (**C**) d3ijwa1 and (**D**) d6itka2. These are drawn using PyMOL (version 2.6) (http://pymol.org).

Other examples of sCBRs do not conform to a specific motif, but they feature the same ligand-binding sidechain interactions. There are five cases of short {T}_m1 (mostly alpha-helical) sCBRs (in the LE16 data set) involved in ligand binding (four nucleotides or analogues of them, and one other ligand). They all involve hydrogen bonding of threonine hydroxyls or threonine backbone nitrogens to the ligand. Depicted is an example from an alanine ligase (domain code 1p3da) binding ANP, a nucleotide analog [66,67] (Figure 7A). {R}-rich sCBRs are most commonly linked to nucleotide binding (seven cases), but there are also three {R}_m1 cases involved in heme binding. They form arginine clusters at or near hemes and may have a role in proton movement and maintaining redox potential. The example shown in Figure 7B is at a heme-binding site in 2,3-dioxygenase from *Shewanella oneidensis* (2nwba1) [68]. Longer (GT16) {S}-rich sCBRs are most often sugar binding (four cases), e.g., one example that binds multiple mannose molecules (d1gaia_ [69,70]), whereas the shorter cases (LE16) are mostly nucleotide binding (or nucleotide-analog binding; seven cases total). Two examples with multiple serines involved in nucleotide binding are illustrated, an Acetyl-CoA binding site in an aminoglycoside N-acetyltransferase from *Bacillus anthracis* [71,72] (Figure 7C,D), and the {AS}-rich sCBR at the NAD-binding site in bacterial malate dehydrogenases (d6itka2, from *Corynebacterium glutamicum* [73]).

### 3.6. Evolutionary Dynamics of Nucleotide/Phosphate-Binding sCBRs

Nucleotide/phosphate-binding motifs formed from simple, biased sequences (primarily {G}-rich) have been noted for decades [62,75,76]. Such simple glycine-rich sequences typically sit at the N-terminus of an alpha-helix and are hypothesized to have an intrinsic propensity for it [77]. The degree to which these have arisen by convergence or divergence and how often during evolution is not completely clear [62,76,78,79,80]. To gain further insight into the evolutionary dynamics of NB-sCBRs and nucleotide/phosphate-binding motifs generally, shifts in counts of biasing residues were probed. Supplementary Table S2 indicates that {G}-rich or nucleotide-binding motifs undergo defined clade-specific gains of glycine about half of the time (42–54%) across LARGE alignments (or KERNEL alignments if there is no LARGE one), indicating continued evolutionary sampling of sCBR variants. However, losses of glycines in sCBRs occur only a third as frequently as expansions, demonstrating that removal of glycine residues in a motif, once they have arisen, is more difficult.

### 3.7. {A}-Rich Protein-Binding Examples

**Figure 8:**
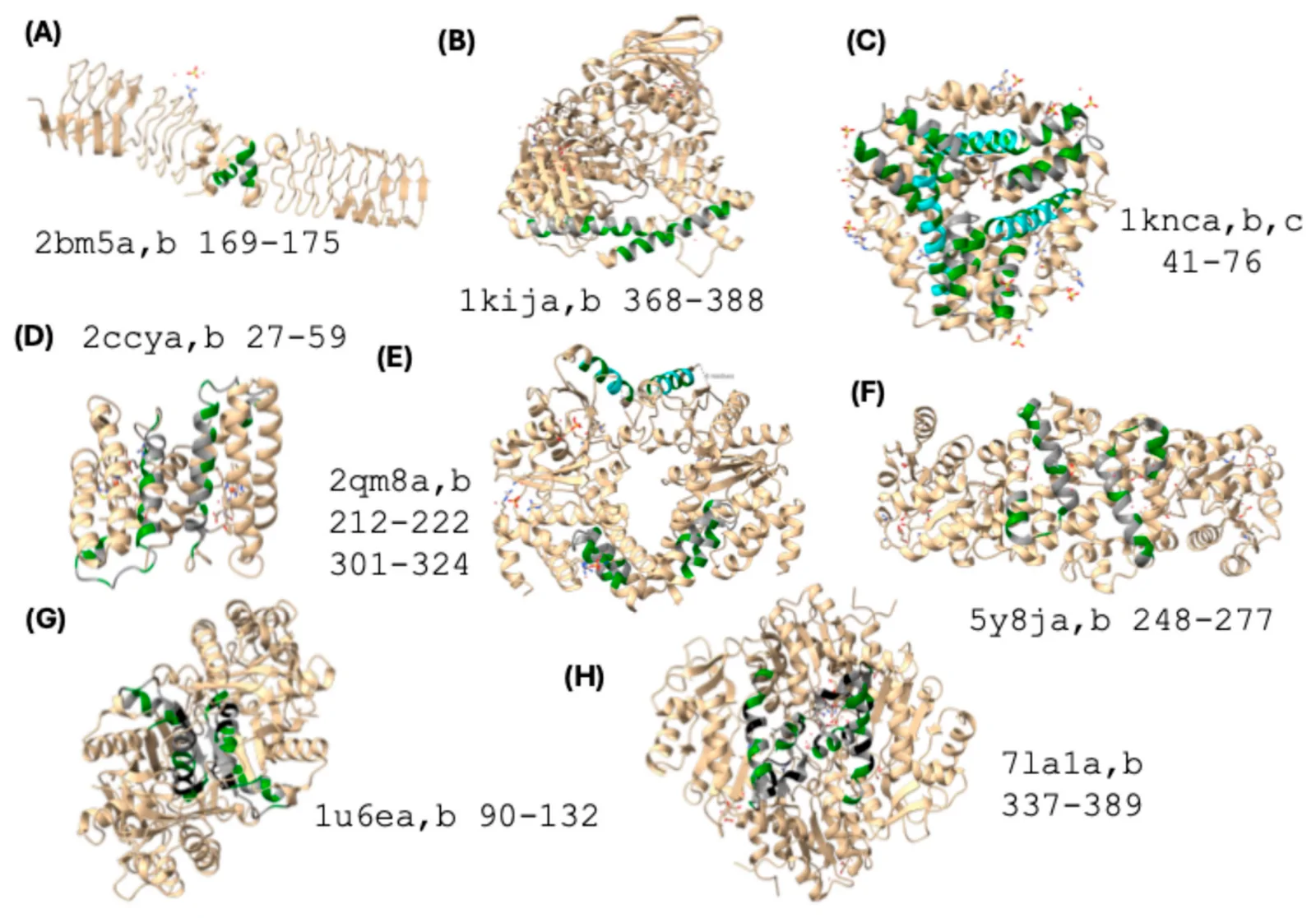
Gallery of alanine-rich protein-binding sCBRs from the following domains: (**A**) 2bm5a, (**B**) 1kija, (**C**) 1knca, (**D**) 2ccya, (**E**) 2qm8a, (**F**) 5y8ja, (**G**) 1u6ea and (**H**) 7la1a. These are drawn using ChimeraX (version 1.12) [60]. The {A}-rich sCBRs are coloured in grey, with green for alanine residues, and black for glycines. Secondary shorter {A}-rich sCBRs are coloured cyan instead of grey.

The most prominent feature of longer protein-binding sCBRs noted above is alanine bias. Several of these {A}-rich interfacial sCBRs were assembled in a gallery (Figure 8). These are all homodimers and one homotrimer. For interfaces enriched in alanines, the interface topography can be more determined by the protein backbone, and might enable tighter hydrophobic packing (e.g., Figure 8A). The alanine content may stabilize more extended, less globular substructures (such as in Figure 8B,E or *Lif* in Figure 3B), or maintain alpha-helical structure pointing away from the interface, while other sidechains maintain interaction specificity (e.g., Figure 8D or Figure 8F).

### 3.8. Nucleic-Acid Binding sCBRs

Of the small population of nucleic-acid binding sCBRs (37 cases), about half are positively charged (46%) (Supplementary File S3A,B), to counteract the negative charge of DNA/RNA [81,82], such as the arginine-rich bridge helix of the Cas9 DNA-cutting enzyme, which interacts with the guide RNA and target DNA (domain code d4oo8a3) [83,84]. However, there are also several negatively charged cases that are more peripheral, and may function in steering binding (Supplementary File S3C).

### 3.9. Intrinsically Disordered sCBRs Inside Protein Domains (sidCBRs)

Compositional bias is most commonly associated in the literature with intrinsic disorder in proteins generally [8,85]; however here, only ~5% of cases contain intrinsic disorder (Figure 2D). Of the sidCBRs (129 examples), about a quarter are fully disordered (26%), and the vast majority are <40 residues in length (93%), a significant enrichment of shorter ones relative to the general sCBR population (percentage = 85%, *p* = 0.004 for hypergeometric probability), which is surprising since generally intrinsic disorder is associated with protein length changes during evolution [86,87] (Supplementary Figure S1C–E). Charged sidCBRs, particularly negatively charged ones, and short glycine-rich ones (all ≤ 13 residues), are the most numerous. The longest negatively charged case that is mostly disordered is a {D}-rich sCBR in RNA polymerase I subunit RPA190 (domain code d4c2ma_). It contains the DNA-mimicking loop of this protein and an adjacent short alpha-helix. The DNA-mimicking loop occludes the binding sites of nucleic acids, regulating their interaction [88,89].

Other sCBRs with D or E primary bias may be implicated in binding modulation, such as the {D}_mD sCBR near the active site of PtpB tyrosine phosphatase from *M. tuberculosis* (d1ywfa1) [90,91], and an {E}_mD sCBR in the hinge region of aspartyl tRNA synthetase, possibly involved in steering binding to the ribose phosphate backbone of the tRNA (d4j15a2) [92,93]. Others feature in domains that have several disordered loops, but their function has not been dissected, e.g., a {D}_mD sCBR in CAND1 protein which serves in the assembly of ubiquitin ligase complexes (d1u6gc_) [94,95].

### 3.10. sCBRs in Transmembrane Proteins

The sCBR data set was checked for transmembrane protein membership. Only 1.1% (21/1904) of sCBR-containing protein domains are transmembrane, compared to 2.2% of *astralscop40R* overall, which is a significant depletion according to hypergeometric probability (*p* = 0.00013). There are 27 sCBRs in transmembrane proteins, 15 of which are hydrophobic primary biases (either L, W, F or A), with five F and four L within transmembrane helices being the most common.

## 4. Conclusions

The complexity or information entropy of protein sequence tracts has been researched extensively, and has been an important consideration in the filtering out of false positives during sequence alignment. Lower sequence complexity might naturally arise due to the tendency of homopeptides and repetitive tracts to expand and contract more readily during unequal crossing-over in meiosis, or errors in DNA replication. Such sequence complexity per se is unlikely to be under selection, since it is a distributed property of sequence tracts that originated from algorithmic considerations. However, compositional biases for a subset of amino acids (such as glutamine and asparagine in prion-like domains) may have functional import. As described in the Introduction, previous work on protein domains has focused on analyzing regions with lower sequency complexity, rather than compositional biases, and has not considered bias conservation and partner binding as functional filters.

Acting on the hypothesis that compositional bias is per se more functionally meaningful than sequence complexity, we set about documenting the wide diversity of compositionally biased regions in structured protein domains. This was probed using a novel strategy applying varied annotation parameter sets. This strategy works here for protein domains partly because they are already delimited by their structural integrity. Domains dominated by bias are rare (~0.5%) but often offer dramatic evolutionary solutions to functional problems. The functional relevance of sCBRs was demonstrated through analysis of conservation and partner binding. Contrary to general assumptions about biased regions, shorter sCBRs are often implicated in highly sequence-conserved functional roles, even when the common nucleotide-binding-linked sCBRs are set aside. New insights into documented ligand-binding subsequences were revealed, such as the marked tendency of glycine-rich nucleotide-binding motifs to gain rather than lose glycines clade-specifically, and evidence for the key role of the glycines in such motifs in enabling backbone hydrogen bonding to the ligand. The sCBR data might be useful for informing protein design/engineering experiments.

## Figures and Tables

**Table 1:** List of the fLPS parameters used.

Notional Parameter Set Label *	fLPS Parameters (m, M, t)			Actual sCBR Frequencies in Structured Domains (Total Domains = 7112) **
Len = 6_Cov = 5%	5	9	1.3 × 10^−4^	529 (7.4%)
Len = 7_Cov = 2%	5	7	8.6 × 10^−6^	2 (0.0%)
Len = 7_Cov = 5%	5	9	1.0 × 10^−4^	0 (-)
Len = 15_Cov = 2%	8	10	5.1 × 10^−7^	3 (0.0%)
Len = 15_Cov = 5%	10	14	1.7 × 10^−5^	9 (0.1%)
Len = 15_Cov = 10%	7	17	3.5 × 10^−5^	839 (11.8%)
Len = 25_Cov = 2%	11	13	1.5 × 10^−8^	2 (0.0%)
Len = 25_Cov = 5%	14	18	1.8 × 10^−6^	4 (0.0%)
Len = 25_Cov = 10%	12	22	9.8 × 10^−6^	176 (2.5%)
Len = 25_Cov = 25%	11	34	4.4 × 10^−4^	229 (3.2%)
Len = 25_Cov = 40%	8	53	5.5 × 10^−4^	352 (4.9%)
Len = 50_Cov = 2%	16	18	2.3 × 10^−12^	0 (-)
Len = 50_Cov = 5%	21	25	6.2 × 10^−9^	1 (0.0%)
Len = 50_Cov = 10%	23	33	4.1 × 10^−7^	9 (1.3%)
Len = 50_Cov = 25%	20	50	4.7 × 10^−5^	16 (2.2%)
Len = 50_Cov = 40%	15	76	1.6 × 10^−4^	98 (1.4%)

* Len = x_Cov = y% means region target length of *x* and data set coverage of *y*%, i.e., overall percentage of *whole* protein sequence residues from a benchmark set taken from UniProt, not structured domains per se [7,8]. ** Each sCBR was labelled with the lowest coverage and shortest target length parameters that find it. These counts are the final bias-conserved sCBR data set. The domain total is for the *astralscop40R* set derived as described in Section 2.1.

**Table 2:** Domains dominated by compositional bias are rare.

Subset *	Cases	Percentages of CBR-Containing Domains *(of All Domains)*
Total of CBR-containing domains	1885	100% *(26.5%)*
Domain coverage ≥ 0.5 (subset *Cov_≥0.5_*)	39	2.1% *(0.5%)*
Domain coverage ≥ 0.33 (subset *Cov_≥0.33_*)	198	10.5% *(2.8%)*
−Log(bias *p*-value) ≥ 10.0 (subset −*LogP_≥10.0_*)	44	2.3% *(0.6%)*
−Log(bias *p*-value) ≥ 8.0 (subset −*LogP_≥8.0_*)	157	8.3% *(2.2%)*
*Cov_≥0.5_* ∩ −*LogP_≥10.0_*	8	0.4% *(0.1%)*
*Cov_≥0.33_* ∩ −*LogP_≥10.0_*	23	1.2% *(3.2%)*
*Cov_≥0.5_* ∩ −*LogP_≥8.0_*	18	1.0% *(0.3%)*
*Cov_≥0.33_* ∩ −*LogP_≥8.0_*	69	3.7% *(1.0%)*

* The bias *p*-value is the lowest one for any one sCBR in the domain.

**Table 3:** Ligand hydrogen bonding.

**(A). Counts for different subsets**			
**Group/Residue**	**Forming Hydrogen Bonds (HBs)**		
	**All HBs**	Backbone HBs *	**Glycine-only Backbone HBs**
All LE16 (total sCBRs 209) *	66 (32%)	41 (20%)	24 (11%)
PO4/nucleotide binding (LE16)	42 (20%)	34 (16%)	19 (9%)
{G}_m1/{G}_mC	18 (9%)	17 (8%)	14 (7%)
G primary bias (glycine-rich)	29 (14%)	26 (12%)	22 (11%)
Not PO4/nucleotide binding (LE16)	24 (11%)	7 (3%)	5 (2%)
**Backbone HB counts**			
		**All backbone**	**Backbone to Gly**
All sCBRs (total HBs 570)		203 (36%)	92 (16%)
All LE16 (total HBs 214)		113 (53%)	67 (31%)
**(B). Contingency tables for Fisher’s exact tests †**			
	**PO4/nucleotide binding**		**PO4/nucleotide binding**
**Have Backbone HBs**	55		25
**Have No Backbone HBs**	24		48
	**Glycine-rich**		**Not glycine-rich**
**Have Backbone HBs**	26		54
**Have No Backbone HBs**	3		69

* All H-Bonds observed involve backbone N. † The association of backbone hydrogen bonding with PO4/nucleotide-binding motifs, and also with glycine-rich motifs was analyzed for these 2 × 2 contingency tables extracted from this data, with Fisher’s exact text. The two-tailed *p*-values were 0.00002 and 0.000006 respectively.

## Data Availability

The original contributions presented in this study are included in the article/Supplementary Material. Further inquiries can be directed to the corresponding author.

## Supplementary Materials

The following supporting information can be downloaded at https://www.mdpi.com/article/10.3390/biom16091222/s1. Figure S1: sidCBRs; Table S1: sequence conservation; Table S2: clade-specific changes; Table S3: partner binding; Table S4: pattern words; File S1: sCBRs; File S2: MSA examples; File S3: nucleic-acid binding.

## Abbreviations

The following abbreviations are used in this manuscript:

CBR	Compositionally biased region
sCBR	Structural CBR
sidCBR	Structural intrinsically disordered CBR
LCR	Low-complexity region
IDR	Intrinsically disordered region
PDB	Protein DataBank
MSA	Multiple sequence alignment
LRR	Leucine-rich repeat
NB	Nucleotide binding
