# Supplementary material for: Compositionally Biased Regions Within Structured Protein Domains
Source: Biomolecules. 2026 Aug 22;16(9):1222. doi: 10.3390/biom16091222 (PMC13604049; doi:10.3390/biom16091222)
Supplement: Supplementary file 1 [file biomolecules-16-01222-s001.zip › Suppl-Figure-S1-sidCBRs.pdf]

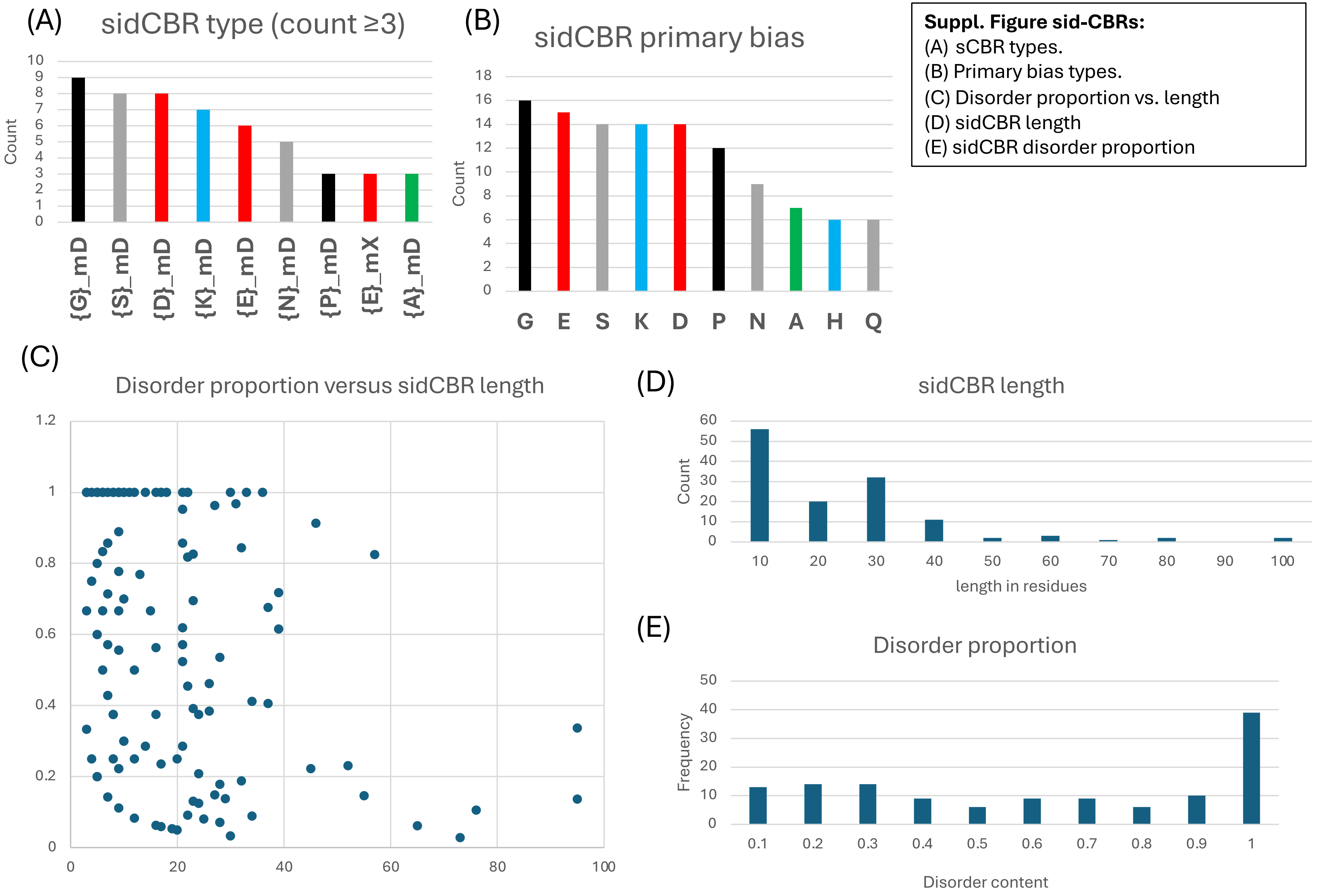

Suppl. Figure sid-CBRs:
(A) sCBR types.
(B) Primary bias types.
(C) Disorder proportion vs. length
(D) sidCBR length
(E) sidCBR disorder proportion
