# Supplementary material for: Compositionally Biased Regions Within Structured Protein Domains
Source: Biomolecules. 2026 Aug 22;16(9):1222. doi: 10.3390/biom16091222 (PMC13604049; doi:10.3390/biom16091222)
Supplement: Supplementary file 1 [file biomolecules-16-01222-s001.zip › Suppl-File-S2-MSA-examples.pdf]

Suppl File S2: Examples of Multiple Sequence Alignments for sCBRs

These are LARGE Kingdom alignments.

The sCBR ID is:

ASTRALDomainID_BiasSignature_Start_End_BiasPValue

The sCBRs are highlighted in **pink**.

(A) **d6m0qa__{P}_411_419_2.040e-05** mean AL2CO score = 0.35(±0.90)

```
d6m0qa_        nrpnppepekpgfgiftqlfwskgnnpas
Q50925         NRPNPPEPEKPGFGIFTQLFWSKGNNPAS
F9ZIJ1         NRPAPPAPEKEGYAYFAQLFWSKGNSPAA
Q2YA36         NRPTPLPPDKEMFAGFTQLYWSKDNNPAA
Q223N2         ------------------------PMDAK
A0A1W6SRU0     NRPAPMAPDKEMFAGFTQLYWSKDNNPAA
A0A0F7KIN0     NRPAPPAPDKPGFGQFTQLFWSKGNNPAS
A0A1I4XAV9     NRPTPLAPDKEMFAGFTQLYWSKDNNPAA
A0A1K1P0I0     NRPLPLPPDKEMFAGFTQLYWSKDNNPAA
M5DDF6         NRPAPPAPEKPGFAQFSQLFWSKGNNPAS
A0A1H7S0H0     NRPLPLPPDKEMFAGFTQLYWSKDNNPAA
A0A1H3PZT5     NRPVPPEPEKPGFGIFTQLFWSKGNNPAS
A0A1G5J0N2     NRPAPMAPDKEMFAGFTQLYWSKDNNPAA
A0A1G5SG74     NRPAPPEPEKPGFHIFTQLFWSKGNNPAS
A0A1H9SCG6     NRPNPPEPEKPGFMIFTQLFWSQDNNPAS
A0A1G4ZDW7     NRPAPMAPDKEMFAGFTQLYWSKDNNPAA
A0A1I3BHD1     NRPLPLPPDKEMFAGFTQLYWSKDNNPAA
A0A1H8VHN7     NRPAPPAPEKEGYAYFAQLFWSKGNSPAA
A0A1I2H304     NRPDPPPPEKPGFAIFSQLFWSKGNNPAS
```

A0A1I4HHU7 NRPNPPAPEKPGFMIFSQLFWSQDNNPAS
A0A1H3R5V0 NRPAPPAPEKPGFGQFTQLFWSKGNNPAS
A0A2V3VDT8 NRPNPPAPEKPGFGIFSQLFWSKANNPAS
A0A4R1BIY1 ------------------------PFDEK
A0A5B8RX05 NRPALPAPEQDEPGGFFSLFFAQKNQTTL
A0A840BPK5 ------------------------PFDEK
A0A916J4W1 ------------------------PFDEK

(B) **d2q4ha1_{SM}_155_168_3.261e-06 mean AL2CO score = 1.04(±0.63)**

d2q4ha1 --------------vfknqgasagasmshshsqmmalpvv--------------------
Q9FK51 --------------VFKNQGASAGASMSHSHSQMMALPVVPPTVSSRLDGTKDYFEETGK
V7C4K9 --------------VFKNHGASAGASMSHSHSQMMALPIIPPTVSARLASMKDHFDQTGK
A0A072U914 --------------VFKNHGAAAGASMSHSHSQMIALPIIPPSVSARLGSMKEYFDQTGK
A0A3Q7IH79 -------------QVFKNHGASAGASMSHSHSQMIALPIVPPTVSARLDSMMEYYKQTGK
B9IPP8 --------------VFKNHGASAGASMSHSHSQILALPIIPSSVSARFEGMNEYFEKTGK
M5W2M7 --------------VFKNHGASAGASMSHSHSQIMALPVVPPAVATRLGSMKEYFDQTGK
B9SHG4 --------------VFKNHGASAGASMSHSHSQIMALPIIPPSVSARLDGTKEYFDNTGK
D7LXS5 --------------VFKNQGASAGASMSHSHSQIMALPVVPPTVSSRLDGTKDYFDETGK
I1MNU2 --------------VFKNHGASAGASMSHSHSQMMALPVIPPSVSARLTSMKDHFDQTGK
A5C6V7 --------------VFKNHGASAGASMSHSHSQIMALPIVPPTVSSRLDCMKGYFEETGK
M1AEW4 -------------QVFKNHGASAGASMSHSHSQMIALPIVPPTVSARLDSMMEYYKQTGK
M4CD60 --------------VFKNHGASAGASMSHSHSQIMALPVVPPTVSSRLDGTKSYFEETGK
S8C1H7 -------------QVFKNHGASAGASMSHSHSQIMALPIIPPTVSARIHSMKEHFERSGK
A0A061F9R4 --------------VFKNHGASAGASLSHSHSQILSLPVVPPSVSARIGSMKEYFNQTGK
A0A067EMC2 --------------VFKNHGASAGASMSHSHSQLLALPVIPPTISARINSTKEYFDQTGK
A0A067KUD1 --------------VFKNHGASAGASMSHSHSQIIALPVIPPSVFARLDGTKEYFDKTGK
A0A078GG55 --------------VFKNHGASAGASMSHSHSQIMALPVVPPTVSSRLDGTKDYFEETGK
A0A087G856 --------------VFKNHGASAGASMSHSHSQIMALPVVPPTVSSRLNGTKDYFDETGK

```
R0FC90        --------------VFKNHGASAGASMSHSHSQIMALPVVPPTVSSRLDGTKEYFDETGK
A0A0A0L3Q5    --------------VFKNHGASAGASMTHSHSQIVGLPVIPPSVTTRLDSMKQYFNQTGK
W9RRZ1        -------------QVFKNHGVSAGASMSHSHSQMLALPIVPPTVSARLDSMKEYFDQTQK
V4TTA4        --------------VFKNHGASAGASMSHSHSQLLALPVIPPTISARINSTKEYFDQTGK
V4KV20        --------------VFKNQGASAGASMSHSHSQIMALPVIPPTVSSRLDGTKDHYEETGK
A0A058ZT74    -------------QVFKNHGASAGASLSHSHSQIIALPIVPSNVSARIECLREHFNQTGK
A0A022RJY0    -------------QVFKNHGASAGASMSHSHSQIMALPIIPPTASARIDSTKEYFAQTGK
A0A0D3EEI7    --------------VFKNHGASAGASMSHSHSQIMALPVVPPTVSSRLDGTKDYFEETGK
A0A0D2SST9    --------------VFKNHGASAGASMSHSHSQMLSLPIVPPSVSTRLNSMKEHFNQTGK
A0A0L9VKN1    --------------VFKNHGASAGASMSHSHSQMMALPIIPPSVSARLASMKDHFDQTGK
A0A151T4Z3    --------------VFKNHGASAGASMSHSHSQMMALPIIPPSVSARLASMKDYFDQTGK
A0A165YBK9    --------------VFKNHGASAGASMSHSHSQMIALPVIPPTVSARIDSMKEHFDQTGK
A0A1S4C1U8    -------------QVFKNHGASAGASMSHSHSQMIALPIVPPAVSARLDSMREYYKQTGK
A0A1S2XI76    --------------VFKNHGASAGASMSHSHSQLLALPVIPPSVSARLASMKDFFDQTGK
A0A1S3VIR6    --------------VFKNHGASAGASMSHSHSQMMALPIIPPSVSARLASMKDHFDQTGK
Q19TV7        --------------VFKNHGASAGASMTHPHSQMVGLPVIPPSVTTRLDSMKQYFNETGK
A0A2C9VAS1    --------------VFKNHGASAGASLSHSHSQIMALPVIPPSVSARLDGTKEYFDKTGK
A0A1R3JVQ6    --------------VFKNHGASAGASLSHSHSQILSLPIVPPSVSARLNSMKEYFNQTGK
A0A1Q3DBV3    --------------VFKNHGASAGASMSHSHSQILALPIIPPTVSDRIDGTKEYFDQTGK
A0A1J6JB59    -------------QVFKNHGASAGASMSHSHSQMIALPIVPPTVSARLDSMREYYKQTGK
A0A1R3GWA5    --------------VFKNHGASAGASLSHSHSQILSLPIVPPSVSARLNSMKEYFNQTGK
A0A4P1RJT6    --------------VFKNHGASAGASMSHSHSQILAVPIIPPNVSTRLESMKSYFDQNGK
A0A1U7YE15    -------------QVFKNHGASAGASMSHSHSQMIALPIVPPTVSARLDSMREYYKQTGK
A0A1U8HVG0    --------------VFKNHGASAGASMSHSHSQMLSLPIVPASVSTRLNSMKEHFNQTGK
A0A251VRJ8    -------------QVFKNHGASAGASMSHSHSQIIALPVIPSTASTRLNSMKEYHNQTGK
A0A2G2YK81    -------------QVFKNHGASAGASMSHSHSQMIALPIVPPTVSARLDSKKEYYKQTGK
A0A2G2VT83    -------------QVFKNHGASAGASMSHSHSQMIALPIVPPTVSARLDSKKEYYKQTGK
A0A2G9H502    --------------VFKNHGASAGASLSHSHSQIMALPVVPPTAAARIQSMKEYFGQTGK
A0A2I0L9H2    --------------VFKNHGASAGASLSHSHSQIIALPVVPPNVSTRLGSMKDHFSRTGK
A0A9R1WPN2    -------------QVFKNHGASAGASMSHSHSQILALPVIPTIASNRLNTMKEHHNQTGK
A0A2I4GSU5    --------------VFKNHGASAGASMSHSHSQIIALPVVPANVSARLDSMNGFFHETGK
```

A0A2K3PBG0	-ALSAPNNVGTLVGVFKNHGEKAGASMSHSHSQILALPIIPPSVSARLGNMKDYFYQTGK
A0A2P5EMX1	--------------VFKNHGASAGASMSHSHSQMLALPIVPPNVSARIEGMKEYFGQTEK
A0A2P5B5X8	--------------VFKNHGASAGASMSHSHSQMLALPIVPPNVSARIESMKEYFGQTEK
A0A2P6RHM5	--------------VFKNHGASAGASMSHSHSQMMALPIVPPAVAARLDSMKDYFNRTGK
A0A2R6R525	--------------VFKNHGASAGASMSHSHSQIMALPVVPPAVSARLDSMKEYFDQTGK
A0A2Z6PED2	--------------VFKNHGEKAGGSMSHSHSQILALPIIPPSVSARLGNMKDYFYQTGK
A0A103XDX8	-------------QVFKNHGASAGASMSHSHSQILALPVVPPTASTRLDSMKEYHNQTGR
A0A2U1PK25	-------------QVFKNHGASAGASLSHSHSQILALPVIPTTVSTRLDSMKEYHNQTGR
A0A328E5T3	-------------QVFKNHGASAGASMSHSHSQIIALPIVPPTVSARLSSMEEYFQQTGS
A0A2Z7DDR5	-------------QVFKNHGASAGASMSHPHSQIMALPVIPSTVSARLNSMKEYFVQTGN
A0A314Z4V0	--------------VFKNHGASAGASMSHSHSQIMALPVVPPAVATRLGSMKEYFDQTGK
A0A392NUD2	--
A0A445GIZ5	--------------VFKNHGASAGASMSHSHSQMMALPVIPPSVSARLTSMKDHFDQTGK
A0A498IJM2	--------------VFKNHGASAGASMSHSHSQIMALPIVPTAVAARLDSMKEYFDQTGN
A0A0S3TAB0	--------------VFKNHGASAGASMSHSHSQMMALPIIPPSVSARLASMKDHFDQTGK
A0A068V0Z0	-------------QVFKNHGASAGASLSHSHSQIIALPVVPTAASARIDCMKQHFEQTGK
A0A8X8WFE4	-------------QVFKNHGATAGASMSHSHSQILALPVIPPTASARIQSMKEHFEQTGE
A0A4V3WL65	--------------VFKNHGASAGASMTHSHSQMMGLPFVPPAVSARLDSMKENFDQTGK
A0A4U5PVU0	--------------VFKNHGVSAGASMSHSHSQILALPVIPSSVSARLEGMKEYFEKTGK
A0A540L1S0	--------------VFKNHGASAGASMSHSHSQIMALPIVPTAVAARLDSMKEYFDQTGN
A0A5A7U3W6	-------------QVFKNHGASAGASMTHSHSQMVGLPVIPPSVTTRLDSMKQYFNETGK
A0A5C7IC11	CGYKTVAHYVARWAVFKNHGASAGASMSHSHSQILALPVIPPTVSTRLCSTKEYFDQTGK
A0A5D2XCB5	--------------VFKNHGASAGASMSHSHSQMLSLPIVPASVSTRLNSMKEHFNQTGK
A0A5A7PC75	-------------QVFKNHGASAGASMSHSHSQIMGLPVIPPSASARIQSMREHFDETGK
A0A5B6VU70	--------------VFKNHGASAGASMSHSHSQMLSLPIVPPSVSTRLNSMKEHFNQTGK
A0A5J4ZKC7	--------------VFKNYGASAGASMSHSHSQIMALPVIPPTVSTRINSMKEYFDQTGK
A0A5N6P247	-------------QVFKNHGASAGASMSHSHSQILALPIIPSLASNRLSSMEEYHNQTGK
A0A5N5J241	--------------VFKNHGASAGASMSHSHSQLLALPIIPSAVSARFEGMNEYFKKTGK
A0A5N6RJP1	--------------VFKNYGASAGASMSHSHSQILALPVVPPTVSTRLDSAKEFFHETGK
A0A5E4FF82	--------------VFKNHGASAGASMSHSHSQIMALPVVPSAVATRLGSMKEYFDQTGK
A0A6A4LKW8	--------------VFKNHGASAGASMSHSHSQIIALPIVPPTVSARLHSMKEHFDQTGR

```
A0A6A4QFB2   --------------VFKNHGASAGASMSHSHSQILAVPIIPTNVSTRLESMKCYFDQKGK
A0A6A6M4N8   --------------VFKNHGASAGASLSHSHSQIIALPIVPPAVSARLDGTKEYFDKTGK
A0A6D2L947   --------------VFKNQGASAGASMSHSHSQIMALPVVPPTVSSRLDGTKDYFDETGK
A0A565CNH9   --------------VFKNHGASAGASMSHSHSQIMALPVVPPTVSSRLDGTKDYFDETGK
A0A6J1DP04   --------------VFKNHGASAGASMTHSHSQMVALPVIPPSVSTRIDSMKEYFNKTAK
A0A6I9TLA4   --------------VFKNHGASAGASMSHSHSQIVALPIVPPTASARIQSMKEDFAQTGK
A0A6J1IZ13   --------------VFKNHGASAGASMTHSHSQMMGLPVIPPSVSTRLDSMKQYFNQTGK
A0A6J0KBW1   --------------VFKNHGASAGASMSHSHSQIMALPVVPPTVSSRLDGTKDYFEETGK
A0A6J1A689   --------------VFKNHGASAGASLSHSHSQILSLPVVPPSVSARVGSMKEYFNQTGK
A0A6J5XVC3   --------------VFKNHGASAGASMSHSHSQIMALPVVPPAVATRLGSMKEYFEQTGK
A0A6P5WUV9   --------------VFKNHGASAGASLSHSHSQMLSLPIVPPSVSARLDSMKEYFNQTGK
A0A6P5SY58   --------------VFKNHGASAGASMSHSHSQIMALPVVPPAVATRLGSMKEFFDQTGK
A0A6P6SUB1   -------------QVFKNHGASAGASLSHSHSQIIALPVVPTAVSARIDCMKQYFEQTGK
A0A6P3Z169   --------------VFKNHGASAGASMSHSHSQILALPVAPPAVSTRLGSMKEYFDKTGK
A0A9C6T7J3   -------------LVFKNHGASAGASMTHSHSQMIALPVIPPNVSARLGNMKDYFEKTGK
A0A6A1W7E7   --------------VFKNHGASAGASMSHSHSQILALPVIPPDASVRLDSMKEFFHETGK
A0A7J6HQZ5   --------------VFKNHGASAGASMSHSHSQMLALPIVPPAVSTRVESMKEYFNQTGK
A0A7J9H1V9   --------------VFKNHGASAGASMSHSHSQMLSLPIVPPSVSTRLNSMKEHFNQTGK
A0A7J8T974   --------------VFKNHGASAGASMSHSHSQMLSLPIVPPSVSTRLNSMKEHFIQTGK
A0A7J7DS99   --------------VFKNHGASAGASLSHSHSQILALPVVPPNVSTRLDCMKEYFARVGR
A0A7J7GKE2   --------------VFKNHGASAGASMTHSHSQMMGLPFVPPAVSARLDSMKENFDQTGK
A0A7J8M9P9   --------------VFKNHGASAGASMSHSHSQMLSLPIVPPSVSTRLNSMKEHFNQTGK
A0A7J8UUY6   --------------VFKNHGASAGASMSHSHSQMLSLPIVPPSVSTRLNSMKEHFIQTGK
A0A7J8ZYH9   --------------VFKNHGASAGASMSHSHSQMLSLPIVPPSVSTRLNSMKEHFNQTGK
A0A7J9JF36   --------------VFKNHGASAGASMSHSHSQMLSLPIVPPSVSTRLNSMKEHFNQTGK
A0A7J9LPG1   --------------VFKNHGASAGASMSHSHSQMLSLPIVPPSVSTRLNSMKEHFNQTGK
A0A7J8XJ49   --------------VFKNHGASAGASMSHSHSQMLSLPIVPPSVSTRLNSMKEHFNQTGK
A0A7N2M4Q6   --------------VFKNHGASAGASLSHSHSQIIGLPIVPPNVSARVDSMKQYFNETGK
A0A7N0VFH9   -------------QVFKNHGASAGASMSHSHSQIMALPVVPPLVVSRVASMKEFQAKFGA
A0A8S0UB89   -----------TCEVFKNHGSSAGASMSHSHSQIMALPVVPSAVSSRIESMKEFFEQTGK
A0A484KNA0   -------------QVFKNHGASAGASMSHSHSQIIALPIVPPTVSARLSSMEEYFQQTGS
```

A0A803MCA9 -------------QVFKNHGASAGASMSHSHSQIIALPIVPPSVSTRLAAMKEHFEQTGN
A0A834H459 --------------VFKNHGASAGASMSHSHSQIMALPIVPPTTSARLHSMKEHFDQTGR
A0A834VYP3 --------------VFKNHGASAGASMSHSHSQILALPIIPPNVSARLGSMKDYFEQKGK
A0A830D8Y2 -------------QVFKNHGASAGASMSHSHSQIMGLPIIPPLASARHQSMKEYFDQTGK
A0A835IYB6 --------------VFKNHGASAGASMSHSHSQILALPIIPSAVSDRFEGMNEYFKKTGK
A0A8B8LU99 --------------VFKNHGASAGASMSHSHSQMMALPIIPPNVSARLGSVKDYFDQTGK
A0A8B8N3B5 -------------QVFKNHGAPAGASLSHSHSQIIALPIVPSNVSAQIECLKEHFDRTGK
A0A8J5YDI6 --------------VFKNHGASAGASMSHSHSQMLSLPIVPASVTTRLNSMKEHFHQTGK
A0A8J4QZ02 --------------VFKNHGASAGASLSHSHSQIIGLPVVPPNVSARVDSMKQFFNETGK
A0A8K0GXL3 --------------VFKNHGASAGASLSHSHSQILALPVVPPAVSARLDGTKEYFDKTGK
A0A8T2WL49 --------------VFKNHGASAGASMSHSHSQILALPIIPSSVSARFEGMNEYFEKTGK
A0A8T1PAW8 --------------VFKNHGASAGASMSHSHSQILALPVVPSDVSARLDSMNEFFRETGK
A0A978UDM2 --------------VFKNHGASAGASMSHSHSQILALPVAPPAVSTRLGSMKEYFDKTGK
A0A9J5X336 -------------QVFKNHGASAGASMSHSHSQMIALPIVPPTVSARLDSMMEYYKQTGK
A0A9D3VIS5 --------------VFKNHGASAGASMSHSHSQMLSLPIVPPSVSTQLNSMKEHFNQTGK
A0A8X7S5T2 --------------VFKNHGASAGASMSHSHSQIMALPVVPPTVSSRLDGTKDYFEETGK
A0A8X7ZIJ2 --------------VFKNHGASAGASMSHSHSQILALPVIPSSVSARLEGMKEYFEKTGK
A0A9D5BHE4 --------------VFKNHGAAAGASMTHSHSQMIAMPVIPPTVSARLGSMKDFYDQTGK
A0A9Q0J1K7 --------------VFKNHGASAGASMSHSHSQIMALPIIPSSVSARLDGMKEHFDNTGK
A0A9Q0T1I6 --------------VFKNHGASAGASMSHSHSQLLALPIIPSAVSARFEGMNEYFKKTGK
A0A9Q0V9I9 --------------VFKNHGASAGASMSHSHSQLLALPIIPSAVSARFEGMNEYFKKTGK
A0A9Q0W9M7 --------------VFKNHGASAGASMSHSHSQLLALPIIPSAVSARFEGMNEYFKKTGK
A0A9P1EFQ7 -------------QVFKNHGASAGASMSHSHSQIIALPIIPPTVSARLGSMKEYFQQRGT
A0A9Q1RSR0 -------------QVFKNHGASAGASMSHSHTQIIALPIVPPTVSARLDSMMEYYKQTGK
A0A9Q1L1H9 -------------QVFKNHGASAGASMSHSHSQIIALPIIPPSISTRLTAMKEHFDQTGK
A0A9R0K9C0 -------------QVFKNHGASAGASMSHSHSQIIALPIVPPSISTRLAAMKDHFEQTGK
A0A9W7HNU1 --------------VFKNHGASAGASMSHSHSQIIALPIVPPNVSARLDGMKEYFNQTGK
A0AA38ZUR1 --------------VFKNHGASAGASMSHSHSQIIALPIVPPTVSSRLDCMKGYFEQTGK
A0AA39SLE4 --------------VFKNHGASAGASMSHSHSQILALPVIPPTVSTRLCSTKEYFDQTGK
A0AA38WNJ4 -------------QVFKNHGASAGASMSHSHSQILALPVVPTTASARLDSMKEYHNQTGR
A0AA35Y5R7 -------------QVFKNHGASAGASMSHSHSQILALPVIPTIASNRLNTMKEHHNQTGK

(C) d3sjld__{A}_7_18_2.914e-08 mean AL2CO score = –1.01(±0.25)

```
d3sjld_        ------------------------------------------------qetqgqaaaraaaad
A1BB97         IGLGLGCSALA--LAASAQDAPEA------------------ETQAQETQGQAAARAAAAD
A9HIB8         AAPLSVAALLGAAILQ-----------------------------------PLVPPSGARAA
B2JN92         IAATL------------------------------------------------SFAAACIGSA
A0A9J9HDB1     -LR------------FA------------------------------------ALCLATAAPA
Q50420         AGQLAATALLA-ATCSAF-----------------------------AADAPTTVPSSLGAI
A1K7S2         RTCLAA--VL-IALAG--------------------------------------------GSA
C6WX30         AGKLAIAALLV-AAQPAF-----------------------------AED-QKAVPKQLAAV
C6XQB5         SCALGA-----LIFLA-----------------------------------SPVGFAQE---
H5WRS2         ----A------AALACALA------------------------------STAVAAPQAPKAQ
C0N7A3         VGGIAAAALMT-AAYPAL--------------------------------AD----------
B8KXR2         -QR----------LAV------------------------------------LMALLMASLA
A0Z932         -SL----------FVALL--------------------------------TAIGMLVSAEEI
F1Z7Y8         SAASAMGLALAATIGA-----------------------------------ICLPAAPALAG
I0JZ89         ----------------------------------------------------------------
U2E0W8         AGG-VA--A--------------------------------------------MAALAWSASA
K6Z5F1         ----------------------------------------------------------------
K0CBH2         AGLLA---L--------------------------------------------GSVL-IPPSL
K6Z5K0         AASLLL--LG----CTATPVAT--------------DGV-KSLAQLETVDSNTVNIKPDPS
K2IVH7         FGLLLF------TSLV-----------------------------------SRAALAQDEPS
H6RJW5         CGLLL--------AVT-----------------------------------AGCARPVDDPG
A0A0H3KP02     LAATL------------------------------------------------ALAATSAGGA
G4RF01         L--ALA-----VGTAL-----------------------------------TLPAFAQENAD
Q49124         LGAAALAALIA--AG----------------------------SAAAAESHGVATAKAAAAD
Q2G7P8         SVS-----G-----AAL-----------------------------------AATPAAKAPY
```

```
F6IE79        GLL-----S-----LTP------------------------------SPNASAQDPGKF
K9DEQ3        FATAAMGFVLCAA------------------------------------ITAQAAPADT
M4RZL2        AGIAWAG----IASAA--------------------------------DTPPPAPASPP
M7PU72        AGKIAIATLLA-ASQPVL-----------------------------AGGAVPSQLGSE
N1MK08        FAAAAWGWALLVS------------------------------------PAQAAPA-DT
S5XJB0        LCAGLTCSVLA--LGTAYAETAAA---------------------PQTEGQKQAAEGAAK
S6AG49        AQTTLA--L----------------------------------------GVALAGLGNA
T0K0G4        ---MGCALLVTG-----------------------------------------TTLWA
U1ZL56        -------------MLGL----------------------------------MVLAGGAQ
F1YVG1        S--LLRALL--GSAAI---------------------------------LTMGGTAAKAA
V5SBY2        IGGVATLGLLA--GGAAYAQEGPR-------------------------GSELPKQLGQE
W0AE12        MLSASVGALLAAG------------------------------------MAHAAPATDP
W0PE43        ALITAFTSLAL-A---GA--------------------------ATAAEPKIPTQLGKE
A4A7G1        LAAVLL-------CVT--------------------------------QGLAAQPYG--
A0A023D170    N--FV--VPLLTAVLP---------------------------------CLAGLATPARA
W8X1W1        -IPTGA--GLLSAALLL----------------------------------GSAVGVQA
A0A086P9C6    -GC-----------IL---------------------------------SLCFATVIPA
A0A059DMU0    ALSLSA------SWAN--------------------------------AQEAAIPD---
A0A024HGH9    ARTMLA--L----------------------------------------GVSLAAAGGA
A0A069PTC1    MAAVL--------------------------------------------SGVLACQIQV
A0A074TIK4    PVLAAL-----LLCAA---------------------------------APGAFA--Q--
A0A8A8D8M4    LAATL--------------------------------------------ACAAAWAGSA
A0A085FEK6    AASALC-----CATML--------------------------------TFPLMAQEA--
A0A086Y511    ATICAA-----CAVLA--------------------------------GQAAIAQEA--
A0A0B5DX78    AAAFLA-----LSAIC--------------------------------G-QAFAQDE--
A0A0C4YG08    LSVLAL--PA-AAANA-------------------------------------SDAAS
A0A0D6JKV1    LGGVASLSLFV--SGAAYAQQSPS------------------------GAEVPKHLDEK
A0A0J1HFZ5    LGALQL--AA----CSSNQEVS------------AGSDESSATQQATHQVTQPVTPNSS
A0A0N0JJK2    LATATLAVALAGGLPGALA-----------------------------PSAMAAPVAAP
A0A0N1C876    SAS-----G-----TAL---------------------------------AATPAAETRF
A0A0P6WHV1    REVFA----------TLL------------------------------LASAAVLPTVAT
```

A0A0Q7E0E9 LSVCLA-----CALAA---------------------------------AAPTLAQEA--

A0A0Q7D1N1 ML--SAPCAAQTVA

A0A0Q6EGD5 VLLALA-----FGTAA---------------------------------FAPAHAQEE--

A0A0Q2QX28 LGALFI-----SGLIF---------------------------------LPTTNAATQ--

A0A0Q8R3S7 -TA-----------LA----------------------------------ALCLAAAAPA

A0A0Q6BI19 LLFAIA-----FSSTA---------------------------------LGSVQAADE--

A0A0J7XRT6 FAAALCGSLLASA-------------------------------------ALAQAAPADT

A0A0W7Z1A5 -----------MAFFA---------------------------------STASF-ASE--

A2WG71 LTATL---ALAATSAGGA

A0A158IF31 MAAVM---SGVVAWHLA-

A0A0U5EZH8 G--TAALVLGMMALGA---------------------------------FSVTSVKAWAD

A0A102D9N9 -AAL----HGAAMALA----------------------------------HPAAADTPTR

A0A124JU51 ALG-----V-----AAL-------------------------------LSAPAAAAENRF

A0A0N9UJL1 A--------------ML-------------------------------LMSASAAGAQNF

A0A125NTV3 LGGVASLVLIG-AAGVANAQQGPS-------------------------GAEVPKQLGAQ

A0A102DDU6 ALA-----V-----ASA-------------------------------QPA-L-AQAASV

A0A0S2KEG8 LAVMAS-------LAL---------------------------------SGAANAQWD--

A0A133XFV5 KLIALL--VL-AGLASG-----------------------------------MAIGEPRR

A0A149PR24 WALLM---LSSVAMATQV

A0A150L8H0 L--N------

A0A196MKB1 FAATLSAMLLGSA-------------------------------------AVAGAAPADT

A0A1B3MRN9 FAAAALGWTLFAT-------------------------------------AAWAAPAAET

A0A1E3GU71 AASMAMTAMFV-GGTQAV------------------------------IAGGVPQQLGSE

A0A1E8CNJ1 --

A0A1E7Z8Z0 PAL---LMACV-GLPA

A0A1D9MCS4 LA---------LPLGL---------------------------------ALLA-----PA

A0A1D9HBT7 --ATLA--AL-AAVCG---------------------------------------VPAAC

A0A1S2CE83 ACCLV---V---ASGL-IAASA

A0A1I3WIR9 TAAFVA-----LVALT---------------------------------G-PAFAQDA--

A0A1M4MXS9 TAMALA-----LMALA---------------------------------A-PAVAQEA--

A0A1Q8IW76 WALLM---AGSAAISAQV

```
A0A1N7RY02    WALLM---------------------------------------------VSGAAISAHV
A0A1U9KH93    A--FLRSLL--GGVV----------------------------------LIAATASARAD
A0A1V0GYS5    MASGL-----A--LGAAAAQD------------------------AQTDGQRAAAEAARL
A0A1Y6BCQ1    MGRVA----------TLLL----------------------------VAAVAAVGGAQAA
A0A1X7J6I5    LATGA-----A--F----AQ-------------------DSSPDETMTEGQRAAAEAARQ
A0A252B2Q1    S--WLGLLL--GSAAF---------------------------------FSA--ATVQAA
A0A246K5C7    ------------------------------------------------MASASVSAAADY
A0A239KUF4    -RHL----LCGLSLCA----------------------------------G-----AASA
A0A1G8ANR3    VCLLVC-----SAFSQ---------------------------------APVALAETQ--
A0A1H7BPS8    IAATL---------------------------------------------SFAAACIGSA
A0A1H8IVS7    IGSGL-----A--LGPSWAQTAEEPAEESQPAEAEAGEAAQTPAEEMTEGQRAAAEAAEK
A0A1H9RWD6    FATALSALLLGSA-------------------------------------AIAHADLADT
A0A1I0H738    LLTSFS-----ALVAS---------------------------------GTFVAA--Q--
A0A1I2MJ34    VAALALSGPALYGI-----------------------------------AAFAPAANAKD
A0A1I7LZ48    RRGV----AAGAAMCA------------------------------------LGAAGQAM
A0A1I3FQ37    L--------------V----------------------------------ALGVSAASVA
A0A1I3TD09    TAAFVA-----LAALT---------------------------------G-PAFAQDA--
A0A257LQY0    -RWAAW--CL-AATL-------------------------------------GCGHMGAA
A0A255YLN0    -RPL----------TL----------------------------------VAALLLAGSA
A0A255Z6A5    -SLI----------AL----------------------------------CAAIALVAPA
A0A291GDG1    TAAFVV-----LAALT---------------------------------G-PAFAQDA--
A0A2A4I8K1    ----IAATALLA---------------------------------------GATAAPAQS
A0A2A4B323    IAALLSA----AGV-----------------------------------QA--QAQTPPR
A0A2A8HV00    TAALWLA----LGI-----------------------------------AAVPGTATARE
A0A2M6UYR1    L-GAV------WAAAGLLC--------------------------------HAAPAARVA
A0A2N3LZ45    LTHVAF-----GAFLV---------------------------------AAPA-----AL
A0A2N5EMF6    LTLFTT-----LGILS---------------------------------FTTSQAAEP--
A0A2N5Y225    LGA----------IVAVG------------------------------H---AFAEDREV
A0A2I8DPH5    -TRAAR--AGGWALCAA-----------------------------------LAAAAGAS
A0A2U3HZK8    MGALF---------------------------------------------YGLLACQLHA
A0A2S5KIC4    SASLLA--L-------------------------------------------VL-AGSQA
```

A0A2S8RZ29 LILSLS-----VLAAS--------------------------------GAQVLA--Q--
A0A1H0H1E2 ANTTLA--L--GVALASVPSA
A0A1H2EX98 ARTTLA--L--GMALAG--AA
A0A1H2F3S3 -KRL----LRGFTLCI---------------------------------ALLAWPVTGV
A0A1H1YFW2 ARTALA--F--TVALAS--VA
A0A2T5FZ30 LAAVISP----VTL----------------------------------QA--ES-----
A0A323UUH5 KNCVAA--LL-IALGG--ATA
A0A2Z2NRF1 PRRLLA--LAIFGICAAGSANA----------------------------QTTKTDAEL
A0A315ABB6 M-GVL------SALAGSLI---------------------------FAASAHAATAQSV
A0A2Z4UQF9 -AAL----LAGFLFT----------------------------------GVSTLSTSAA
A0A369T7V0 -ASAMA--AALAGVLF---------------------------------AGTAAAQVPE
A0A3D8JVF2 WALAM--ACGATIAAHA
A0A398BT79 LLLSLS-----ILAVT--------------------------------GSQVLA--Q--
A0A1W7M7Y5 -SLL----ALGASQAM---------------------------------AAPPKPAPPK
A0A3S4CAP5 CSALLA-----AQLAA--------------------------------PVSVLAQDD--
A0A3N6NJG1 -RLIS----G-VAVLATVL--------------------------------LSAQAPVA
A0A3N7CFA9 L-M--------------SL---------------------------SVAAATAATPAAL
A0A3S3TT68 RLAPL-------AL----------------------------------AVASGTARAAP
A0A437J7B1 PVRALALALTLAG------------------------------------SSLSAAPADT
A0A410GFE7 -LP-----GL--AACAL----------------------------------MALVSPAG
A0A418ZRX7 ---MTEGQRAAAEAAAQ
A0A423PIM0 ----M------LLGFT--------------------------------GAAALAADK--
A0A401J738 FAAAAWGWALFAG------------------------------------PTQAAPA-DT
A0A4Q7LIS5 AIGLLS-LLGVTGLATS-----------------------------FGALAQAGKARAV
A0A4R0XD57 IAATL--SFAAACIGSA
A0A4R6RC52 RVAVLA-----AAMAV--------------------------------AVPALAEDA--
A0A4V3BNW1 SFRVKS--LL-FGLCA--------------------------------------GFAMQ
A0A4R2KBX4 LAAAMA-----VPLTL---------------------------------AGG-----PA
A0A4V2SDJ7 PAAV----L--A-GALVAGSA
A0A4Y8UXA1 --------------MS--------------------------------SGAANAQWD--
A0A4P7XM83 ITLGAV-----LGVLA--------------------------------VGGAQADDD--

```
A0A4Y8UN28      SAAALL--LGSYG-CAPEAEQA-------------DTS------HADHVAPAVEIVADPS
A0A562NTZ2      LGLGMTCSSLA--LGTATAQEATAD-------DAALTSAGVTRNDGESQGAAGARTAAAD
A0A558NPA9      --I---------ATLL---------------------------------LSAVMPAAQ--
A0A4Y6V628      ------SITLFVALAV---------------------------------FST--PALAEP
A0A4Y3TVW3      G--LSSLLA--GGIA----------------------------------LLALPAFAGAA
A0A523Q339      R-RSL------SGLC--VL----------------------------LLSTAALAAPSTQ
A0A501PGL6      IGA-----L-----------------------------------------CAACVWPAVG
A0A501WV29      FLVALS-----VLAAA---------------------------------GTPALA-----
A0A511X7F5      V--FATCLG-----AA---------------------------------LLSSTTHARAE
A0A511XFQ7      A--TAVCATGLLA-------------------------------------GSVSERALAS
A0A1I4E4L0      ALLGLS-----FISLL---------------------------------ASAAFAQDE--
A0A5D0X323      -ASALLA----LALPG---------------------------------AA--IAAQGRL
A0A6C2CLF7      --------------MS-----------------------------------------AGA
A0A5E4RPP3      MALTLW-------IMT----------------------------------AQAQAQTKTL
A0A6I5YKB7      FAAAFSALLLGSA-------------------------------------TLAHADLADT
A0A5S9QH23      --LST------LLSLL---------------------------------CLIQHVQLH--
A0A6N6VMG0      -MRV----WSSIAIA-----------------------------------ALMLVMAPAT
A0A6C0U555      MAA----------LCGLG------------------------------LGFAAVAAAGDQ
A0A6L6JDP0      TVLPIF-----LASLA---------------------------------L-PAVAQET--
A0A7C9TIH4      RRLARL--PQALGLL-------------------------------------LAAGLGLA
A0A6J5FID0      AAAVL---------------------------------------------AFA----CAA
A0A6S6ZV48      -SRALR--AGRWALCAA-----------------------------------LASATGLA
A0A6N0WYA5      ALG-----L-----AVL-------------------------------PAH-AATPAESY
A0A7L5RWA2      LTLSVA-----LGMLA---------------------------------TVPAFAEEP--
A0A7Y9QWY9      LGAVLL-A------CSS------------------------------ATWAANKASAKPV
A0A7X0JRG8      ISW-----------------------------------------------PLMLA-ALVV
A0A7Y7IV73      AAALAVSGLLGA--------------------------------------AAPHDAARAA
A0A7W4KEC0      A--MA--AMLA--------------------------------------APAG--AALAA
A0A7W6CEB8      RAAAV-----LIGT-----------------------------------VLASGAQA---
A0A840SJ55      IALGM-----A--FGPASAQEQQTPEQPAQEQQ------AQGEQTTQTEGEREAAEAAQA
A0A7W6DDQ9      VAALAV------GV-----------------------------------AAIGFPAQATD
```

A0A7W4J5F5 A--FS--AMLM------------------------------------VSAA--TAHAD
A0A7W5ZZB3 -GLL----V--SSADA--------------------------------RPAKKAAPPK
A0A7W7B107 T-------------LL-----------------------------FAASDAQAQQAF
A0A848G7N7 ---
A0A853FCE1 --------M--PASAQ---------------------------------EPKTPQVQ
A0A7U7EL64 MQAVMA--L---------------------------------------GVSLA--GHA
A0A850PC06 ----ARCAVLPALTCV-------------------------------SVAHAGPASAS
A0A7R7HAE8 IFLGLS-----FATLM-------------------------------TAGPVLAQE--
A0A967B6I1 A--MLCSLA--SGLA--------------------------------LLAGATSGRAE
A0A936Z330 -GLSL---LIAAGAA
A0A931HFP9 ----MASTLLLGSV---------------------------------AALTVPAQAQD
A0A917GPW5 LALVTS-------LVA-------------------------------CSAVNAQWD--
A0A919AKV3 VAT-----A---------------------------------------CFFCT-GALA
A0A974Y937 ---
A0A939DFR0 ---------------MS----------------------------L-APTAAGAEDR
A0A916IQB5 RRLIPL--LL-AATLG-------------------------------------AGAAV
A0A8B6X9Q5 ARLVRF--LL-AAGLA-------------------------------------GAALS
A0A940SI03 IAATL--SLIAACIGSA
A0A9N8RZ47 LAFAL--AGTAMMSLHA
A0A977SHT6 AASTLC-----CATML-------------------------------TFPVMAQEA--
A0A9J6RMI2 -------------------MA---------------------------ASSYAAKAE
A0A9Q3YNS7 CTGLL---L---------------------------------------AAAA-VSATA
A0A9X1DC07 AAA-----RVLSSL---------------------------------TILALMTASSA
A0A9X3U0E8 --------------MI------------------------------SLCGTAAGAADF
A0A9W6JCY9 LGSAAFAALLA--AG-------------------------PAAAAETHGQAGAKAAAAH
A0A9X3H996 SGT-----I-----CGS-------------------------------ALAAPNTGPY
A0AA41Z7Q8 ALG-----V-----AAL-----------------------------PAL-AGAAPGTG

d3sjld_ l-------aagqdd--ep------rile--apa-pdarrvyvndpah-faavtq-qfvid
A1BB97 L-------AAGQDD--EP------RILE--APA-PDARRVYVNDPAH-FAAVTQ-QFVID

```
A9HIB8        E-------PILQTE--ES------DIVT--LPP-GGPHRVLVQDGVYRHSKDGR-VYVVD
B2JN92        H-------AIEKPE--EL------TVQK--LPA-WHSHEVFVVDISMPSMTDAR-IYLYD
A0A9J9HDB1    L-------AQLQPE--EL------TKES--LAETMQPHWVWLNDISFDRMLDGR-AYLVD
Q50420        G-------TTPTLD--NI------TMEP--GLP-SDAKRVYVLDPGH-FHVTTT-VYTID
A1K7S2        Q-------AELQVE--IP------KTVD---LPPPNPYRIYLSDISIGHIVDGR-LHVID
C6WX30        G-------SL-KID--NL------SVAK--APP-VDAKRFYVIDPGH-FNMTSQ-TYVMG
C6XQB5        ---------PIAPE--EL------TVEK----EIPDGENVYVLSSSWA--GASS-VLVLS
H5WRS2        P-------PELPAE--TL------QRGG--TLVGATTERIYVGDVAIAHIADGR-LRVFD
C0N7A3        ----------IAIE--EM------TLEV--APA-SDSKRVYVTDPGH-FQMTSQ-VFTID
B8KXR2        H-------AGVKPE--TI------SVAK--LG-EPTPTWVFIKA------YMGG-TNLFD
A0Z932        R-------PPLAVE-PTG------IIET--LPGRYPADWFLVHDAAFFHMSDGK-VVVLD
F1Z7Y8        T-------ASVEPE--DS------DVAP--LPM-PKPSWFYIQRGF----VQAG-TGVYD
I0JZ89        ------------------------------------------MDQLF-TVANRK-AFLID
U2E0W8        W-------SQLPPD-EIG------T-VT--L-DKPGPHRAYIIDTQFDNATTGK-VVVVD
K6Z5F1        ------------------------------------------------------------
K0CBH2        Y-------AALPTE-TVG------Q-AE--LPFPPSPHRSYLIDFEFDNMVATR-VVVVD
K6Z5K0        L-------PPLPIE-KTG------KIEV--LPAKYPESWMFVDEVSFQSQFGGK-MIILD
K2IVH7        -H------PPFEFE--TP------TALA----AIAPGPNVFVNSQGWD--KASA-IKVYG
H6RJW5        APLDGLRACPAAGS--VIGVFVGAGPTA----DAGDGPTVQGVD-TWTLSVGGD-VRRL-
A0A0H3KP02    H-------AAEKPE--EL------VVQK--MPP-WHPHEIYIVDISMPSMTDGR-IYVYD
G4RF01        AP------EPIAPE--TL------TVKE----TIDPGPNIFSNAQSWD--GASQ-IQVWS
Q49124        L-------AAGKAD--DP------VVLK--AAP-INARRVFVYDPKH-FAAISQ-FYMID
Q2G7P8        P-------KPLPEE-PIP------ASAK--LPSQWPASVVLIHDFHFSSIVDGR-VMVVD
F6IE79        A-------QPLPVE-EVP------SVQS--LPPLYPKDWVLIQDFHFNALIDGR-LAIVD
K9DEQ3        T-------AALEPE--TS------DVVT--MEP-PKPNWFFVDGGW----DMPG-TSIFD
M4RZL2        P-------PILEAE--TS------GTLT--LGP-AGAHRLYIAA-------GEG-FTIID
M7PU72        A-------TVQLMD--EP------TILT--APP-SDAKRVYVLDPGA-FHMTSQ-IFTID
N1MK08        G-------TVLEME--TS------DVLT--ITP-PKPTWFFVDGGW----DMPG-TSIFD
S5XJB0        L-------AKGEDD--EP------HIGT--MPK-ADAHRVYVNDPAH-FAAITQ-QFIID
S6AG49        A-------AELPADAGIG------Q-TT--LPFPPEPHRAYIVDVEFNNFVAGR-VTVVD
T0K0G4        Q-------SAPEPE--TS------DVVK--MEP-PKPDWFFVDGGW----NMPG-TSIFD
```

```
U1ZL56         A-------QDKPRE--VL------TGGH--SVSAPQENRIYVMDSVFMHLTESR-VHVYD
F1YVG1         E-------PVLQTE--QS------DVIT--LPA-YSPHTILVEDAVYTHNKDGR-VYVVD
V5SBY2         A-------TSDLED--NP------VILS--APE-PDARRIYVTDPAH-FAVTTQ-HFTVD
W0AE12         N-------PVGESE--TS------DVMP--LAA-PSPAWFYVDGGW----DMDG-TSIFD
W0PE43         A-------VSTLQE--EP------KILS--APP-SNAKRVYVTDPGH-FNVTSQ-VFSID
A4A7G1         ---------SIVGD--AL------KLG--------------TPNDHWFSARGKQIAYLID
A0A023D170     A-------DTLEPE--QE------YTEK--LPPTMGEHWALV-T------TNGS-FTLYD
W8X1W1         A-------PPLPVE--QL------TGGH--SVKVDPAERIYVMDAVFFHLTDSR-LNMYD
A0A086P9C6     S-------AQLAPE--EL------TQET--LADTMQPHWVWLNDVSFDRMLDGR-AYLID
A0A059DMU0     ---------PIEPE--EL------ITET----EIAPGPNVFVVSPSWI--GAGA-ISILS
A0A024HGH9     Y-------ADLPAE-TIG------Q-ET--LPFPPSPHRAYIIDVEFESFVIGR-VTVVD
A0A069PTC1     H-------AAEKPE--EL------TVAK--VPP-YSPHQVFVPDISFSSMTDAR-VHVFD
A0A074TIK4     -D------TSIKPE--KL------TIRP----HISDGPHLFVLDFGMN--GSSP-IYVYD
A0A8A8D8M4     N-------ATEKPE--EL------VVQK--MPP-WHPHEVYIVDISMPSMTDGR-IYVYD
A0A085FEK6     ---------EFVPE--TF------SVKE----AIEPGPNIFVGKQEWG--GASS-IEVYS
A0A086Y511     ----------IAPE--KL------TVEE----RIAPGANVLTLDQSWS--GQSR-INVLS
A0A0B5DX78     -T------APIEPE--TL------TVKE----RIDEGPNLFVLDQKWA--GPSS-VNVLG
A0A0C4YG08     T-------AELQPE--TL------SVQP---LAPADDGRLYISDVAFKHMVDGR-LHVVD
A0A0D6JKV1     A-------TSQLED--DP------QILS--AST-PDSRRIYITDPAH-FAVATQ-TFTID
A0A0J1HFZ5     L-------PSLPIE-TVG------KVER--LPKVFPESWIYVDETSFSSMFGGK-MILMD
A0A0N0JJK2     I-------ASVEPE--QS------DVAQ--LTP-PKTSWFFINGGF----TSNS-SVIYD
A0A0N1C876     P-------KPLAEE-PIP------IIET--LPAQWPASWVMVHDLHFASILDGR-VMIID
A0A0P6WHV1     A-------EDLRPE--KV------TVAR----LDAATPRIYAVDIAINHIVDGR-AYVLN
A0A0Q7E0E9     ---------EFKPE--SV------GVKA----AIDPGPNVFVNKQEWG--GAGS-INVYS
A0A0Q7D1N1     S-------QPLPEE-AIP------SVSE--LPAAYPPSWILVHDLHGASIVDGR-LVIAD
A0A0Q6EGD5     ----------FEPE--QL------SVEA----KVRPGPNIFVLDQSWK--GSSR-LNVLS
A0A0Q2QX28     ----------FKPE--HV------SVEK----EIKPGPNVFVIDQSWA--GSST-ITVLG
A0A0Q8R3S7     W-------AQLQPE--EL------KLES--LAETMQPHWVWLNDISFDRMLDGR-AYLVD
A0A0Q6BI19     ----------FEPE--QL------TVEA----KVRPGPNIFVLDQSWR--GSSR-LNVLS
A0A0J7XRT6     K-------ASVEPE--ES------DTLT--IDP-PKPTWFFVDGGW----DMPG-TSIFD
A0A0W7Z1A5     -Q------KPFEPE--VL------TVEQ----RIAPGPNILVLDQSWK--GATR-INVMN
```

```
A2WG71        H-------AAEKPE--EL------VVQK--MPP-WHPHEVYVVDISMPSMTDGR-IYVYD
A0A158IF31    S-------AAEKPE--EL------TVAK--VPP-YSPHQIYVPDISFASMTDAR-VHVFD
A0A0U5EZH8    E-------PVVQEE--ES------DIIK--LSP-VTPHRILVQDTVGTHAKDGR-VYVVD
A0A102D9N9    P-------PVLQPE--TL------SVVE--LKDRMQPHWVWINDISFNHMSDGR-AYLVD
A0A124JU51    P-------QPLAEE-SLP------SVAK--LPAEWPLSWVTIHDFHFNAIIDGR-IAVVD
A0A0N9UJL1    P-------QPLPEE-PVP------TVSE--LPERYPDSWVFVHDLHFNSLPDGR-AAIVD
A0A125NTV3    A-------TSELED--TP------VILS--APA-PDARRLYITDPAH-FATTTR-TFTVD
A0A102DDU6    S-------AALPVE-PIP------TVTA--LPAQWPRSWVLINDFNFNSIVDGR-VVVVD
A0A0S2KEG8    ---------SVVGD--VG------ILE--------------EIDHHWVTANRGNTIYMVN
A0A133XFV5    P-------PPLPEE--EI------KVER---LAPADGNRLYVVDPAFGHMVDGR-THVLD
A0A149PR24    L-------ATEKAE--DL------TIAK--MPA-YSPHQLYVMDVNFSSMTDAR-TYVID
A0A150L8H0    ----------INND--ELA---VTNFSD----KLAEGSKVVIVD----------------
A0A196MKB1    S-------VALEPE--ES------DVLT--IDP-PKPTWFFVDGGW----DMPG-TSIFD
A0A1B3MRN9    D-------AVPEAE--TS------DVMT--IPP-PKATWFFVDGGW----DMAG-TSIFD
A0A1E3GU71    A-------TLDLMD--TP------VILD--APP-SDAKRVYVLDAGA-FHMTST-YYTID
A0A1E8CNJ1    -------------------------ME--------------GFDQHWVTVRRGDTVFLVD
A0A1E7Z8Z0    I-------AELEPE-PIP------SVAT--LPSVYPDSWIFAHDVNFNAMISGK-VVIID
A0A1D9MCS4    LA------EEFTPE--VV------TTKE----TIDPGANVFVSQQQWI--GAGS-IAVFD
A0A1D9HBT7    A-------EDLPPE--TL------TVAQ---LPPADADRLYISDVAFRHMVDGR-LHVVD
A0A1S2CE83    N-------ARLPNE-TVG------S-TT--LPL-PDDHRSYMVDFEFNNMVSTR-VVVID
A0A1I3WIR9    ----------IEPE--TL------TVKE----TIDEGPNLFVLSQEWK--GPSS-INVLG
A0A1M4MXS9    ----------IEPE--TL------TVKE----TIDPGHNVFVMDQAWA--GPSS-VYVVG
A0A1Q8IW76    L-------AVEKAE--DL------TIAK--MPP-FSPHHLYVVDVNFSSMTDAR-TYVLD
A0A1N7RY02    L-------AVEKAE--DL------TIAK--MPA-YSPHQIYVLDVNVSSMTDAR-TYVLD
A0A1U9KH93    E-------PIPQTE--QS------DIVT--LAP-WSPHTILVVDAVYQHGKDGR-AYVVD
A0A1V0GYS5    L-------AEGQAD--EP------VVLN--APK-PDARRVYVQDPAH-FAAITQ-QFAID
A0A1Y6BCQ1    A-------QELKPE--EV------TVAR----IPADTPKVYLADIAINHIVDGR-LYILD
A0A1X7J6I5    L-------AAGEAD--EP------VILN--APK-PDARRVYVQDPAH-FAAVTQ-QFVID
A0A252B2Q1    E-------PVLQTE--QS------DVVT--LKP-YSPHTILVEDAVYTHNKDGR-VDVVD
A0A246K5C7    P-------QPLAEE-PVP------SVAE--LPADYPDSWVLVHDLHFNSLPDGR-AAVVD
A0A239KUF4    F-------AAFVPE--QA------RVEV--LGEHRDQHWFWIWGSNAPNMVDGR-AYLFD
```

```
A0A1G8ANR3      ----------FVPD--HV------SVEK----EIKPGPNVFVMDQSWS--GTSM-VTVFG
A0A1H7BPS8      H-------AIEKPE--EL------TVQK--LPP-WHPHEVFVVDISMPSMTDAR-VYLYD
A0A1H8IVS7      L-------AAGEDD--EP------VILD--APA-PDARRVYIQDPAH-FAAVTQ-QYVVD
A0A1H9RWD6      K-------ASVEPE--ES------DVMT--IDP-PRPRWFYVDGGW----DMPG-TSIFD
A0A1I0H738      -E------VDIEPE--TL------TVEE----RISEGEHVFVMDFGIN--GSSP-IYVLN
A0A1I2MJ34      P-------NGLEPE--ES------DTAT--IEA-PQAGWFYVRGGW----EVDG-TAIYD
A0A1I7LZ48      A-------ADFEPE--RL------KDGV---ALPADMPRVYVGDFSIGHLNDGR-VHVLD
A0A1I3FQ37      A-------PPLKTE--TL------SIEP--VPK-PGPHWLYLVDAANSHMIDAR-LVIYD
A0A1I3TD09      ----------IEPE--TL------TVKE----TIDEGPNLFVLDQAWK--GPSS-VNVLG
A0A257LQY0      H-------AELPVE--QL------TMEK---LLPIDPYRIYLSDPTMGHLVDGR-AHVVD
A0A255YLN0      T-------AQLVPE--EL------VVES--LPEKLPPHWVWVNDVAFFNMSDGR-AYLID
A0A255Z6A5      R-------AELQAE--QL------SVET--LPEKLPAHWVWVNDVAFFNMADGR-AYLID
A0A291GDG1      ----------IEPE--TL------SVKE----TIDEGPNLFVLDQAWK--GPSS-INVLG
A0A2A4I8K1      A-------PELQPE--TS------DTMR--ADP-PNPNWFYVEGGW----DSPG-TSIFD
A0A2A4B323      P-------PELPVE--ES------DVAA--LPV-LGPRVVMVNTNW----GSTG-ARLFD
A0A2A8HV00      A-------AALEVE--DS------DVAT--IGE-VTPRWVFLTSQW----GRPG-TRIVD
A0A2M6UYR1      M-------EPLPAE--TL------TVS---RIAPEATQRVYVADIAIGHISDGR-IRVFD
A0A2N3LZ45      AE------DAFTPE--QV------SVKE----AIDPGPNVFVYQEEWK--GAGS-IAVFA
A0A2N5EMF6      ----------FKPE--RI------SVEK----NISPGPNVFVLDQNWK--GAST-ITVLS
A0A2N5Y225      P-------PPLPVE-PIG------IIET--LPSVYPASWFLVHDVAFFHMSDGK-VWVID
A0A2I8DPH5      W-------ADLPVE--EL------KGGT--RLPPATPHRLYVMDAAFNHLVDSR-VNLYD
A0A2U3HZK8      S-------AAEKPE--EL------TVAK--VPP-YSPHRVFVTDVSFASMTDAR-VHVFD
A0A2S5KIC4      H-------ADLPAD-TIG------Q-TT--LPFPPEPHRTYVIDAEFENQVASR-VIVVD
A0A2S8RZ29      -E------TDLQPE--TL------AVEA----RISEGNHIYLMDMGIS--GPSS-ISVLN
A0A1H0H1E2      F-------ADLPADKGIG------Q-TV--LAFPPSPHRAYMVDVEFENFVTGR-VVVID
A0A1H2EX98      Q-------AELPAE-ELG------Q-VT--LPFPPEPHRAYVVDVEFENMIATR-VTVID
A0A1H2F3S3      L-------AEIQPE--QA------GVEV--LPAHDGQHWFWIWGNRAPSMIDGR-ALLFD
A0A1H1YFW2      H-------AELPVE-TEG------Q-VT--LPFPPEPHRAYIVDVEFQNMIATR-VVVVD
A0A2T5FZ30      --------VVPQIE--ES------DVAT--LPP-LSPHMLLLTGDG----SIKG-TRIID
A0A323UUH5      H-------AELPVE--IV------TVEQ---LAPPNPYRIYLSDVSIGHIVDGR-LHVLD
A0A2Z2NRF1      P-------PPLPIE-ETG------VVLS--LPESYPESWIMVDEASFFNHYGGK-VIILD
```

A0A315ABB6 P-------PELPAE--TL------TVT---SLADAPSTRVYVADVAISHIVDGR-IRVFD
A0A2Z4UQF9 R-------AADLPE--DP------MGEVVSFSRQNHDRLVWVTDMNMPYQAHGR-GYLID
A0A369T7V0 G-------AKMPVA-EEH------NTV---TLGEPGDHRVYVLDPVFPHLIAGK-VYVVD
A0A3D8JVF2 G-------AVEQAE--DL------TVAK--VPP-YNPHQIDVLDVNFPSMTDGR-IHVLS
A0A398BT79 -E------KDIQPE--TL------AVEA----QISEGNHIYLMDMGIS--GSSS-IFVLN
A0A1W7M7Y5 L-------APLKPE--EL------TTVE--LKEQMPPHWVLVNDISFLHIADGR-AYLID
A0A3S4CAP5 ---------TFVPE--TL------TARE----RLDPGPKVFVNVQNWG--GGPSFVRIYS
A0A3N6NJG1 C-------ATEKIE--TI------TVDK---VPDWNPNWIYGIDDNFNTMAAGT-VQVYD
A0A3N7CFA9 P-------PELPPE--TL------STS---TLDAAQQARVYVADVSIAHIHDGR-IRIYD
A0A3S3TT68 E-------NLTEPE--TA------TSAT--LPA-PSKDWFFVRGYF----ETGG-TSIVD
A0A437J7B1 V-------ASVEPE--EA------NVAT--IPP-PALNWFYVDGGW----GTTG-TSIFD
A0A410GFE7 A-------QHLPVE--QL------TGGH--HLPADASQRVYVMDSVFQHLTESR-LSIFD
A0A418ZRX7 L-------AAGEAD--EP------VILN--APE-PDTRRVYIQDPAH-FAAVTQ-QFVID
A0A423PIM0 -A------DSFEPE--QV------TMES----AIDPGANVLVNQASWD--GASR-IHVFN
A0A401J738 G-------AALEME--TS------DVLS--ITP-PKPTWFFVDGGW----DMPG-TSIFD
A0A4Q7LIS5 P-------EPLPPD-PPL------TTLP--RIDAQHGKRVYVIDLAISHISDGR-IRVLD
A0A4R0XD57 H-------AIEKPE--EL------TVQK--LPA-WHPHEVFVVDISMPSMTDAR-IYLYD
A0A4R6RC52 ---------EFKPE--TV------GLKE----TIDPGPNVFVYQQEWK--GAGS-IAVYG
A0A4V3BNW1 A-------HALEPD--VL------TVTR---MAPADAYRVYIADPNFSSLVDGR-MHVID
A0A4R2KBX4 PA------QDFVPE--TL------SVKP----TIDPGPNVFVNQQQWI--GAGS-IAIFD
A0A4V2SDJ7 Y-------ADLPHD-EIG------Q-AK--LPFPPEPHRAYIVDMEFDTMVATR-VSVVD
A0A4Y8UXA1 ---------SIVGS--VT------SIG--------------EPTERWVSIRGGNTAYLVD
A0A4P7XM83 ----------FEPE--VL------TVEE----SIEPGANLFLLDQSWQ--GASR-LNVLA
A0A4Y8UN28 L-------PPLPIE-TIG------TVET--LPERYPESWVLVDEVSFFKMSAGK-IIVLD
A0A4S1E2M2 --
A0A562NTZ2 L-------AAGKDD--EP------HILE--APA-PDSRRVYVNDPAH-FAAITQ-QFVID
A0A558NPA9 -A------EPFKPE--VF------TVEK----TIQPGPNVFMNEAAWG--GASQ-IHVFA
A0A4Y6V628 E-------PIPQTE--ES------DIIK--LPP-WSAHRILVQDTVARHAKDGR-VYVLD
A0A4Y3TVW3 E-------PILQTE--QS------DVAT--LPA-WGPHTILVVDAVYTHNKDGR-AYVVD
A0A523Q339 P-------PELPAE--TL------GVG---SLAGTSTARVYVADVAISHISDGR-IRVYD
A0A501PGL6 Q-------AELEIE-EIP------NVAT--LPSDYPSDWIFVADGNFNAMIDGK-IVLMD

```
A0A501WV29      -Q------DALPAE--TL------TVQE----RIPAGPHVYVMDMGIN--GSSP-IYVLN
A0A511X7F5      E-------PVLQTE--QS------DIAK--LSP-AGPHRILVMDADYPHAKDGR-VYVVD
A0A511XFQ7      D-------PVLQEE--ES------DVVR--LAP-VTPHRVLVHDEAGQHAKDGR-VYVVD
A0A1I4E4L0      ----------IQPE--TV------GNLT----IPRGTEIVFSVDLALNHVVDGR-VYALN
A0A5D0X323      P-------PPLEEE--VS------DVAV--MGA-PGPHRLMLAGGF----GSGG-AQIRD
A0A6C2CLF7      L-------AELAPE--SI------TTGQ---LPPPHPYRIYLSDVAIGHIVDGR-LHVID
A0A5E4RPP3      P-------PPLAAE--TL------RTAP--MPP-RNPHWLYAIDVAISHLVDGK-VVLYD
A0A6I5YKB7      S-------AALEPE--ES------TVLT--IDP-PKPTWFYVDGGW----DMPG-TSIFD
A0A5S9QH23      -A------EKFQPD--HI------VTQR----QIAAGANVFLNELSWD--GASR-IHVFG
A0A6N6VMG0      A-------DELQPE--TL------HVEK--VPDGPRDHWIWIGDLGAQHNVDTR-AMLFD
A0A6C0U555      P-------PPLPVE-PIG------VVET--LPAEYPKSWFLVHDAAFFHMSDGK-VYVLD
A0A6L6JDP0      ----------FTPE--TL------KVEE----RIAEGPNVLVMDQSWN--GAST-IDVLG
A0A7C9TIH4      Q-------AELPVE--QT------RIEP---LAPATPERIYLTDPVMPHLVDGR-VIVID
A0A6J5FID0      T-------AAEKPE--EL------TIQK--MPA-WHPHEIFIVDPSGDAATDGR-IYVYD
A0A6S6ZV48      W-------ADLPIE--EL------KGGT--RLPPATPHRLYVMDTSFNHMVDSR-VNLYD
A0A6N0WYA5      A-------KPLPEE-AIP------SVTA--LPAQWPKSWVLINDFHFNAIVDGR-VVVVD
A0A7L5RWA2      ----------FVPE--KL------TVEA----KIKPGPNLFVVDSNWA--GSSN-ISVLS
A0A7Y9QWY9      P-------EPLPPD-PPL------TTTP--VIDAKHGRRVYVPDLAISHITDGR-IRVLD
A0A7X0JRG8      Q-------AEIQPE-PLP------VYET--LPKELPDSVLFAHDANFDALVAGR-VVVLD
A0A7Y7IV73      D-------PILQTE--ES------DIVK--LSP-GGPHRVLIEDAVYRHNKDGR-VYVVD
A0A7W4KEC0      S-------PVLQAE--QS------DVAT--LPP-VGAHWVLPAT------DRDA-YTLYD
A0A7W6CEB8      A-------VPTEPE--TP------NVAT--LAS-PNPGWFYVRGGF----DTAG-TQIVD
A0A840SJ55      L-------ASGEAD--EP------VVLN--APK-PDARRVYVQDPAH-FAAVTQ-QFVID
A0A7W6DDQ9      P-------HGLEPE--ES------DIVT--IET-PKPDWFYVRAGW----GIDG-TAIYD
A0A7W4J5F5      T-------PVLQAE--QN------DVAT--LPP-VAPHWFLPAT------AHTA-YTIYD
A0A7W5ZZB3      V-------PVLATE--DV------TLVE--LKDRMPPHWVVVNDVVFTHLIDGR-SYIVD
A0A7W7B107      P-------QPLAEE-PIP------VVET--LPERYPDSFVFVHDLHFKSIPDGR-AALVD
A0A848G7N7      ------------------------MVK---MAPPTPQRMYLVDPAFNHMIDGR-LHIVD
A0A853FCE1      E-------IQAPME--KL------TGGH--SLPEDATHRVYVMDSVFQHLTDSR-VNIYD
A0A7U7EL64      F-------ADLPVE-TIG------Q-TT--LPFPPEPHRTYVVDVEFENLVAGR-VVVVD
A0A850PC06      D-------PILQTE--EN------DIAT--LPA-IGPHVVFIEDPVYRHAKDGR-VLIVD
```

A0A7R7HAE8 ----------IQPE--TV------TNMT----IPDGTELVFSVDLALNHVVDGR-TYVLD
A0A967B6I1 E-------PVLQTE--QS------DTVK--LAP-WSAHTILVVDAVYQHNKDGR-TYIVD
A0A936Z330 Q-------AQLAVE--TL------AMEP---LAPADAHRIYLSDPTMPHLVDGR-IHVID
A0A931HFP9 P-------HGLEPE--ES------DTTT--IAP-PSPDWFYVRYGW----DADG-TAIYD
A0A917GPW5 ---------SVVGG--TS------IIE--------------GYDRYWVSVRSGGTVYLVD
A0A919AKV3 A-------DDLPVE-PTP------NVVT—LPSEYSKDWIFAQDLNFVEPVGGK--IVVLD
A0A974Y937 -----------------M------TVGK---LAPAHPYRLYYTDVALPHLADGK-LTVID
A0A939DFR0 P-------PPLPIE-PIG------VIET--LPATYPESWFLVHDAAFFHMSDGK-VYVID
A0A916IQB5 A-------EDLPPE--TL------TVAP---LPPADENRLYISDVAFRHMVDGR-LHVVD
A0A8B6X9Q5 G-------RAFEPE--TI------DIAT---LSGDASQRLWLPDAVLSHIADGR-LLVVD
A0A940SI03 H-------AIEKPE--EL------TVQK--LPA-WHPHEVFVVDISMPSMTDAR-VYLYD
A0A9N8RZ47 A-------AVEKPE--EL------TTAK--MPA-YSPHQVYVIDANFPSMTDGR-VYVVN
A0A977SHT6 ---------EFVPE--TF------SVKE----AIEPGPNIFVGKQEWG--GASS-IEVYS
A0A9J6RMI2 M-------PPLAIE-EIG------RIEK--LPASYPEDWVLIDESSFFNMYGGK-VIILD
A0A9Q3YNS7 Q-------AQLPVE-KLG------QNTT--LPFPPDPHRSYIVDFEFNNMVATR-VVVVD
A0A9X1DC07 V-------RAQEGE--TS------EVSK--MEP-PKPEWVFVRGGF----GSEG-TSIYD
A0A9X3U0E8 P-------EPLAVE-QTP------NVLT--LPAKYPKNWVFLHDFYFPSIIDGR-VALVD
A0A9W6JCY9 L-------AAGKED--DP------VVLE--APA-INPRRVYVNDPAH-FASLTQ-QYVID
A0A9X3H996 P-------APLGEE-PIG------VVAP--LPAQWPASWVLIHDFNFASILDGR-VYVID
A0AA41Z7Q8 T-------APLPEE-TIP------SVTP--LPAQWPKSWVLINDLNFNAIVDGR-VVVVD

(D) d3pnlb1_{AS}_139_167_9.661e-06 mean AL2CO score = –0.62(±0.36)

d3pnlb1 mfrdgadgvisrgkaepgdktmcdvwvpvveslrqsseqn--------l-svpv-aleaa
P76014 MFRDGADGVISRGKAEPGDKTMCDVWVPVVESLRQSSEQN--------L-SVPV-ALEAA
K8E7T6 ILQAGLDSIEKRGKAVAGEKTMIDTWVPVIEAIKTNK--------------------LT
Q1R0P1 MLAEGRQSLEQISDASVGDKCLLDTLVPAIDGYRHALDAG--------E-DFPQ-ALQAL
A5G1S6 VLAAGVAGVVARGKAAPGDKTMIDALTPAVAALEAAA-------------SLPE-ALARS
A7MG44 ALLAGLAQMKHYGGAAPGDRTLIDALQPALEALRDK------------------GIAAA

```
A7NKW3          AVEGAINGLMARGKANVGDKTMVDALVPALHALKQAIADG--------I-DDHE-ALRQS
D9QAF6          LIDGALEGITARGKATPGEKTMVDAWSLASAAARSAADNG--------A-TAAE-VLARA
Q1YEP1          AFCAMAKGIQDRGKAERGDKTMVDAWLPAAEAIGEAAERG--------D-DLAA-CFRAA
D1CI02          GLRDAIEAIQQLGKAQPGDKTLLDALVPFVEALEEGVRGG--------Q-DLRS-AWRQA
A0A0H3DEV8      ALRAGLEGVQARGKAVAGDATMVDALIPAVAAAEKAAGDG--------G-DVAA-LLTAA
A9KNP0          VLDAMLQAIISRGNAQKGWKTMIDCLAPAVECYQACIAQG--------L-DEKV-TADQV
D7C870          ALRTGVDAVAQLGGAAPGDKTMLDALLPALDALAT----------------DGG-SFAAA
D7DPP9          AFAGGVQAIIQRGKAGLGEKTMLDVLIPVSNEFVLQATQN--------N-DIKL-ICAKL
Q82NL7          ALRAGVDAVMQLGGAAPGDKTMIDALVPAVDALSD--------------------SFAAG
F9USY1          LLAAALAGIEKRGGATTGDKTLIDVWAPVVQAVQAGE---------------------LT
B9DU17          ILSEGLAMIQKRGKAEKGEKTMVDVWIPVIEALG-QGQLE--------K--------ETI
F8A062          LLEAGLDGIVARGKATTGEKTMVDAWSPAADAAAKAAAEG--------A-DPRG-VLVAA
Q5HKK0          VLKAFADGIQQRGKVELNEKTMYDVIERAREAVEK----------------NETVDLDKL
Q4L473          VVTEFANAIAQRGKVQRDEKTMFDVVDRAREALEN----------------DETITSEIL
Q8R8K0          IFEAALQGIKDRGKAQAGDKTMIDSIEPAYKALKESLENN--------I-ALPE-ALKRA
Q8XDH5          MFRDGADGVISRGKAEPGDKTMCDVWVPVVESLRQSSEQN--------L-SVPA-ALEAA
A6L9P2          MFAGGLANVQQQTQAKRGDKTMMDALIPAVEAMQACTSDD--------I---KE-VLNAG
Q6LL47          MLQDGMDGIVQRGRAEVGDKTMIDTWNGIMTSLKQD--QN--------I-SVYD-ALNTA
Q63NN9          ALAAGFAGVQAISDAKIGDKTLIDTLAPAAAAFHEALRAG--------D-DFRH-ALAAM
Q65S46          VLKDGVAGIVSRGHAELGDKTMCDVWLPVVNQLEQE-DGN--------Q-PLDA-LLKSA
C7R307          LLVAACEGIEMRGHAVTGEKTMVDAWESAVKAAQVAALSG--------A-DTQQ-VLVAA
A1SLY2          FAAAAANSIAQRGKTQVGDKTILDGIHAAAETLRSASDSG--------Q------AAAAV
B8D1L0          MLNAALEGVKALGDAKVGDKTMMDTLIPAVKTYDKAIEGG--------K-GFKE-ALEEM
B7LGV9          MFRDGVDGVISRGKAEPGDKTMCDVWVPVVESLRQSSEQN--------L-SVPV-ALEAA
B7MKA1          MFRDGADGVISRGKAEPGDKTMCDVWVPVVESLRQSCEQN--------L-PVPA-ALDAA
A0JRA4          AIQAARDGVVARGKAESGDKTMVDAWTPAVDAAAAQAAAG-------DG-DVRK-VLLAA
A0KQ90          MLSDGVEGIVSRGRAEPGDKTMCDAWWPALAALQQAEQQG--------L-PLPE-ALDKA
A0QU92          ALRAGVDGVVQRGRAEANDKTMFDALAPALDALDAALEGD--------T-DLPG-ALAAA
C7LYL5          TLSLFRDAVLERGHAELGDKTMVDVLVPALASLAAELGTQ--------R-SFAE-AAATA
Q9CKH0          FLVAGSSAVFARGGAKPGDKTMVDALVAAEKKAEEVSHE-----------PLNV-ALLSI
A0A3N4B7H8      MLKGGLADLQEVTDARVGDKCIMDTLIPASAAFELAQQQQ--------Q-SFVE-SLHRM
```

Q8YA19	MIINGAAAVQHRGKAELGDKTMMDAFLPGVEVLQNRDANA--------E-PI-E-TFSTF
Q8DY42	ALGAGLEMIQKRGKAELNEKTMVDVWHGVIEAIEKNE---------------------LT
Q8ENK9	GLQAGLDGIKQRGKATTGEKTMIDVWDPVVQLFKDDE---------------NV-APSTI
C7MWZ8	ALNAGLDGVVARGKAEVGDATMVDALKPAVEAAQAAKKDG-----------VAA-MLSAA
D1AJM4	AVKEAVEGIKQRGNASVGDKTMVDTIEPFYAAFKKAVQEE--------K-NLKN-SFAEG
D5UD19	LLEAALDGIVARGRAGAGEKTMVDAWTPAVAAAVEAADHG--------A-PPSA-VLAAA
C7Q5T1	AFDAGLAGVQKLGAAVPGDKTMVDALVPALDAFGAAARAG--------E-SDAA-AAEKA
D7GCR5	ALRAGLEGVTQRGKSAVGDKTMVDALAPAVERLEADAAAG--------D-KLAN-ALKHA
A0A384LQB2	MFRDGADGVISRGKAEPGDKTMCDVWVPVVESLRQSSEQN--------L-SVPV-ALEAA
Q6MTW9	CLNDAVSGIKLRGKANIGDKTMVDVLEPVSDLIKELINKN--------E-SAKE-ILNKA
B2TZU4	MFRDGADGVISRGKAEPGDKTMCDVWVPVVESLRQSSEQN--------L-SVPV-ALEAA
C3KP63	ALEHAIDAVARRGRASPGDKTLLDVLYPVHAEVV----RR--------A------GLAGI
A6VPA2	VLTNGVTGIISRGRAELGDKTMCDVWLPVIAELEA--NQT--------E-PEVK-LFERA
A7ZKX1	MFRDGVDGVISRGKAEPGDKTMCDVWVPVVESLRQSSEQN--------L-SVPV-ALEAA
A0A7I6GZ14	MFRDGADGVISRGKAEPGDKTMCDVWVPVVESLRQSSEQN--------L-SVPA-ALEAA
Q5WJG3	ALKQAAEGIAQRGKAAPGEGTLLDVWSTVANEAK-ETGMD--------W--------NQL
A9H2V8	LLAAGVAGVAARGKAVAGDKTMLDALIPAVAALSGTQSGP----------DTPQ-ALARA
B2J6P9	LLQAGLDGVLQRGKAQLGDKTMVDVLSPAVTAFGQAVGES--------K-GTLE-AMQQA
B2JWR9	VFAAGVDAVAQRGKAGIGSKTMMDVLIPVASRFAEMADED----------AAPEAVLDAL
K5Y1C8	MFAGGLANVQKQTKAQKGDKTMMDALIPAVEAIQACPSDD--------I---KE-ILEAG
Q9K7G5	SIEAGLNGILKRGKATTGEKTMVDIWKPVVESLMAEQQLS--------K--------ERI
G2SM22	LLQEALSGIKKRGGAQPNDKTMVDVWNKVVSKADDSS---------------------LT
Q6AFI5	LLTAARDGILSRGRAEPGDKTMIDAWTPAVDAAAAAQAAG--------E-SPSA-ILAAA
B0S302	ILKITTESVKLVGNCKLNDKTILDCIYAAEKSFEENKNEN--------T---IR-LMKKI
B8I2J6	TLEAALKGMQAFDKAKVGDKTLMDTLVPAIEGYKAALAEG--------K-DFKA-ALEDM
C8WGF7	MFAGCLAEMQDITTAQVGDKTMMDALIPAVKAAQACAAPD--------D-GPVD-VLMAA
D2SH30	ALRAGYDGVVARGKAERGDKTMLDALGPACDALDEALAAG--------K-ALDE-ALGAA
G2NB77	AFGAGVAAVAQLGGAKAGDKTMLDALLPAVEALGE--------------------SVGAA
A0A0H2V747	MFRDGADGVISRGKAEPGDKTMCDVWVPVVESLRQSCEQN--------L-PVPA-ALDAA
Q893Q0	ILETSLEGVKARGKAQRGDKTMIDAIAPSLEVVKNGIKEG--------L-DEKK-ILENM
Q835L6	IVQQGLAGIQMRGKAEPGDKTMVDVWAPVAEVIGTEQ---------------------FN

```
Q7NC54        SFYRALLDCKNKYQLRIGAKSFYDILYPVLKYLYLNNDLS--------T---QE-LMAES
A3MYF4        IFKAGVEGIIARGRAEPGDKTLCDVWLPLLDELAQA-DHS--------Q-PEAV-LLNQA
C9YYH4        ALRAGVDAVRALGGAAPGDKTMVDALVPAVDALAE--------------------SFGAA
C4LG95        VVESAVGGVEKRGKATEGEKTMVDAWAPAARAARSAADEG--------K-SPAE-VLRAA
D7DWU8        FLQSGLDGVIERGKAQLGDKTMLDVLSPAVVAFEQAVNEG--------M-GTVE-ALRVA
A6TVH6        VLEAELNGIMERGKAKPGYKTMIDALYPAVTRFKESLEKG--------L-DNHT-ALQEL
D9TSY8        MFEAGLSGIKERGKAQAGDKTMIDAIEPALNELKKSLEDG--------L-KFNE-ALSIA
Q03BZ6        LVDAAAKGIAKRGQAKTGDKTMLDVWAPAADLIK-QGKLD--------D--------AAI
D9QZW4        LFEAYEAGVADLGKAKLGDKTFLDGFHPGVQTLKAELEAG--------V-SLRE-AFGKA
B1YE66        AFKEATDGIKMRGKSEFGQKTMVDIWTPFYEALEQESDLN--------A-----------
D4GL25        MIREGAEGVINRGKAEPGDKTMCDVWLAVVASLRQSSEQN--------V-SLTA-ALDAA
D5E5M2        AFSFANENLKARGKTEYRNKTMYDILFAVEQ----ATKDV--------K-DFNE-FVKKA
Q2RUQ3        MIRAGLDGVEEIGSAKPGDKTLLDSLAPAVDAFDAAQAEG--------R-SFAE-SLDAM
Q2JZR2        AFAALAQGIQERGKAEPGEKTMVDAWKPAADAALAAWASG--------T-PLSQ-SLDMA
I2BC43        MIREGADGVVSRGKAEPGDKTMCDVWLPVADALRRASEQH--------L-SITD-ALEMA
Q9FC12        ALRAGVDAVRTLGGAAPGDKTMVDALVPAVEALGD--------------------SFAAA
Q92WN5        LIQAVASGIGERGKGQRGDKTMLDVWLPAADAAAQAVAAA--------K-STAA-FWADV
Q12FM9        ALAAGVEAIKSRGKADLGEKTMLDVLIPASRLLLRLAEEG--------C-DQGT-LCARL
A4YWS0        ALGRAIEDVKARGKSEAGQKTMLDVLIPVQAAFAADG---------------DD-LATRL
A9WUM9        VLTAARDGIVARGKAEPGDKTMIDAWTPAVEAADAAVENG--------L-AADE-VLAAA
A9W9R1        ALEAALAGIVARGKAQRGEKTMVDALAPAIEALQSALAEG--------K-SLGR-ALLAA
D1C9M9        AFDAALEGIKARGKASPGEKTMVDALTPAVDALRAAVEQG--------L-DLPT-ALDHA
D2B9Y6        ALQAALAGVQRLGSAAEGDKTMVDALAPAVRALSRATQEG--------K-DPEE-AIGAA
D1B6Y5        AMAEGVRGIVALGKAEKGEKTMLDALYPALEALSSGSDEA--------S------ALEGA
A0A140NCI1    MFRDGADGVISRGKAEPGDKTMCDVWVPVVESLRQSSEQN--------L-SVPV-ALEAA
C3PGU3        LIEAALEGITVRGKAAEGEKTMVDAWAPAARAARGAADAA--------K-SPAE-VLRAA
A9G640        CLEAGLDGVKRRGKASPGDKTMVDALSPAVDALKAALSAPAPTGDPARS-SLMD-GLARA
A3CK11        VIQAGLDMIQKRGKAVPGEKTMVDVWSGIPISLQ-SGDLT--------L--------EKI
B0K761        IFEAALDGIKQRGKAEAGDKTMIDSIEPAYKALKESLENN--------I-ALPE-ALNRA
Q72DE9        MLEAGVAGLVSRGRPVLQDKTMYDAWAPALDAFRAALGKG--------D-DLAL-ALDAA
Q9CIV7        LIYAGLEAIQKRGKAQVGEKTMVDIWSAFLNDLQTDS---------------------AS
```

Q6NEE0 LIAAALEGIQARGKASEGEKTMVDAWAPAAHAAATAADEG--------A-SPAG-VLARA
C6WHH1 ALRAALDGVVARGKAQVGDKTMVDALSPAAAAAAGLGGAG-----------VGE-VLAAA
C8XFT9 ALTAGRDGVVARGKAELEDKTMVDALTPAVNAARAAADAG--------G-DVAA-VLRAA
D7AWT1 ATANGWATLHRLGSAEVGDRTMVDAVAPASEALAGAVASG--------A-GLHA-GLRAA
D1BZN2 LLEAALEGITARGHATTGEKTMVDAWHPAAEAAA--AADG--------Q-PVLA-VLRAA
A6X6C1 IVEAVTRGIQERGKGQRGDKTMLDAWIPAMEVAIDARTHS--------L-ASQE-MWSKI
E1R8P9 AFQAGLEAIKKRGKAHVGDKTMLDALVPAVEKMEVLAGEG--------K-DIPT-MLGGA
E2Q762 ALREGVTAVAGLGGARPGDKTMLDALEPAVVALAG----------------APE-AFGRA
B7KD17 LLQAGVEGVIQRGKANLGDKTMVDVLTPAVEVYQKAIAQG--------D-TIKE-ALVKT
A5N619 MLEQALVGIKMRGRGKLGGKTMIDAIEPAVNSLRYSISKG--------L-DSVK-SLENV
Q1H2B4 AFHASVEAIKQRGKADAGEKTLLDTLIPVADTFSRLTAVN--------A-EPAQ-VFDAI
E3J2C4 GVDAGTATVQRLGKARPGDKTMVDAMVPAVGALAAAVADG--------L-DLAD-GVALA
Q87SQ7 ALKSGVDGVVSRGKAELGDKTMCDVWLPVLVSVKASLENG--------M-SADH-LLNEM
Q881Q7 --------MQDITEAGIGDKCLMDTLIPAVEAFEQAHANG--------A-SFNE-ALDAM
Q8RHZ7 TLNSMIEGIQKRGKAVLNEKTMLDTIMPTYNFLEKSFNEG--------K-NLKD-IKNEV
Q8EW70 IFNQMIEAIKTRGNSQVGEKTMLDTLVPAQQAFKKAVEEN--------K-DIVE-CFKEM
Q89PB0 VFGSGVNAVSARGRSQAGEKTMLDVLVPVLETLRTA--AG--------QP----DLIARV
C6BY70 VIEAGVEGILQRGRPDLGDKTMYDLWAPVLEVVKEKAGNG--------D-DVLA-IVEAA
H0TUV4 ALGRAIDEVKARGKSDAGQKTMLDVLVPVQAAFA----TG--------GA----DLTDRL
Q313B6 LLESGVEGIVSRGRPVEGDKTMYDIWKPALDAYTQAVQAG--------A-ALDA-ALDAA
Q32H07 MFRDGADGVISRGKAEPGDKTMCDVWVPVVESLRQSSEQN--------L-SVPA-ALEAA
C6WWI2 AFTAGVEAVQQRGKAGLGEKTMLDVLIPVAQQFSALAASN--------A-DIKT-TCQAL
C6XCW2 LFATGVAGIQHRGKAQVGEKTMMDVLIPVSALFTQLASTH--------A-EIDADILNAI
A0A094YZF9 ALREGEKALRQRGKAVLGEKTMIDVWSPVVAELEKANSLN--------H--------EKM
F4XFL5 MFAGCLSEMEDITTAKVGDKTMMDALIPAVQAAQQTQGDC------------KA-ILEAA
B5HKC0 ALRTGVAAVAQLGGAKAGDKTMLDALEPAVAALGD----------------STE-SFAAA
I2F888 LFQAMVDGVQKVGKAQLGDKTLLDALIPFKESLKESAKLN--------E-PIEA-AFDKA
I6RZA4 IIEEGKNGIQARGKAEAEEKTMLDVWLPVTEALK-NNRLT--------E--------EVI
A3YTX8 ALRQAVDAVKARGRSDAGQKTLLDVLDPVHAALA----RG--------A------TAAEI
J0MWJ2 AVEAGVGGIVQRGRAEAGDKTMLDAWFPALDSLRSG------------A-DLRA-ALQAA
E5BF30 IFKAMIEGVKKRGKASLGEKTLLDTVLPVYDLLQHRLEQG--------E-DILS-NTEEI

F3NT86	ALRTGVDAVRQLGGAAPGDKTMIDALVPAVDALGE--------------------SFDAA
E0QM99	VLHAGLQGVLDRGKAELQDKTMVDAMTPALEAFDAVAATG-----------LTA-AATAA
C4IDX1	MLNEALEGIKMRGKSVEGEKTMIDTLSPALEASKKALEEG--------K-SYED-VLSAL
I9GRJ3	MFAGGLIGVKKQTKAQRGDKTMMDALVPAVEAIQATNSSD--------I---KE-LMAMG
H0HYA5	ALAKAIDAVAARGKSQAGQKTMLDVLYPVQTAVA----AG--------K------TTPEV
F3B1P8	VLKAMLEAIMSRGQAKAGYKTMIDSLYPAVKAYEEALREN--------D-DDKT-LCEKV
G5K1Y5	ALQAGIDMVQKRGKAEVGEKTMVDVWTPVIKSLKEET---------------------LI
F3PPM2	MFAGGLANVRRQTKAQKGDKTMMDALIPAVDAIQSCPSDD--------I---RE-IMNAG
F5T169	VYSAGVESIKARGKADIGEKTMLDTLIPVMQSLQSDTEQN--------A-DKTT-VLSNI
H3NBU3	ALEAGRDGITKRGKSSVGDKTMVDVWTPALELLADGD---------------------VS
G9PIG6	ALEEATSGLEARGHATSGDKTMADAWRPATVAAREAAEIG--------E-DEVG-VLSAA
A6EMJ5	SIESGLKGIKSRGRASLGEKTMVDVLEPAVNSISASINSN--------A-SMEQ-ALTHM
W0IWI1	MLAGAMTGVSGLGDAKVGDKTIIDTLAPAEAAYREAIAAG--------A-TIPD-ALARM
C7HTR7	AFSDGVDGVLMRGKAQVGDKTMVDVLYPVAEALKND-----------------S-SFEEI
H0QJK9	ALAAARDGIVARGKAESGDKTMVDAWTPAADAAAAAAADG----------DTLA-VLIAA
A4AIN0	ALRAGLDGVVARGKAEAGDKTMFDAINPALDAWDAAVAGG--------A-DIAA-AASAA
F0Z4L2	YLDAGRTGLYEIVDARIGDKTLVDALSPAVDALRQADGKR--------E-SFAA-ALDEM
K0YPL4	LIGGALAGIQARGKAERGEKTMVDAWGPAYDAAKAAAESG--------A-TPEQ-VLAVA
I8UJC4	GFQEACAGIAARGKSEVGQKTMLDVWVPFTEALA-TRQNV--------D--------ETI
C8PCR8	LLEAALAGIKKRGGATIGDKTMVDVWSVVIEAIKEDA---------------------LT
C2L0R3	IFSTSLTSLKKRGKAKVGDKTMVDALEPAVLALEEGAKEG--------L-DLIE-GFQRA
D9X7S5	ALRAGVDAVMTLGGAAPGDKTMIDALVPAVDVLGD--------------------SFAAA
C6PVN6	VMNAALGGIKMRGKAELEDKTMVDAIVPAIESLNESVSSN--------E-DTVI-ALEKA
G2GMN3	ALRAGVDAVMALGGAAPGDKTMIDALVPAVDALGD--------------------GFAAA
D0GNQ9	ALEAGISKIKERAGSKENEKTILDALVPATEKFEKAIKSD--------K-TISE-ILKET
G6FMN8	MLQAGLDGVVGRGKAQLGDKTMVDALSPAVTAFGQAVTAG--------E-NILS-AMQKA
E0NGR7	LLVAATAGVQKRGKAILGEKTMVDVWEPISEKA---T---------------------FT
C6JM61	TWNEMIKGIEMRGKAVLGEKTMLDTQIPAYEAFKLKADAG--------A-GIKE-CFEFA
D6U6Y4	LLAAMVEGIQARGKARRGEKTMLDALLPALEAARKAESEE--------L-EMPQ-LLQVC
G3ITN9	IFTSGVDAVKQRGKADAGEKTMLDVLIPVAASLRQAAADS--------V-ALPV-ALDNV
E6MJW9	MFEAAIGGIQQRGKAVAGEKTMLDAVMPAKEAYDAGIAAG--------K-SIAE-ALADA

C0W3K3 AMEAGVAGVQARGRSQAGEKTMLDAWLPALEAMRGQ------------S-DLAA-GTAAA
C8NCE6 AIADGSAGLVARGQAAAGDKTMVDVWLPVAEVLKQAAASG--------V-SLKD-ALQAA
E8LGV2 MLAQAKEDFEDISKAKIGDKTMVDALYPALEAGMTAEG------------DAKA-VFAAM
H5X503 VLRAGLEGVVARGKAEVGDATMVDALSPAVEAAEAAKGEG-----------IAA-QLAAA
A6DNZ7 SFESGLARVMKIGKSAEGDKTLIDALAPGIRALKASSQAG--------E-SLGV-ALNKM
B1FVY6 MFAAGVEAVAQRGKTGVGSKTMMDVLIPVAARFKELAAVE--------SPALRKAVLDEL
F9QEG7 MLLAAAEGIKMRGKANVGEKTMLDVLEPFATTFDQLTKNG--------E-KNKS-ALEKA
I1D5G2 ALRAGLDGVVVRGKAEVGDATMVDALKPAVEAAERAEGDG-----------VAA-VLSAA
H8GRD8 AFAQGVESVKLRGRADAGEKTMLDVLIPAAQSLQKSAAAS--------E-GLKE-SLAEI
E3CX22 ALQEGLNGIVALGKAQVGDKTMVDTLVPALSTLAAE--AE--------G-DERS-AWDKT
G7M3Z5 MLNEALEGIKMRGKGQAEDKTMIDAIEPALNAINEGIEKG--------F-EVKE-ILRTA
B1CB33 MMEEAINGVKMRGKSEAGEKTMLDAMIHAYDAFKEG--LC--------D-GAKS-AIKKA
G4CVL8 ALDAGLAGIVARGKAEVGDKTLVDALSPAVDAVKKVAADG--------G-SAAE-ALAKA
F9EGT1 AIGAGVSGIARRGRAEAGEKTMLDAWYPALDALRAS------------T-DLVP-ALEKA
I8TY96 MLEAAIAGIKERGKATTGEKTMLDALVPAYEAFQDGAKTG--------K-SVIE-CLELA
G5IBQ9 MLKGSLEAMEDITTAKVGDKTMMDALIPAVEAAVAAGQAG--------G-DVMA-VLTAA
G5KIC1 HLQAALEMIQKRGKAEIGEKTMVDVWIPVIEALKTDS---------------------LT
F9ELQ8 ALNSMIEGIQRRGKAVLGEKTMLDTIIPTYNFLEKSFNDG--------K-SLKE-IKNEV
A8RYY3 MMDTAVQAVEQRGKATVEEATMLDAMVPSLKTMKDAAAEG--------K-SVRE-ALEAG
G8TSR2 AIDVFVTALKERGKAELGDKTMMDVWLPARETLHERLDAG--------D-SGWE-ALQYV
G8S8S8 ALHAGLEAVRRLGGAAPGDKTMIDAYAPALAVFERSVAGG--------A-DLAG-ASAEA
G8PBG1 LIPAGLAGIERRGNATEGEKTMVDVWKPAAEAVSAGK---------------------LT
F7KPB8 MLAAGLEGLYGIVDARPGDKTLVDTLNPCVECVKSAAEAG--------K-DYAV-ALDEM
G8QU73 LFQAYFDGVANRGKAKIGDKTFLDGLNPAIEALKDSIGEG--------K-TLQE-ASQDA
D4E0S0 MMQEGVEGVVMRGKAEPGDKTMCDAWWPVVGSLRQSAQQQ--------L-SVAQ-ALQRA
F1T4X2 MLQCGLDQIQKRGHAELGEKTMVDFWSPAIEQLRRGE---------------------LS
I4BYG6 AFREGLQGVVALGKAQLGDKTMVDALTPAIEALEAAAKQG--------L-PLKE-GLKRA
J9Z1A9 IFAQGVEAVKQRGKADVGEKTMMDVLIPVANCLQDGVQNN--------K-EINE-ILTEA
F9ZQA6 ALAAGLAGLQRLGKAEKGDKTMVDALLPAIDTLQSS-----------------A-DLQKA
I1A7E4 FVSNFAGSLEMLGKVTLGEKTMYDVWKPLSEKLNPLPEIS--------E-NTKKELVNYI
C9MZ82 ALENGISKLKEKANSKEGEKTILDSLCPATKVFANNIDKG--------K-TVLE-ILKEA

K0K2T4 ALQAALEGVVARGKAVVGDKTMVDALTPAVAAAESVASTG--------A-DVAG-VLTAA
Q49VJ6 LLNSFSEAIAQRGKVELNEKTMFDVIERANQAIQQ----------------GENLTLDVL
Q5FJ65 LLDAALAGIQKRGGAEPGDKTMVDVWYKLVPDIKDGS---------------------LS
I3D7P6 MLKNGVEGIVSRGHAKLGDKTMCDVWLPVLDELEQA-DLH--------Q-PLCT-LLESA
I4F4L6 ALRAGLDGVVARGKAEPGDKTMLDALAPACDALDAALAEG--------R-PLGA-ALTAA
Q1WRP5 LMELALAGIKKRGGAEAGDKTMVDVWTVLVPKLKDNS---------------------LT
D7A0Q6 TLGRAVEAVARRGRSGPGDKTLLDVLYPVQAEVA----GR--------A------APARI
Q1AX45 ALEAALAGVKQRGRAEVGDKTMVDALEPAVRAARQAEGKG-----------VAA-MLRAA
A4FMZ5 ALKAGMEGVAARGKAVTGDKTMIDALSPAVDAAKAAADAG--------E-GPVK-VLSVA
D6ZIF3 LLVKAAEGIAERGGAQPGDKTMLDAWYPAATNAAAAVAKG--------A-TLEE-VLRVA
Q60CU3 AFGRGVEAMKARGKADRGEKTMLDVLIPVADVLKSAAEHA--------I-APGQ-LAEEV
A8LNW4 LIAGARDKMQERGKAELGGKTVLDSLDYVARTTDKADDK-----------------ASAA
F2N7D3 AFHAGVLAVHNLGKAEIGDKTMYDVLAPVDDLLAGWRGEG--------E-GRHA-SAAEV
F2R6Z8 ALASGVAAVSQLGGAQAGDKTMLDALGPAVEGLRE--------------------SFAAG
E3DLJ7 IGEEAVAGVQKRGKAEQGEKTMLDTMIPAVNSLKESKEQD--------H-DLET-ALKKA
E3G7K1 MMREGADGVINRGKAEPGDKTMCDVWLPVVESLRQSSEQQ--------L-TVAQ-ALQTA
E3GPD1 ALEAAIEGIKQRGKSTTGEKTMLDAIVPAKEAYDKAVGEG--------K-GIAD-ALADA
E3H7Q4 ILKASIDGIQMRGKAVKGEKTILDSLIPAYEAYEKSLSDE--------E-SGTK-ALEQA
K2QR18 FWRAYTDGIAERGKAKVGDKTLLDVLDPMTKALEAAAAQG--------D-SLTM-ALSSA
H6LKL8 AFDEAVGGIQYRGKAVKGEKTILDSMIPAIDAIKTSIANG--------K-TMTE-ALVDA
J2ZU17 LLAAGLAGIQKRGGATTGEKTMVDVWAPVVAAAK-AGNLT--------E--------QVI
J2VAZ1 AFIAMAKGIQDRGKAEIGDKTMIDAWVPAAEAVEAAKARG--------A-SLTE-LLDAA
K2N9I0 IVEAIRDAISARGKASVGDKTVVDSFDYLTRYLAQGPVS-----------------LETA
I0JQA6 ALKEAVAGIKQRGKAEAGEKTMLDVWEPLADKMSEVQ---------------DF-DRQLI
E4RNI0 IGQSAVDGIQKRGKAEQGEKTMLDAIIPAVDALKESKENG--------D-DLEE-ALQKC
F4CPQ6 ALRAGLEGVVARGKAEAGDKTMYDALSPAVDALDAALADG--------D-PLAE-ALRAA
A0A0H3GYP2 MIREGADGVVNRGKAEPGDKTMCDVWLPVVDSLRQSSEQH--------L-SIAA-AMDAA
F8JV61 ALRAGVDAVAQLGGAAPGDKTMLDALVPATEALAFALDAG--------A-GLAE-ALAAA
I0I650 VLEAGRDGVVQRGRAELGDKTMIDAWTPAVAALREALDAG--------Q-PLSA-ALRAT
I0HCV6 GLHAGLEAVRKLGGAEPGDKTIVDAWKPAADAFEAAVAAG--------G-DLTA-AAGEA
F5X6F7 DVKAGLEMIKKRGKAEIGEKTMVDVWEPVLKDLETNS---------------------LT

F5XQ38 MFRAGVEGVAARGKAVLGDKTMYDALAPACDALDAALAAG--------S-DLSA-ALDSA
C5BWZ3 LVDAALEGIVARGRAQVGDKTMVDAWTPAARAAQEAADDG--------A-APAG-VWAAA
D2PZT9 ALKAGVGGILARGKAELGDKTMYDAWAPALDAYDEAVAGG--------S-DLRG-ALVAA
K4RFM8 AFRTGVEAVMTLGGAAPGDKTMIDALLPAVDALDA--------------------GFGAA
A8AFT8 MMREGAEGVTSRGKAEPGDKTMCDVWGPVVESLRQSCEQH--------L-SVQA-ALDAA
B7UQ88 MFRDGADGVISRGKAEPGDKTMCDVWVPVVESLRQSCEQN--------L-PVPA-ALDAA
E0U9C1 LLQTAVQGVIQRGKASLGDKTMIDVLVPAVGRYQEAIARE--------D-TIQE-ALFKT
Q73K63 GFSKFNEEIMKKGGAKPGEKTLVDTLVPVEKFVRENLGK---------I-PDRE-LIKES
A0A0H2YY24 MFRDGADGVISRGKAEPGDKTMCDVWVPVVESLRQSCEQN--------L-PVPA-ALDAA
A0Q3F2 ALKEALTGIKMRGKADIDDKTMIDTLVPAIDVLEKALNEG--------K-NDLD-ILKDV
D3F3G8 ALEAGAAGVQRRGKAEPGDKTMVDALLPARDALATAAADG--------A-SLAE-ALRAA
D3R2L5 VLNAMLTDIQERGKAKPGMKTMIDALAPAVERFKLGLVNK--------E-NIES-IFSEI
F4BL99 VLEAGLEGIQKRGKAVAGEKTMVDEWIPVVEAVKSKT---------------------LT
F4F7H7 GLGSALAGIRELGGSAPGDKTLVDAIHPAVAVLESPSTGS--------A-GLVA-ALDRA
G4T0K8 AFAQGVEAVKRRGKADAGEKTMLDVYIPVADYLKQAEVNS--------T-PWTE-ILEQV
F4H6I8 LLEAALEGIVVRGKATTGEKTMVDAWTPATEAAVAAADGG--------A-GPAD-VLAAA
K6UNQ1 ALQAGVDGVIARGKAEPGDKTMIDALAPAVEAFAKASTD-----------GLAA-ALTAA
B0TAR9 MLAAAIQGIKDRGKATRGEKTMLDALEPAFEAFVQGLEAQ--------K-PLVE-CLEAA
A0A0H3EI07 MFRDGADGVISRGKAEPGDKTMCDVWVPVVESLRQSCEQN--------L-PVPA-ALDAA
D6EB63 MFANALAELSDISGAQVGDKTLMDAFVPAIEAIQACEG-N--------E---AA-LFEAA
Q2G0E9 LIQAFAEAVESRGKVTLNEKTMYDVVARAAEKLKN----------------GETLTFNDL
F9ZY89 AFQLAVAAVKQRGKADVGEKTMLDVWVPVAETLKRAVSEG--------Q-QLNV-ILDQV
E6SH85 MFKAALAGIQERGKAARGEKTMVDALGPAVEALEAALGRG--------A-GLGE-ALAEA
F7V0N8 MFAGCLAEMRDITNAQVGDKTMMDALVPAVEAAQACTDDD--------A---AA-VFAAA
D4MT14 AFEAAVEGVKQRGKSDKGEKTMLDAMIPALEAIWQTYGET--------K-DAQS-AWKAG
A0A8B1NT48 GLGAGVEAVAQLGGSAPGDKTMLDALVPGIEALAT--------------------SFDAA
A0A0H3MXE5 TLQAGLDMIQKRGKAELGEKTMVDVWIPVISALRESQ---------------------LT
I1YIS9 IYAAGVAAVKARGKADIGEKTMLDTLIPALVSLQASVRSN--------D-SLMQ-AMQSL
F5YG98 MLRSGLLALYDIVEARPGDKTLVDTLAPACDAMDKDAEEK--------M-NLVA-ALERM
F6EM32 LLHAGLEGVVARGKPEPGDKTMFDALAPAVEAVDDALDDG--------A-SLSD-ALAAA
F6FRU1 MLEAALEGITTRGKATTGEKTMVDAWQPAVDAATRAAQDG--------A-DPVA-VLRAA

```
K9ETT1    ILSAGLSGIQKRGKAEVGDKTMVDLWSGALDLIK-EGQLT--------Y--------QKL
M2XUI3    ALAAGQAIVQRYGEAEVGDKTMVDALAPAVEAMRRSLHDG--------E-PSQH-VLEAG
K9CFB7    LFTCGIEAIRRRGHAERGDKTMLDTLIPIRDAFLPENAEG--------K-SLRE-CLEDA
G0VNU3    AMEAAVDGIKMRGKATEGEKTMLDALCPALKVMQDEVAAG--------K-SLKE-ALQDA
D7UV97    VIEAGQEGIEKRGKSHAGEKTMLDVWEPVVNSLHQDE---------------------LS
C8RYK0    LVVAMADGIKARGQAELGDKTMIDVWYPAGHAAIAAAAEG--------G-SPAE-VARAL
K8WHA8    LIKAGTDGVVSRGKAEPNDKTMCDVWWPVSEALENN--AS--------L-PLND-AIAIA
E6LEU6    AVQAGLEMIEKRGKATVGEKTMVDIWASVLADLQ-QDQLT--------V--------ARV
C0W168    FIEGGLGGIQARGKATTGEKTMVDVWTPALEAAKAAAANG--------A-SAVE-TLEAA
M4U7Q4    MLSAGLDGIIERGRAQPGDKTLCDVWWPAIDAGQQALDNN--------L-PLNI-ALQNI
K9RKI5    LLKTGLDGIIARGKTQLGDKTMVDSLHPAVMAFEQAINDG--------A-DVKN-AVEKA
K9TAS4    LLQAGLNGVLDRGKAQLGDKTMVDALSPAVTAFQTAAAEN--------K-DMRQ-AMQQA
K9U7Y7    LLRLGLEGVIQRGKAQLGDKTMIDVLSPVVDNFQKSVDNN--------Q-TTLV-AMQQV
L1L3V1    ALRAGVDAVMTLGGAAPGDKTMIDALVPAADALRD--------------------SFAAA
L0RXD0    FVANFAKNLQILGKVQLNEKTMYDVWNPFCTKLSEFNEIN--------L-EAKNELLEYL
K9WJ60    FLQATVDGVVQRGKAHVGDKTMLDTLAPAVDAFKDAIAEG--------A-STPE-ALQKA
K9ZQU8    LLQSGLDGVIERGKTQLGDKTMVDVLSPAVVAFEQAVGQG--------M-GTVE-ALQMA
K9XI13    LLQAGLDGVLQRGKAQLGDKTMIDALSPAVTIFTQAINQG--------K-TTSE-AIQHA
K9VA65    MLQAGFAGLVERGKTQLGDKTMVDTLSPAVIAFQQAVNDG--------K-VMRD-AMQLA
L5NDP0    MVQEALEGLKQRGKAEVGEKTMIDVWSPVAEAVKEEG---------------TW-EPVKW
L0EJ60    MFEAMVQGIADRGKARVGDKTILDSLAPATEAMGAARQRG--------A-GLDQ-MLKEA
F4LS03    MFKQGVEAVEFRGKASVGEKTMVDVLRPAVDEFEKAMKEN--------L-APKE-IMEAF
L7FGQ5    ALRAGVDAVMALGGATPGDKTMIDALVPAVDALTD--------------------SFAAA
L0KF84    AFGKAIEAVAARGKSQVGQKTMLDVLRPVHDALA----GG--------R------TAAEI
F4EWM0    AFSASVAGIKMRGKSTIGEKTMLDALCPAERAMREAVEAK--------K-TLKE-ALEAA
L8TMK9    AVSAARDGIVARGKAEPGDKTMVDAWAPAADAAKAAADTG-------GP-EVLS-VLVAA
M3BS32    ALRAGVDAVMKLGGAAPGDKTMIDALLPAVDALGE--------------------SFAAA
K2FJ00    AVEEALNGMKQRGKAEQGEKTMIDVWHPVADFLKNGQ---------------NV-NAGKL
G9QI21    ILKAGLEGIQKRGKAVPGEKTMVDVWVPVIEALESGN---------------------LT
M7NIQ8    YWVAFGKGIKERGQAEVGDKTMVDTLEPFFRTLEEEYASS--------R-DFSE-SFSRA
M7PNV0    VFSAGVESIKARGKADIGEKTMLDTLIPASQSLQQDAANG--------V-PLET-SLSHM
```

M5DZ01 IGETAVAGIQKRGKAEAGEKTMLDTIIPAVDALKESKKNG--------D-SVEE-ALQQC
N4WJ21 LFTHSLSAIKTRGKAEIGDKTMIDALQPAVEALQNTHV-E--------Q-DLLT-ALKQA
C9ABG8 IVEEGLTGIKMRGKAEFEDKTMIDVWQPVAEALK-KGELT--------K--------DTI
M9WI92 CLIQAVEGIKLRGKAQLNDKTILDVLIPVSELIQELIKTR--------L-DAKE-ILTKA
N6XD13 LILAALEGIQARGKAERGEKTMVDAWGPASDAAAKAASEG--------A-DEIA-VLEAA
N9VEI0 VYDAMLQEIMKRGKTEPGQKTMIDALYPALRAYREGLDAG--------R-RDEE-IIQDF
R4KG11 MALSAVKGVSERGKAKVGDRTMLDALSPAVDALVASLIEK--------S-ILTE-AFTKA
R4LHW9 ILRAGLDGVIARGKAEPGDKTMVDTLTPAVEALESALAAA--------R-PLPE-ALRAA
R2SJE3 LLSAAIAGIEKRGNSQPGQKTMLDLWVPAVEALK-TDSLT--------P--------EKI
R2Q950 IVNQGLQGIQARGKAVAEDKTMVDVWIPVAAALKNGE---------------------LT
R3TPV0 MIKNGIAAVQHRGKAQVGDKTMIDAFLPGLEVLETYQVEE--------D-PY-Q-FFEKF
R4UJY9 AYLAGIKAVKTLGKSDVGEGTMLDALIPAVKILESNLNLN--------NK----DLYKQM
R4T9Z9 ALRAALEGVQARGKAVGGDATMVDALIPAVSAEGASGDG------------VAA-ILAAA
S1P0N7 LMAQGLNGIQLRGKAEVGDKTMVDVWSPVVAALKEEN---------------------LT
R9KQ22 IMEAVIGGIKLRGKAERGEKTMLDALEPAMDALAKGIEAG--------D-SPED-CLAKM
S0FK13 MLNCAVEGIQKRGGAKLGDKTLLDALIPAAQSLRISANSG--------L-SISE-ASKLA
S0J6Y9 MMEAVIGGIKMRGKAERGEKTMLDALEPALEALTKGIEAG--------E-SPED-CLNHM
R9IRK6 MMEAVIGGIKMRGKAERGEKTMLDALEPAMEALTKGLEAG--------E-TPED-CLAHM
S3B602 ALRTGIEAVAQLSGAAAGDKTMLDALLPAVDALEDS---------------GGT-NFAKA
S2XG65 ILKAFEDAVAQRGKVTREEKTMYDVISRAREAVEN----------------GERVDLEKL
S2YZ61 ALRAGVDAVMTLGGAAPGDKTMIDALVPAVDALRD----------------G---GFAAA
S2W6B3 AFRAGVEGVVARGKAQAGDKTMLDALFPAADALDA----G--------S-DLGS-ALKKA
S3X4B0 ALQAGVEGVAERGRCMLGDKTLFDVLAPAVDAFAA---------------DFVD-PWGAA
S4AMF0 ALRTGVDAVAALGGAAPGDKTMLDALVPAIDALPT--------------------SFAAA
R7RQ76 MLLEALNGIKARGRASIGEKTMVDSLEPAINELKVSIDNK--------F-NELE-CLEKM
S4MG33 ALRAGVDAVRTLGGAAPGDKTMIDALVPAVDALGD--------------------SFTAA
S9SCC0 IIPAIRDGIAHRGKAVPGQKTMLDTWHPAAEAVIGGATAA------------------EV
S9S516 LLADVRDRMLARGGARLGDKTMVDMLDAVIRATDGLDAPE--------A------MAIAA
S5VBA9 ALRAGVDGVMALGGAAPGDKTMIDALAPAVDALPD--------------------GFAAA
T0TK32 LIYAGLEAIQKRGKAQVGEKTMVDIWSAFLKDLQTDS---------------------AS
S0LAM8 VLEAGALGIKKRGKAELEEKTMLDVWWPCVEELKENQ---------------------LT

```
T5KTF2          ALRAGLDGIVARGKAEAGDKTMFDAMAPAADAMDAALAAG--------S-SVAE-ATRAA
U1GE01          AFKADLDGIMTRGKAEPGDKTMVDALAPAVEALQTDAEAG--------R-TLQE-ALDHA
U1MTZ3          VLTAARDGIVARGKAESGDKTMVDAWTPAVEAASA---AG--------D-DVRA-ALAAA
U1N326          ALVEAATAIKARGKSEVGQKTMVDVWDPFLSDLNESDDFD--------A-----------
U2T4X1          LLTAARDGIVSRGKAEPGDKTMVDAWTPAVDAAAAAEASG--------A-SPA-------
U2TE92          MGKGAIAGVMERGKTKVGDKTMLDALAPAVDALEAASVAG--------K-SMKD-AWQDA
U2TIQ4          FYRAFADGLAKRGKTQLGDRTILDALDAGAVKAAEVDPAE--------G-FLA--VATAA
U2UUY7          VLLSMLDAITARGQAKEGDKTMIDSLAPAVKKYKECLDRK--------E-DAEL-LCEEV
U2N6F3          MLKDALDGIKMRGKAKAGEKTMIDALEPALEAINKGIEQG--------L-ESKE-ILKLA
U4VFA1          MLHAGLEGIQSIGSAKVGDKTLLDTLVPAIEAFDEATAAG--------K-SFAE-ALEAL
U5F6K8          ALENAVQGLMDLAGAKVGDKTLVDTIYPACQAYQAAINQD--------A-EFSQ-CLREM
U4K1X7          ILSEGLIGLQDISDAKVGDKCIMDTLVPAVEAMQKSVREE--------F-SFEG-SLSNM
U7VCP6          AAEAMVEGIKMRGKAQCGEKTMLDTIVPVVEVLKNNKEDS--------L-D--A-LLKKI
U7UHV1          AFAAAIEGVKMRGRAEKGDKTMLDALCPAYEALALSLADG--------E-ELAL-AVKKA
U7UNX5          ALKTMVEDMKKRGKTEVGSKTMIDALNPAVEELEKHLNSR--------EEDLVK-IFENV
U5MP41          ILQVSLDGIKMRGKSIEGEKTIIDTLSPSIEAGRKALDEN--------K-SQND-VLIAI
V2YGV3          IFAKALEELSDMSGAKVGDKTMMDALIPASQAIAAYEGGD--------E---AG-LFAEA
U5T5E1          MLEAGQQAVVNVGSAQIGDKTLLDTLSPATEVFANT---P--------G-TFEE-RLAAA
A0A2I5TCR4      LLATAVTAMTQRGKGELGQKSVLDIVNALANELTSISTFD--------E------MKHTA
U5VX81          VLRAALEGVVARGKAVAGDKTMYDALAPAVDALDAALAAG--------K-SLGD-ALAAA
V7IAM1          ILEAKLTAIMERGNAKPGYKTMIDPLYTAAVRMKAGMEAG--------M-DDTS-IISEM
A0A084A2U3      MIQEGAEGVMMRGKAEPGDKTMCDVWGPVVESLNQSLEQQ--------L-SLPQ-ALQQA
V5DRA3          VFMKAVEAVKQRGKAERGEKTMLDVLIPVAELLLQNAD-------------QPE-VLEQV
V6KYB2          ALRAGVEAVAQLGGAAEGDKTMLDALFPALAALDAALAGG--------E-ESAV-ALAAA
V6JJD1          ALRSGVDGVMALGGAAPGDKTMIDALVPAVDALAD--------------------GFAAA
V6Q9X7          ILEAGRDGIRKIGKSDRGEKTMLDMWFPVIEALKENK---------------------LT
V2Y558          ILEAMLDAIMSRGQAKPGFKTMIDSLYPAVEEYKNCLETG--------V-SDEE-LCDRV
G9WKH9          VFFTSLASLKKRGKAKVGDKTMVDALEPAVLALKEGSEDG--------L-DLTE-GLQKA
V7EEK5          ALQAAAQGITDRGRARPGDKTMLDAMQPAATAYAGALSQG--------D-SLPA-ALRAA
                                 . *
```

```
d3pnlb1     ssiaesaaqstitmq-arkgrasylger-sighqdpgatsvmfmmqmlalaake------
P76014      SSIAESAAQSTITMQ-ARKGRASYLGER-SIGHQDPGATSVMFMMQMLALAAKE------
K8E7T6      TEVIEYSVNATKDMK-ATKGRASYLGER-SIGHIDPGAASSGYLFETMMEAGVCNE----
Q1R0P1      RHGAQRGCDSTLELE-AKIGRAARLGER-SRGHLDAGAVSCCLLLTRLADSTERRLEKAA
A5G1S6      AAAAREGAEATAPLV-ARKGRASYLGER-AIGHVDPGATSSAMLLAALAEAAG-------
A7MG44      AQAAEQGAKATAQMGQARAGRSSYVGSDNLAGVEDPGAVAVAGVFKAITQGE--------
A7NKW3      VAAAEQGMKNTIPLV-AKKGRASYLAER-SAGHQDPGATSSYLMFKAMMDAWTK------
D9QAF6      AESAEQGAESTIPMV-ATKGRASYLGER-SVGHKDPGATSSALFLKAAADAAKECTAS--
Q1YEP1      ARAAEEGAEATKAMR-ASKGRAARLGDR-SLGHIDPGAASTALIVGVMATQLTG------
D1CI02      LPVAEEAARATAGLV-ARKGRSSRLGAR-GLGHPDPGAVSLTYILRAVEPALPA------
A0A0H3DEV8  ADAADTGAESTVDLV-PRKGRASYLGER-AVGHLDPGARSSALLLRAFAEAAR-------
A9KNP0      KQAAIDGAESTRDME-AVRGRATYQANK-GVGHLDPGAVTMSYQIATLMDFVKEHCE---
D7C870      SEAADKGALATVPML-ARKGRASYLGER-SIGHQDPGATSSALLFAALAEVASS------
D7DPP9      IQTAEQGMLSTKDLI-ATKGRAAGLGER-AIGHIDPGAKSCQLMIETVCHLLLKP-----
Q82NL7      RAAAEEGAVATTPLQ-ARKGRASYLGER-SIGHQDPGATSSALLIAALEEASVE------
F9USY1      QDVIDDAVASTEPMV-AKKGRASYLGER-SVGHLDPGAVSSGYLFTTLLDTEGIL-----
B9DU17      W----DAVKATEPLV-ATKGRASYVGDR-SKGHIDPGSYSSGLLFEAMLEAEVV------
F8A062      AQAAAQGAASTIPLV-ATKGRASYLGER-SAGHLDPGARSSELLLAAAVQVAP-------
Q5HKK0      Q----SFANETKDMV-ATKGRASYFNEA-SKGYIDPGAQSSVYILNAIIGGE--------
Q4L473      Q----QFATDTKEIE-ATKGRAAYFKKD-SIGYIDPGAQSMVYVLSALIGDDIE------
Q8R8K0      VNAAKEGMEYTKNIS-ARKGRASYLGER-SIGHIDPGATSSYLMIKALADVINLL-----
Q8XDH5      SSIAEFAAQSTITMQ-ARKGRASYLGER-SIGHQDPGATSVMFMMQMLALAAKE------
A6L9P2      AKAALDGAEATVPMK-ANFGRARNYGER-SIGFADSGATSWSCMFASFAEVL--------
Q6LL47      VANAEVAMKSTIPMQ-ARKGRASYLGER-SIGHQDPGATSTYLLFTALRNASAQ------
Q63NN9      SAAAERGKESTRWLQ-ARVGRASRLGER-SVGTLDAGAASCCLILCSLASSIGARLN---
Q65S46      VEKANISLQATIPLI-AKKGRASYLAER-SAGHQDPGATSTTYMLEALYNAVK-------
C7R307      AQGACAGADATVAMR-ASKGRSSYLGER-SVGHRDPGAQSTALILKAAAQTATNGEFHDS
A1SLY2      VDAAARAVEETRSLQ-SRRGRASWLQER-SIGLADPGATAFQRFVEAWSRVDGPSS----
B8D1L0      QVAAEKGKESTKDLV-AKVGRAARLGER-SRGVLDAGATSCNLILQAMANTIRDLLISE-
B7LGV9      SRIAESAAQSTITMQ-ARKGRASYLGER-SIGHQDPGATSVMFMMQMLALAAKE------
B7MKA1      SSIAESAAQSTITMQ-ARKGRASYLGER-SIGHQDPGATSVMFMMQMLALAAKE------
```

A0JRA4	AEAAEAGAVATDPLI-ARKGRASYLGER-SAGHRDPGAVSTALILRAAAGAAG-------
A0KQ90	VAAAAAGTEATIQMQ-ARKGRASYLGER-SIGHQDAGATSTLLLLTALRDRARL------
A0QU92	ATAAEKGRDATESMI-ARKGRASYLGQR-SVGHIDPGATSAAMLIAAAATAAGSTS----
C7LYL5	ASVASERAESTIPLV-ARKGRASYLGER-SAGHEDPGAASMALFFACLGEVS--------
Q9CKH0	AKAAEEGAENTRHQV-AVFGRAKSLGER-SLGYVDPGAVSMSFIIKYMAEFVNQ------
A0A3N4B7H8	KHAASMGRDSTLDLV-AKIGRASRLGER-SRGVLDAGATSCCLILTQLADSVERRLVP--
Q8YA19	VDAMHAGAQSTIPLI-AKKGRALRLGER-AIGHLDPGSESSWMLMNIILENLKKAV----
Q8DY42	EDRIDSLVDATKGMK-ATKGRASYVGER-SVGHIDPGSFSSGLLFKALLEVGGV------
Q8ENK9	EETAKRAMNDTKELK-ATKGRASYFKEK-SIGHIDPGSASSYYIFSALATSIKGA-----
C7MWZ8	ADAADEGANSTKPLV-ARKGRASYLGQR-SIGHLDPGARSTALLLRAFAEAAR-------
D1AJM4	LEAAEKGMLSTKDIQ-AKRGRASYLGER-SIGTVDPGARSSYLILKTIYNQI--------
D5UD19	AQGAHTGAIATVPLV-ATKGRASYLGER-SAGHLDPGARSSELILVAAAQAAAAAD----
C7Q5T1	AAAAETGMQDTTPLQ-ARKGRASYLGAR-SIGHQDPGATSTALVFRALAEVLQS------
D7GCR5	STAAQQGRDATVDLV-ARRGRASYLGER-SKGHLDPGAASLTMLIESAARTLS-------
A0A384LQB2	SSIAESAAQSTITMQ-ARKGRASYLGER-SIGHQDPGATSVMFMMQMLALAAKE------
Q6MTW9	VQLAYLKVEETKNII-AKKGRASYLGQR-SVGHIDPGSYSSYLILKCISENI--------
B2TZU4	SSIAESAAQSTITMQ-ARKGRASYLGER-SIGHQDPGATSVMFMMQMLALAAKE------
C3KP63	SEQAERAASLTFGMR-AMRGRAAFLGDR-SIGHVDPGAKSCALLTAAICRFLEEQCPA--
A6VPA2	VKKAAEESVAATIPLV-AKKGRASYLGER-SVGHADPGATSSMLIIKALANAVQGA-----
A7ZKX1	SRIAESAAQSTITMQ-ARKGRASYLGER-SIGHQDPGATSVMFMMQMLALAAKE------
A0A7I6GZ14	SSIAESAAQSTITMQ-ARKGRASYLGER-SIGHQDPGATSVMFMMQMLALAAKE------
Q5WJG3	EASAREGMEATKDMV-VKRGRGAILGEQ-SVGHIDPGAVSSYYLFKTLCNTMKAGE----
A9H2V8	AEAARQGAEATIPMI-ARKGRASYLGER-SAGHEDPGAASSALLLTALADTAG-------
B2J6P9	VAIAQKGLQETIPMQ-AKKGRASYLGER-SIGHQDPGGTSAYLMLKSLLEVLENSQTTDH
B2JWR9	PEVAEAGMLATRDML-ATKGRASFLGER-SRGHIDPGARSSQLMIDAVCARLAQDKE---
K5Y1C8	AKAALAGAASTVEMK-ANFGRARNYGER-SIGYADSGATSWSCMFESFAQAL--------
Q9K7G5	Q----QFVSETKEMK-ATKGRASYLGER-SLGHLDPGAVSSGYLFEAMIDGGILS-----
G2SM22	NADIEAAVESTKDMV-AKKGRASYLGER-SVGHLDPGAVSSGYLFEALLEAEESL-----
Q6AFI5	ADAAEKGAEATEPLV-ARKGRASYLGGR-STGHRDPGAQSTALILRAASETAA-------
B0S302	SVDCKLACDNTANMA-ANKGKARHNAIY-SVGVIDPGAYSLFLFFDTLYKYVLKKRRTK-
B8I2J6	KSEAEKGKESTRDMV-AKIGRAARLGER-SRGVLDAGATSCNLILKSMADAIIELL----

```
C8WGF7        AEAAEEGARASEGFA-SKFGRARSYGDR-TIGTPDAGAVSTSLFLRGLANGASLPRS---
D2SH30        AEAAAQGRDATEPLV-ARKGRASYLGER-SAGHVDPGAASATLLLEAAATALAGRG----
G2NB77        GDAALAGALATVPLR-ARKGRASYLGER-SIGHEDPGAASAALLVAALAEAAGTGDGGRA
A0A0H2V747    SSIAESAAQSTITMQ-ARKGRASYLGER-SIGHQDPGATSVMFMMQMLALAAKE------
Q893Q0        KDEALKGVNYTKDII-ARKGRASYLGER-SIGHQDPGATSSYLILNEIYKYVCGK-----
Q835L6        EEKIEQFAEATKDWV-AKKGRASYLGER-AIGHIDPGAASSALLFEELVKVINQ------
Q7NC54        QKILDENFNKSLLLK-SKVGRASYHGKR-SIGIYDPGTVFIYIILEGIIKIYDQ------
A3MYF4        VEKAENFAKATVPMT-AKKGRASYLGER-SVGHADPGATSSYYLIKALAEAVK-------
C9YYH4        RAAAEAGAAATTPLQ-ARKGRASYLGER-SIGHQDPGATSAALLIAALAATAGTPEATDV
C4LG95        ADAANKGAEATVDMT-ATKGRASYLGER-SVGHKDPGATSSAYFLAEASKAVDA------
D7DWU8        VSAAEQGLQETIPML-AKKGRASYLGDR-SVGHQDPGGTSAYFMLRSLLKVVDSQKKGGR
A6TVH6        KQGAIDGMNSTKEME-AVKGRAYYQAQK-GVGHIDPGAVTMCYQIEVLVDYLCK------
D9TSY8        VNASYSGVEMTKKIA-ARRGRASYLGDR-SIGHQDPGATSAYLMIKTVANYVDKLQQ---
Q03BZ6        D----KLVASTEPMI-AKRGRASYLGEK-SVGHIDPGAASTGELLKAYLDATA-------
D9QZW4        AEAAWEGFEQTTTLI-AVHGRAATRGEA-SRSLKDPGAAVAALIMKAVADQ---------
B1YE66        --TLDQALADTKERV-ATKGRASYFGDA-TVGVQDPGALSSSLLLRSIVEVLS-------
D4GL25        CQQAESAAQSTITMQ-ARKGRASYLGER-SIGHQDPGATSVMFMMQMLAAAARE------
D5E5M2        YHAAKDENEFIKNIA-ARSGRASYLGER-SIGVNDPGATTIYLIFELLNKEVK-------
Q2RUQ3        TLAAERGRDATRDMI-AKVGRAARLGER-SRGALDAGASSCCLILGTLACGVKKRLA---
Q2JZR2        EQAARQGCEATKDMT-AAKGRSSRLGER-VIGHIDPGAASAAIVIASIAKFVEQHDSCRI
I2BC43        CQVAGQAAQATITMQ-ARKGRASYLGER-SIGHQDPGATSVLLMVQCLAAAANA------
Q9FC12        RTAAVEGAEATTPLQ-ARKGRASYLGER-SIGHQDPGATSSALLFVALEETAGE------
Q92WN5        TAAAENGASATRSMV-ATKGRAARVGER-SLGHMDPGAASAVLIIRAMAQTLREG-----
Q12FM9        KEEAELNMLATRDMI-ATKGRAHFLGER-ALGHIDPGAKTSQVALFAVCDMLTIGAAS--
A4YWS0        PAVAAEAADATVPMK-AIRGRASFLGER-SIGHMDPGARSSSLLVQAICSVFNEV-----
A9WUM9        AAAAEDGGRDTESLL-ARKGRASYLGER-SVGHRDPGAQSSALILRDAANTARTAL----
A9W9R1        AAAAEAGAKATIPML-AMKGRASYLGER-SIGHQDPGATSTAYLLRALAMTCSRNEEEA-
D1C9M9        AAAARTGMEATIPML-ATKGRASYLGER-SIGHQDPGATSSYLIVQAIADVVRENSG---
D2B9Y6        ASAALAGSEATVPMQ-ARKGRASYLGPR-SVGHEDPGAASTALILAALRTVVTR------
D1B6Y5        VSAAEEGMKSTIPML-AKKGRASYLGER-SVGHQDPGATSAYLLLSCLKEAVVA------
A0A140NCI1    SSIAESAAQSTITMQ-ARKGRASYLGER-SIGHQDPGATSVMFMMQMLALAAKE------
```

```
C3PGU3        ADAAAAGAEATIPMV-ATKGRASYLGER-SAGHKDPGATSSALFIAAAADATETLKEEA-
A9G640        AEAAQAGALATVPLI-ARKGRASYLGER-SAGHQDPGATSCSLLLRSLAATAGV------
A3CK11        G----SLVEATKDLK-ATKGRASYVGER-SIDHIDPGSASSGLLFEALLETEAG------
B0K761        ANAAKEGMEYTKNIS-ARKGRASYLGER-SIGHLDPGATSAYLMIKSFSDVVNLS-----
Q72DE9        VDAAGEGMRATIPMQ-ARKGRASYLGER-SIGHQDPGATSTVFMLTALRDVVRG------
Q9CIV7        KDNLEKVVKASAGLL-ATKGRASYLGER-SIGHIDPGTQSSAYLFETLLEVVA-------
Q6NEE0        AAAAAQGAADTEPMV-ATKGRASYLGER-SVGHRDPGATSSAYFLQAAADAASEGTA---
C6WHH1        AAAAEGGARATVPLV-ARKGRASYLGER-SAGHMDPGARSTALLLGALARVAAG------
C8XFT9        ANDAAMGAAATIPLQ-ARKGRASYLGAR-SIGHQDPGATSTAYLLEALAAVAEKAP----
D7AWT1        AEAAREGADGTSELL-GRRGRTSYVGEA-ALGSPDPGAVTVALFYRAAQGVVGV------
D1BZN2        AAAADAGSTATIPLV-ATKGRASYLGER-SAGHEDPGARSTALLLAAAVAAAEEQ-----
A6X6C1        LEVAEKGTDSTRSMV-ATRGRAARLGER-SLGHVDPGAASAIIILRAMRDSFTEAN----
E1R8P9        VEAAEAGLASTIEMV-AKKGRASYLGER-SKGHQDPGATSSYYLLKAALETWK-------
E2Q762        AAAAAEGALATVPMR-ARKGRASYLGER-SMGHQDPGATSSALLVAALAEAERTEGEAP-
B7KD17        VATAEQAMKNTIPLM-AKKGRASYLGQR-SIGHQDPGATSCYLILKTLGETVEG------
A5N619        RNAAFIGVKSTESII-CKNGKASYLGEK-SIGYPDPGATSSYLMLNAIYEKVKELCAVEK
Q1H2B4        QQAAEQGMLSTKDMV-ATKGRAAFLGER-AIGHIDPGARSSQIIIHTIAELAKQQAV---
E3J2C4        AEAARGGALATEALR-ARRGRASYVGDV-ARGVLDPGALTVALFFDSCHG----------
Q87SQ7        VELAETNAVATIEML-AKKGRASYLGER-SIGHQDPGATSSLLMIKALQQAIAGN-----
Q881Q7        KNAAAQGRDSTKDLM-AKIGRASRLGER-SVGVLDAGAVSCCLILTQLADSVQPRLKAG-
Q8RHZ7        IEVAKNSMEATKDII-ATKGRASYLGER-SVGHIDPGAMSSYIMIKTVCENI--------
Q8EW70        DAKAFEGKESTQNII-ATRGRASYLKDR-SLGSLDPGSVSSYLIINTIYKSLSGE-----
Q89PB0        RTTAAEAVERTAPMQ-ATKGRASFLGPR-SVGHVDPGARSSCVLVQAVCASLEGQQ----
C6BY70        LPVGEKALADTIPLQ-ARKGRASYLGER-SIGHQDPGATSSYYMLETLKEVLS-------
H0TUV4        ATVAREAADATVPMK-AIRGRASFLGER-SIGHMDPGARSASLLVQAVCNVFNEA-----
Q313B6        VAAAEKGLEATVPMQ-ARKGRASYLGER-SIGHKDPGGASSCMMLGALRQSIGG------
Q32H07        SSIAESAAQSTITMQ-ARKGRASYLGER-SIGHQDPGATSVMFMMQMLALAAKE------
C6WWI2        MQTAEEGMLATKALI-ATKGRAASLGER-AIGHIDPGAKSCQLMINAVCNQVLASQL---
C6XCW2        KAEAEKGMLATRDML-ATKGRAAGLGER-AIGHIDPGAKSCQVMIHAVCDMLLGQAH---
A0A094YZF9    RATAESCMKATEILE-AKKGRAAYLGKR-SVGHIDAGAYSSYLLFLSLAETLAKRGE---
F4XFL5        AQAAQEGAKASEQFV-SKFGRARSYKEQ-TIGTPDAGAVSTALFFRGLAQGI--------
```

```
B5HKC0      REAAEQGASDTVPLQ-ARKGRASYLGER-SIGHQDPGATSSALLLAALADTAEGAA----
I2F888      ATKAEEGAESTKTIV-ATKGRARYLGVR-SIGAYDAGARAVAVILSDIQKNFFNKTN---
I6RZA4      E----EAKEHTKDLV-AKKGRASYLGER-AIGHIDPGAASSAIFFETLLTNIES------
A3YTX8      RAVALEAAEATKPLR-AQRGRASFLGER-SIGHVDPGARSCALLVAAVTDVLEAPESAP-
J0MWJ2      AAAADAGRAATESMR-AMKGRASYLGER-SIGHIDPGASSTALIVRALADVVDGCAS---
E5BF30      KTVAKQGMESTKDII-ATKGRASYVGER-SLGHIDPGAASSYMMIKVICEEIK-------
F3NT86      GAAAEEGAVGTTPLR-ARKGRASYLGER-SIGHQDPGATSSALLIGALKEARGE------
E0QM99      GKAARAGAEATTPLL-ARKGRASYLGER-SVGHQDPGATSSAYLFETLAKVVS-------
C4IDX1      IKKAKEGMENTKNII-ATKGRASYLKER-SLGHKDAGATSMYLILNTIVEKSI-------
I9GRJ3      AEAALKGAAETVNMK-ANFGRARNYGER-SIGYADSGASSWSCMFDAFAKALL-------
H0HYA5      ADIADQAAEATKPLK-AIRGRASVLGDR-SIGHMDPGARSSALITRAIAGTLEPRV----
F3B1P8      KKAAIDGANETAKME-AVKGRATYQADK-GVGHLDPGAVTMSYQLVCLMEAVEEKL----
G5K1Y5      KDAVEEMVLATKAIQ-ATKGRASYVGER-SVGHIDPGSFSSGLLFEAMIEAEVI------
F3PPM2      AEAAIAGAESTLEMK-ANFGRARNYGER-SIGHIDSGAASWSCMFASFAEALN-------
F5T169      KTAATQGMQSTKDML-ATKGRASFLGER-ARGHIDAGARTSQLILCVIADELSKI-----
H3NBU3      TDDLAQLAEDTKELK-AKKGRASYLGDR-SVGHIDPGAMSSVYFFQALVEAEAVK-----
G9PIG6      ARAAADGARDTEPLQ-ARRGRASFLGER-SCGHRDPGAQSSALIFQAALDAARACSLEPI
A6EMJ5      VNEAEKGMKSTVNMI-ARKGRASYLGER-SVGHQDPGATSSYFILKALKESIN-------
W0IWI1      TEAADRGRDSTKDLV-AKLGRAARLGER-SRGVLDAGATSCALILKTLARAFGANP----
C7HTR7      LKIAQEKMEKTKEIK-ARKGRAAYLGDR-SIGHIDPGAYSSYLCIKSIVGDLND------
H0QJK9      AEAAEAGAVATDPLV-ARKGRASYLGER-SAGHRDPGAASSALILRAAAGAAA-------
A4AIN0      LAAAEKGRDSTEPLV-ARKGRASYLGER-STGHLDPGATSSTLLFQALADTVTS------
F0Z4L2      KKAAVRGRDSTKELI-AKFGRSSRLGER-SRGVLDAGACSCCIILTAMADGILELL----
K0YPL4      SEAAHKGALATEPLQ-ATKGRASYLGER-SKGHLDPGAVSSAIILAKALEAAEVE-----
I8UJC4      T----QALQTCQALS-AKKGRAAYFGDN-AIGKQDPGAQSSALLLRPFAEVM--------
C8PCR8      TEKIEAAVLATKDMI-AKKGRASYLGER-SINHIDPGSQSSGYLFESLLEVINNK-----
C2L0R3      ALAAEKGMEYTKTIK-ARFGRAKYFGDK-AIGLQDAGATSVYIIFQAMSDFLKGEKN---
D9X7S5      RTAAEEGAVATTPLL-ARKGRASYLGER-SIGHQDPGATSAALLVAGLVEASGE------
C6PVN6      KDSALKGVLHTKEII-AKKGRASYLGER-SIGHQDPGATSSYLMLNTIYECIKEISQK--
G2GMN3      RAAAEEGAVATTPLR-ARKGRASYLGER-SVGHQDPGATSAALLIAALGEAGDE------
D0GNQ9      YDKAAKGSENTKEMK-AVHGRAAYHDEK-TLGHIDGGAYVGKLIFEGIYNFYKTK-----
```

```
G6FMN8    VAAAEKGMQDTTPMQ-AKKGRASYLGER-SVGHQDPGATSVYLMLQSLLDVIS-------
E0NGR7    AEDVDRAVESTKPMI-AKKGRASYLGER-SVGHLDPGAVSTGYLLKTLLAVEAK------
C6JM61    ELKGKAGMESTKNIA-ATKGRASYLGER-SIGHLDPGSVSSYLIIKTINDELKG------
D6U6Y4    SSAAEAGMKSTIPLL-ATKGRASYLGER-SIGHQDPGATSSTLMLQALAETYR-------
G3ITN9    ARTAIAGMESTRDML-ATKGRASFLEER-SRGHIDAGAKTSQLMICALVDVLAKHLTAPA
E6MJW9    EDAAWKGVAYTKTII-ATKGRASYLGER-SIGHQDPGATSITYMVQAARAVAEAK-----
C0W3K3    ARAAQEGRGATAAMR-ATKGRASYLGQR-SVGHIDPGAASTALVLQALAQAETATSQEEQ
C8NCE6    ARTAQEQAEATIPLV-AKKGRASYLGER-SAGHKDPGAASSALLLQCLAEAV--------
E8LGV2    GKAAAEGAEKTADMV-ARFGRAKNVGER-SLGTKDPGATSISILLTAMAAAI--------
H5X503    ADAADEGARSTVPLV-ARKGRASYLGER-SVGHLDPGARSTALLLRAFADAAR-------
A6DNZ7    HQAALEGIEVTKTLV-ANKGRSRYAGER-GLGHQDAGATSVTLIIKCFAEQATKEA----
B1FVY6    PQVAEQNMLATRDML-ATKGRASFLGER-SRGHIDPGARSSQTMITAVCAHLARNPV---
F9QEG7    LKVAYDSKELVKNKP-ATKGRASYLGER-TIGIVDPGSQSMYYILETLVENYND------
I1D5G2    ADAAEEGANSTKPLV-ARKGRASYLGER-SIGHLDPGARSTALLLRAFAEVAQ-------
H8GRD8    VRTAEAGVEATRNML-ATKGRASFLGER-TLGHLDAGAKTAQLMISAIAEALASSAAPT-
E3CX22    VAAAEQGMTSTIPLV-ARKGRASYLGER-SAGHQDPGATSTYLLLNCLRAAALGES----
G7M3Z5    KEAAYKGVEHTKEII-AKKGRASYVGER-SIGHQDAGATSSAIMLETIYEAL--------
B1CB33    LQASMDGVEYTKTIK-ATKGRASYIGDR-SIGHQDPGATSFTYMMECINDII--------
G4CVL8    AEAANEGRDATIEMV-ARRGRASYLGER-SRGHQDPGATSMAMLVGSAARCLSGAES---
F9EGT1    AAAADAGRTATGPML-ATKGRASYLGER-SVGHIDPGAASTALLLRALAEVMGGRAS---
I8TY96    CEAAQQGVEFTKTII-ATKGRASYLGER-SRGHQDPGATSSYIMLKALLEHLRRQ-----
G5IBQ9    KEAAVRGAKESEQYI-SKFGRAKSYKEQ-TIGTPDAGAVSTSLLFVGLCDGFRES-----
G5KIC1    IEKIKNVVEATKDLK-ATKGRASYVGER-SIGHIDPGSYSSGLLFEAMIEAEVD------
F9ELQ8    IEVAKNSMEATKDII-ATKGRASYLGER-SVGHIDPGAMSSYLMIKTVCENI--------
A8RYY3    VRAAWAGAEHTKDLV-ATKGRASYVGER-GLGHQDPGATSYSYMLEVIAGLV--------
G8TSR2    ARHARIWAEATIPLT-AKKGRAAYLGPR-SQGHLDPGALSSAIWWECWLEEGVAR-----
G8S8S8    AEAAAAGAAATVPLR-ARKGRASYLGER-SIGHQDPGATSTSLIFQALAAVS--------
G8PBG1    SKLIDESVEKTRDMQ-ATKGRASYVGER-SIGHIDPGSMSSGYLLKTLLKVGE-------
F7KPB8    QECAEKGKESTKEMV-AMYGRAARLGER-SRGVLDAGATSCCIILKAMADGMKKVLE---
G8QU73    SLAAQKGYEATVGML-AIHGRAATRGEA-SRQLLDPGAAVAALLMKAFAVALQ-------
D4E0S0    AECAQQAVEATIAMQ-ARKGRASYLGER-SIGHQDPGATSVMLMVKTLAEIAA-------
```

```
F1T4X2      CDSIKALVDATAPLR-ATKGRASYTGER-SCGHIDPGAASSGMLFCALLEQGV-------
I4BYG6      LDMAEKGMKDTIPLV-ARKGRASYLGER-SAGHQDPGATSSYLILKAICEALGV------
J9Z1A9      VQVAEKGMLSTKDLL-ATKGRASFLGER-AKGHIDPGARSSQLMIKTVCESVIQK-----
F9ZQA6      AEAARQGLAATIPLI-ARKGRASYLGER-SRGHADPGAASASLILECLAGDDASTS----
I1A7E4      D----QLVKKTENMV-ATKGRASYLKER-SLGTIDPGSFTSSVILKHLIKEL--------
C9MZ82      SDAATKGSEATKNMM-AVHGRAAYHGEK-TIGHVDGGSYVGKLIFEGIYNYYKEKAEKLY
K0K2T4      AEAAEQGAESTVPLV-ARKGRASYLGER-SAGHLDPGARSTSLLLRSFAGGAR-------
Q49VJ6      Q----SYADMTKDIE-ATKGRASYFKED-SKGHVDPGAQSSVYILNALIGDES-------
Q5FJ65      EDKIKDAVEATKDMI-AKKGRASYLGER-SKGHIDPGSESSGYLFETLLETEGLL-----
I3D7P6      VKKAKISLEATIPLV-AKKGRASYLAER-SVGYQDPGATSTMYMLEALYQAVK-------
I4F4L6      AAAAGAGRDATVPLV-ARKGRASYLGER-SAGHQDPGATSTALLLDAAVTALSRGEG---
Q1WRP5      QADIDAAVESTKDMV-AKKGRASYLGER-SVGHLDPGAVSSGYLFTALLEAGVM------
D7A0Q6      AECAQSAARATEMML-ARRGRASYLGER-SVGHEDPGASSCALLTTTICLYLKEHAAP--
Q1AX45      AEAAGEGAEATVPLT-ARKGRASYLGAR-AAGHKDPGAASTAYLLAAAARAVEGS-----
A4FMZ5      AEAAHVGAQATEPLV-ARKGRASYLGER-SAGHIDPGARSTALLLSAFSEAARGGR----
D6ZIF3      ADAAAKGAEDTVSMS-AAKGRASLLGER-SIGHKDPGATSSALILAEAAKAAAH------
Q60CU3      SRAAEAGMESTRDML-ATKGRASFLGER-AIGHVDAGARSSQLMISAIAEVVAESAW---
A8LNW4      AEAVDRALEDFRDKP-ATVGRARIFGEK-SIGLDDPGMLAMQSVVRAAVDK---------
F2N7D3      IALAEEKCESTRDML-AKKGRASYLKER-SIGHLDPGARSCSLLIEVMCQQLEKKEAAHA
F2R6Z8      RAAAEEGALATVPLR-ARKGRASYLGER-SVGHQDPGATSSALLFAALDETAGAESPEVP
E3DLJ7      KASAEEGMKATIEMQ-ATKGRASYLGER-SIGHQDPGATSTYLIIKTVVDELY-------
E3G7K1      TDVAEAAAHATIPMQ-ARKGRASYLGER-SIGHQDPGATSVLFMMQMLTAAARE------
E3GPD1      EAAAWDGVEYTKTII-ATKGRASYLGER-SIGHQDPGATSITYLIQAAKEAGEQAGA---
E3H7Q4      VIAARDGVEYTKTIK-ATKGRASYLGDR-SIGHADPGATSTKLILEAILEVL--------
K2QR18      ETVAEEALEATKSMV-AQHGKAAAFQEK-TVGLQDAGATVGFFLIRRMSQFAANH-----
H6LKL8      EQAAWEGVEYTKTII-ATKGRASYLGER-SLGHQDPGATSMAYVFQAAKEVALKAGN---
J2ZU17      T----DAVQATEPMK-ATKGRASYVGDN-SIGHLDPGSVSSGYLFSELLKAGVTA-----
J2VAZ1      GKAAETGAEATKAMI-ASKGRSARLGER-AIGHIDPGAASAAILIRAMGRGLG-------
K2N9I0      QRAANDALDQMRPMP-CRIGRARMFAER-SIGSDDPGMLAVAKLLD--------------
I0JQA6      LDTAQSTMESTKDLV-ATKGRASYLKER-SKGHIDPGAMSSYYVFEALADCIKGDE----
E4RNI0      KAKAEEGMKATIPMQ-ATKGRASYLGER-SIGHQDPGATSSYLMIKTVVDELYKYKD---
```

```
F4CPQ6          AAAAAAGRDATIPLV-ARKGRASYLGER-SAGHQDPGATSVTMLVAAAADALA-------
A0A0H3GYP2      CEVAERAAHATITMQ-ARKGRASYLGER-SIGHQDPGATSVLFMVQMLAAAAKE------
F8JV61          RSAADEGAAATVPML-ARKGRASYLGER-SVGHQDPGATSAALLVAALASAAGAATGDAR
I0I650          VAAAEAGMRATIPMQ-ARKGRASYLGER-SIGHQDPGATSTHLILLALADALADKDAKSA
I0HCV6          AVAAREGARATAGLL-ARKGRASYLGER-SVGHQDPGATSAALLFRALSEQTGNSG----
F5X6F7          IDRINEYVQATSPLK-ATKGRASYVGER-SIGHIDPGAFSSGLLFKALIEAEE-------
F5XQ38          AAAAAQGRDFVTPLV-ARKGRASYLGQR-SADHQDPGATSAVLLIQAAATALGSPAN---
C5BWZ3          ASAAQEGAVATEPLV-ALKGRASYLGER-SAGHRDPGAESTALLLAAAARAAQEAA----
D2PZT9          AEAAAQGRDATVPLV-ARKGRASYLGER-SAGHQDPGATSTTLLLESAARTLG-------
K4RFM8          RAAAEEGALATIPLQ-ARKGRASYLGER-SIGHQDPGATSAALLVAALAEANGE------
A8AFT8          SAQAEAAAQSTITMQ-ARKGRASYLGER-SIGHQDPGATSVMFMMQMLAAAAKE------
B7UQ88          SSIAESAAQSTITMQ-ARKGRASYLGER-SIGHQDPGATSVMFMMQMLALAAKE------
E0U9C1          VTTAETEMKNTIPLV-AKKGRASYLGPR-SAGHQDPGATSCYLILKALLETVKDDS----
Q73K63          EKIAKEGSDSTRNMK-SVHGRAAYYAEK-SIGILDGGSYAGYLVFKALNNYLKDK-----
A0A0H2YY24      SSIAESAAQSTITMQ-ARKGRASYLGER-SIGHQDPGATSVMFMMQMLALAAKE------
A0Q3F2          KDAAFKGVEHTKEIV-AKKGRASYLGER-SIGHQDPGATSCALILESIYEEVKKLQ----
D3F3G8          ASAADAGADATIPLL-ARKGRASYLGER-SVGHLDPGAVSSALLLRTAADAWGESTE---
D3R2L5          KQVAADGAEATKGME-AVKGRASYQKNK-GVGHLDPGAVTMSYQIGVLCDVISKEIKAE-
F4BL99          SSFIDETVEKTIDMK-ATKGRASYLGAR-SIGHIDPGAMSSSYLFKTMMKAGVYDE----
F4F7H7          ATAAEQGRDATAAMV-ARMGRGSYLGDR-SRGHIDAGATSMAMLLRCLHASAEEVTS---
G4T0K8          CDTANQGMEATRDML-ATKGRASFLGER-TRGHIDAGAKTAELMICAIATVLAK------
F4H6I8          ARAATDGAQATIPLV-ATKGRASYLGER-SAGHLDPGARSTELLLAAAAQAAA-------
K6UNQ1          ADAAAQGRDATTPLV-ARKGRASYLGER-SAGHQDPGATSTAMLLAAAAKAFSA------
B0TAR9          TAAARQGVEFTKTIV-ATKGRASYLGER-SKGHQDPGATSAAIMLAAITDFCRKAP----
A0A0H3EI07      SSIAESAAQSTITMQ-ARKGRASYLGER-SIGHQDPGATSVMFMMQMLALAAKE------
D6EB63          AQAALTGAERSKDFP-SKFGRAKSYGEK-TIGTPDAGAVSMACFFKGLAQA---------
Q2G0E9          Q----QLADNTKDMV-ATKGRAAYFGEE-SKGYIDPGAQSMVYILNALIGDEDNA-----
F9ZY89          CAAAEAGVESTRDML-ATKGRASFLGER-SKGHIDAGAKTAQLMIEAIARQIRSAA----
E6SH85          ARAARQGSDATIPMV-ATKGRASYLGER-SRGHRDPGSLSATLMMEAAAETLPGGTEA--
F7V0N8          AAAAEAGAKASEGFA-SKFGRARSYGEQ-TIGTPDAGAVSTSLFFCGLADGARS------
D4MT14          TRAAEDGVEYTKTII-ATKGRASYVGER-SIGHQDPGATSFTFMMETVEEAL--------
```

A0A8B1NT48 AAASEKGALATVPML-ARKGRASYLGER-SIGHQDPGATSSALLLAALAEVAK-------
A0A0H3MXE5 TEIIHKAVQGTKDIA-ATKGRASYVGER-SIGHIDPGSFSSGLLFEAMLEAGL-------
I1YIS9 QQAAEQGMLSTKDML-PTKGRASFLGER-ARGHIDAGARTSQLILSAIADEVIQNQ----
F5YG98 KTAAASGWESTKDME-ARFGRSSRLGER-SRGVYDAGATSCKIILKAMADGILEVAR---
F6EM32 VTAANAGRDATIPML-ARKGRASYLGER-SVGHQDPGATSAALLITAAARAFG-------
F6FRU1 AEAADAGATSTIPLV-ATKGRASYLGPR-SAGHEDPGARSTALLLGAALAAALTDEVPAA
K9ETT1 D----QIVEKTKEMK-AHKGRASYLGDR-SIGHIDPGAMSSKYLFEAFIKGGVFD-----
M2XUI3 EAAAVEGARSSAESL-ARRGRASYVGEA-SQGVIDPGAAAMALVLGAARAAADAGRGEQH
K9CFB7 LSAGREGAEYTKTIV-ARRGRAALIGER-SIGIEDPGAMSSLIMFRALCSYLRG------
G0VNU3 AAAAEKGVEYTKTII-ATKGRASYLGER-SLGHQDPGATSSLYLLQVLAEMA--------
D7UV97 DDVVEAALQKTKDIK-ATKGRASYLGER-SIGHLDPGAYSSALLFHTMLETEVD------
C8RYK0 RMAAEQARETTVTMI-AARGRASRLKER-SLGHLDPGGASAAIILAAIADTLASAA----
K8WHA8 VDAAKQGVESTIPMQ-ARKGRASYLGER-SIGHADPGGMSVFLMLETLQKAI--------
E6LEU6 Q----EYVQASADLK-ATKGRASYVGDR-SIGHIDPGTCSSGYLFEALLEASK-------
C0W168 ALAARAGAEATLEMK-ATKGRASYLGER-SIGHLDPGSVSSAIIFEQAVKAAKAGA----
M4U7Q4 CQRAEEAVTRTIEMQ-AKKGIASYLGAR-SVGYQDPGATSTLLILKALKKAQLES-----
K9RKI5 VAAAEQGMKETIPMV-AKKGRASYLGER-SKGHQDPGATSVYLMLKSLQESLI-------
K9TAS4 VVAAEQGMKDTIPML-AKKGRASYLGER-SIGHQDPGATSSYLMLKSLLAALECS-----
K9U7Y7 VIVAEQAMKDTIPML-AKKGRASYLGDR-SIGHQDPGATSTYLMLKSLLAALES------
L1L3V1 RSAAEEGALATTPLQ-ARKGRASYLGER-SIGHQDPGATSAALLIAALADTAQAPAASEA
L0RXD0 Q----YLVAQTKDMM-ATKGRASYLKER-SIGTIDPGSFSTGIILKNFLKEL--------
K9WJ60 VSAAEAGMKSTIPLV-AKKGRASYLGER-SSGHQDPGATSTYFILKALLDTVNTH-----
K9ZQU8 VAAAEQGLKATIPML-AKKGRASYLGDR-SIGHQDPGGTSAYWMLRSLLEVLKGSNTIRN
K9XI13 TTAAEQGMQATIPML-AKKGRASYLGDR-SINHQDPGATSVYLMLQTLSEVLDMTSD---
K9VA65 VVAAEQGMKDTIPMI-AKKGRASYLGER-SIGNQDPGATSCYIMLKCLCDALQK------
L5NDP0 KEAAETAMEATESLQ-ATKGRASYLKER-SVGHLDPGAVSSFYLFEALASVEGAE-----
L0EJ60 AEAAKQGAERTASMQ-SRHGRASRYLER-SIGHQDPGATVGALIVQGFADYVNKTSSAQ-
F4LS03 IDTARKGAESTIDMI-AKKGRAMRLGER-SIGHLDPGAASSTMILEVFADCFPC------
L7FGQ5 RTAAEEGALATVPLQ-ARKGRASYLGER-SIGHQDPGATSAALLIATLAETAGE------
L0KF84 ANVADAAAEATVPMK-ALRGRASFLGDR-SIGHMDAGARSTALLVRTLAQIIGGKA----
F4EWM0 AVAAEKGAERTKDLV-ATKGRASYLGER-SLGHRDPGAVSSSFMLRAMADAM--------

```
L8TMK9    AEAAEAGAVATDPLV-ARKGRASYLGER-SAGHRDPGAASSALILRAAVGAAA-------
M3BS32    RDAAREGAVATTPML-ARKGRASYLGDR-SIGHQDPGATSAALLVSALLEAGPGDGEAGG
K2FJ00    VEVAEQAMEGTKDTV-ATKGRAAYLQDR-SKGHIDPGSTSSFYLFQALADVWKEGE----
G9QI21    KETIQEAVNQTKDMK-ATKGRASYLGER-SIGHLDPGAVSSGYFFEAMLEGGILNE----
M7NIQ8    VDAAKAGMESTKDLL-SKRGRSSRLGDR-SIGAQDPGATSAYLIVSKFQEYLQGELAQAK
M7PNV0    QQSAITGMESTKDML-ATKGRASFLGER-ARGHIDAGARTSQLMLCAIADEVIKYNN---
M5DZ01    KIKAEEGMKATIDMQ-ATKGRASYLGKR-SIGHQDPGATSSYLMIKTVVEELY-------
N4WJ21    TNAANEGVEKSKEYV-AKFGRAKTLGER-AIGYQDAGATSVWIIFDSMTNWVENHVK---
C9ABG8    E----AAKEHTKELV-AKKGRASYVGER-AIGHVDPGSASSAILFDSLLTVIEKN-----
M9WI92    VDLAYKQALYTKTII-AKKGRASYLGQR-SIDHIDPGCWSAFLILETIKENI--------
N6XD13    AAAAAEGAAATEPMK-ATKGRASYLGER-SIGHLDPGAMSSSIILAKALEAAKKA-----
N9VEI0    MEGAANGAKRTKEME-AVKGRASYRSDK-GVGHLDPGAVTLAMQLECMGNMILNTLK---
R4KG11    FDAARHGVENTKQLK-PVFGRAAYYRDK-PLGTPDSGAVAIMFVFKGIKEGLNK------
R4LHW9    VTAAEQGRDATIPMV-ARKGRASYLGER-SAGHADPGATSAAMLLTAAATALSGQD----
R2SJE3    D----EIVEKTKEIK-ATKGRASYVGER-SIGHIDPGAMSSGYLFKSMMEAGVFK-----
R2Q950    SEVIEAAKEHTKDLV-AKKGRASYLGER-AIGHIDPGAASSGILFKTLLDTIKD------
R3TPV0    IEVMKKGAESTIPLV-ANKGRAMRLGER-AIGHKDPGSESSWMLMDIFNKNLKVRSEI--
R4UJY9    ALAAQEGAEATITML-KTKGRASYLKER-SVNHMDPGAKSVSLIFEAVSEALQ-------
R4T9Z9    ADAADKGAESTVDLV-PRKGRASYLGER-AKGHMDPGARSTALLLRAFAEAAQ-------
S1P0N7    VEKITAAKEATKDLV-AKKGRASYLGER-AVGHIDPGAASSALLFTTLLTYI--------
R9KQ22    CEAAKEGVEYTKTIR-ATKGRASYLGDR-SIGHQDPGATSALITLEAVRAFYLEQVK---
S0FK13    AVSAVQGGEKTRSYV-ASKGRASYLGER-SLNYPDAGAVAIGVIFTGISKKIEDILK---
S0J6Y9    CDAAKEGVEFTKTIR-ATKGRASYLGDR-SIGHQDPGATSSLITLEAIRAFYLENIK---
R9IRK6    CKAAKEGVEYTKTIR-ATKGRASYLGDR-SIGHQDPGATSSLITLEAIQTFYLENIK---
S3B602    RAAAERGAEETTPLQ-ARKGRASYLGER-SVGHQDPGATSAALLVAALAAAHGDNAGEDA
S2XG65    Q----ALADATRDIE-ATKGRASYFKKE-SLGYVDPGAQSSVYILNALIGDEKK------
S2YZ61    GTAAEQGALATTPLQ-ARKGRASYLGER-SIGHQDPGATSSSLLVAALAEADGE------
S2W6B3    QEAAEAGRDATEPMV-AHKGRASYLGER-SAGTIDPGSASATMLVSALASA---------
S3X4B0    ARRADSSRLQTADMI-ASKGRAAFLGTR-SLGVVDPGATSASWLMAAGAEVMPSS-----
S4AMF0    RAAAHEGALATTPLQ-ARKGRASYLGER-SIGHQDPGATSSALLFEALAETAEEAAHE--
R7RQ76    VNSAKLGMENTKNIV-ATKGRASYLGER-SIGHLDPGAVSCYLILKTIFEEVKKIKG---
```

S4MG33	RTAAEEGAVATTPLQ-ARKGRASYLGER-SIGHQDPGATSAALLVAGLAEAAGE------
S9SCC0	LRTATEGAEATRAMT-ATLGRAERLGAR-AVGHPDPGAVSALMLIEEILARIVPPPAD--
S9S516	LRAADETLEDFRDRP-NRIGRARMFADR-TVGMDDPGMLAFHRLLRAVTGAEVPHA----
S5VBA9	RAAAEEGAVATTPLQ-ARKGRASYLGER-SIGHQDPGATSSALLIAALQEAASGA-----
T0TK32	KENLEKVVKASAGLL-ATKGRASYLGER-SIGHIDPGTQSSAYLFETLLEVVA-------
S0LAM8	EEKITAYVHHTKELV-AKKGRASYLGER-AIGHIDPGAQSSGYLFETLIK----------
T5KTF2	ADAAAAGRDATLPLV-ARKGRASYLGER-SAGHLDPGAASTAILFDTLAAAVADTA----
U1GE01	AQAAAQGRDATVGMI-ARRGRASYLGER-SKGHQDPGATSLALFIESAARCLPVQV----
U1MTZ3	AAAAAEGAVATEPLV-AHKGRASYLGER-AVGHRDPGAESTALILAAAAETT--------
U1N326	--KLDQLVGATEDMV-ATKGRASYFGEN-SRGTVDPGALSSSIVLKAIMKEVE-------
U2T4X1	--
U2TE92	FEAAKEGVEKTKTMQ-SVHGRAAYYREK-SVGRQDPGATAIMYIVRGIAEGVR-------
U2TIQ4	VEGAKAGVEATKDMI-PKYGKAAVFAAK-AKGIADQGAVAGCYLDTAILNYFQAKSA---
U2UUY7	KKAAEEGAENTKKME-AVRGRASYQADR-GLGHLDPGAVTMSYQLVTLMDFAAEKSREK-
U2N6F3	KEAAYNGVEYTKTII-ATKGRASYLGER-SIGHQDAGATSSAIILETIYEAIQ-------
U4VFA1	VLAAEKGRDSTIDLV-AKIGRASRLGER-SLGVLDAGATSCAIILKELSDGARVRFQ---
U5F6K8	AKGAEAGKESTKDLV-AKIGRAARLGER-SRGHLDAGATSCWIILTTMANAMLAQLQSQE
U4K1X7	EAAAKAGAESTKDLV-SKIGRSSRLGER-SVGVLDAGAVSCSLILSQLAQSTISRLQS--
U7VCP6	QITAKDGMESTENML-ATKGRASYLGAR-SIGHIDPGAMSSYLIIDTICKNLK-------
U7UHV1	VAAAEAGVAYTKTIA-AAKGRASYLGER-SIGHQDPGATSSLFMLQVIAGHIA-------
U7UNX5	KNAAINGAESTKDMP-AIRGRASYQKDK-GIGHLDPGAVTMAYQIELLCDYILNNKIGK-
U5MP41	IDAAKKGMENTKNII-ATKGRASYVGER-SLGHQDPGATSMYIILCTIAEELKF------
V2YGV3	AKAAAEGAEASKQFV-SKFGRAKSYGEK-TIGTPDAGAMSMSYFFQGLAQS---------
U5T5E1	KAAAMAGRDSTQDMI-ARIGRSSRLGER-SRGVLDAGATSCCMILCILVDNVSRRLA---
A0A2I5TCR4	RLSCQQTLNTFRPRP-NLLGRARMFPEK-SQGLDDPGMLAIKVLIDAL-----------
U5VX81	VKAAEEGTAATIPMV-ARKGRASYLGER-SAGHQDPGATSMTMLFTAAATTLSGEG----
V7IAM1	KRGAVEGMEATRGME-AVKGRATYQTNK-GVGCLDPGAVTMCYQLELLADCMKP------
A0A084A2U3	AVRAEQAAQATITMQ-ARKGRASYLGER-SIGHQDPGATSAMLMMQTLAEVVGAAG----
V5DRA3	KQTAIAGADATQNML-ATKGRASFLGER-SRGYIDAGAKSSQLMICAIVDVLTAT-----
V6KYB2	GRAAADGAS---
V6JJD1	RAAAEEGAVATIPLQ-ARKGRASYLGAR-SIGHQDPGATSSALLIAALEEAAEG------

```
V6Q9X7     AEAIASFAEETKEIK-ATKGRASYLGER-SIGHLDPGAVSSQYLFETMLEAEVI------
V2Y558     KKAAKDGADSTSNME-AVKGRATYQADK-GIGHLDPGAVTMSYQISELMNIAKERII---
G9WKH9     AEAAKKGMEYTKTIK-ARFGRAKYFGEK-AIGLQDAGATSVYIIFQAMADFVKGEEQ---
V7EEK5     ADAAAQGAAATAAMR-ARRGKSSYVGDR-SIGTVDPGAASVALLFAAMAQIGAPAP----
```
