# Supplementary material for: Compositionally Biased Regions Within Structured Protein Domains
Source: Biomolecules. 2026 Aug 22;16(9):1222. doi: 10.3390/biom16091222 (PMC13604049; doi:10.3390/biom16091222)
Supplement: Supplementary file 1 [file biomolecules-16-01222-s001.zip › Suppl-Table-S1-sequence-conservation.pdf]

Suppl. Table S1: Sequence conservation

**Length set → *** <br>**Mean sequence conservation**	**LE6** **(total 817)**	**7TO16** **(total 660)**	**GT16** **(total 709)**
LARGE Kingdom alignment	398 (49%)	332 (50%)	344 (49%)
> 0.0	277 (34%)	195 (25%)	184 (26%)
≤ 0.0	121 (15%)	137 (21%)	160 (23%)
< -0.5	46 (6%)	38 (5%)	28 (4%)

* The length sets are defined in the text. The mean conservation scores are derived from AL2CO output as described in *Methods*.
