# Supplementary material for: Compositionally Biased Regions Within Structured Protein Domains
Source: Biomolecules. 2026 Aug 22;16(9):1222. doi: 10.3390/biom16091222 (PMC13604049; doi:10.3390/biom16091222)
Supplement: Supplementary file 1 [file biomolecules-16-01222-s001.zip › Suppl-Table-S2-clade-specific-changes.pdf]

Suppl. Table S2: Clade-specific changes in sCBR size*

Data Set	Proportion with any clade-specific size change**	Proportion with any clade-specific expansion**	Proportion with any clade-specific contraction**
**All sCBRs**	861/2185 (39%)	795/2185 (36%)	182/2185 (8%)
**LB sCBRs**	172/379 (45%)	154/379 (41%)	43/379 (11%)
**LB sCBRs ≤16 residues**	90/209 (43%)	82/209 (39%)	23/209 (11%)
**Nucleotide/phosphate-binding LB sCBRs ≤16 residues**	38/90 (42%)	33/90 (37%)	13/90 (14%)
**Nucleotide/phosphate-binding {G}-rich LB sCBRs ≤16 residues**	14/26 (54%)	11/26 (42%)	5/26 (19%)
**PB sCBRs**	190/410 (46%)	179/410 (44%)	41/410 (10%)
**PB sCBRs ≤16 residues**	103/227 (45%)	99/227 (44%)	20/227 (9%)

* These data include calculations across KERNEL multiple sequence alignments, if there isn't a LARGE alignment for a sCBR.

** Calculation is described in *Methods*.
