# Supplementary material for: Compositionally Biased Regions Within Structured Protein Domains
Source: Biomolecules. 2026 Aug 22;16(9):1222. doi: 10.3390/biom16091222 (PMC13604049; doi:10.3390/biom16091222)
Supplement: Supplementary file 1 [file biomolecules-16-01222-s001.zip › Suppl-Table-S3-partner-binding.pdf]

Suppl Table S3: Ligand / protein binding

* B = significant after Bonferroni correction. Enrichments are highlighted in **green,** depletions in **red**.

** PO4b = phosphate-binding.

† SC= sequence-conserved. Conservation is a positive mean AL2CO score over either the LARGE or KERNEL MSAs.

(A) Ligand-binding frequencies (LE16)

sCBR type	Count in sample (total=209)	Count in background (total=1477)	P*
{G}_mC	25	75	8.6e-06 (B)
{G}_m1	13	18	3.6e-05 (B)
{A}_m1	5	137	2.1e-05 (B)

Primary bias	Count in sample (total=209)	Count in background (total=1477)	P*
G	48	130	4.7e-12 (B)
A	10	164	0.0004 (B)

(B) Ligand-binding Distance Calculation (LE16)

Group OR Primary bias residue **, †	Mean	Std. Deviation	Count (# of sCBRs in contact ≤5 Å)	Z

LE16 SC	37.7	3.52	147	31.02
LE16 not SC	8.29	1.56	17	5.55
LE16 PO4b	23.12	2.65	83	22.57
LE16 PO4b SC	18.56	2.35	75	23.96
LE16 PO4b not SC	4.43	1.09	8	3.29
LE16 not-PO4b	22.93	2.79	81	20.83
LE16 not-PO4b SC	19.21	2.64	72	20.0
LE16 not-PO4b not SC	3.84	1.04	9	4.99
LE16 G	5.39	1.13	46	35.79
LE16 G SC	4.42	1.12	45	36.19
LE16 G not SC	0.95	0.21	1	0.22
LE16 A	7.14	1.40	7	-0.10
LE16 A SC	5.97	1.26	7	0.81
LE16 A not SC	1.25	0.62	0	-2.02
LE16 P	2.94	0.89	5	2.33
LE16 P SC	2.33	0.72	5	3.71
LE16 P not SC	0.61	0.49	0	-1.25
LE16 V	3.34	1.02	10	6.55
LE16 V SC	2.55	0.92	7	4.82
LE16 V not SC	0.75	0.43	3	5.19
LE16 E SC	1.52	1.03	2	0.47
LE16 E not SC	0.12	0.33	0	-0.37
LE16 E	1.57	1.05	2	0.41
LE16 S SC	2.07	1.04	13	10.49
LE16 S not SC	0.78	0.44	0	-1.76
LE16 S	2.88	1.11	13	9.15
LE16 Y SC	1.35	0.79	2	0.82
LE16 Y not SC	--	--	--	--
LE16 Y	1.33	0.79	2	0.84

(C) Protein Binding Frequencies (LE16)

Primary bias	Count in sample (total=227)	Count in background (total=1477)	P*
V	2	68	0.0007 (B)

(D) Protein Binding Frequencies (GT16)

Primary bias	Count in sample (total=183)	Count in background (total=708)	P*
A	54	144	0.00019 (B)

(E) Protein-binding Distance Calculation (LE16)

Group OR Primary bias residue **, †	Mean	Std. Deviation	Count (# of sCBRs in contact ≤5 Å)	Z
LE16 V	2.65	0.64	2	-1.01
LE16 V SC	1.80	0.60	2	0.33
LE16 V not SC	0.84	0.37	0	-2.28
LE16 H	9.7	1.26	25	12.18
LE16 H SC	9.7	1.26	25	12.12
LE16 H not SC	--	--	--	--
LE16 A	11.22	1.15	27	13.7
LE16 A SC	8.26	0.89	19	12.0
LE16 A not SC	2.90	0.74	8	6.9
LE16 M not SC	0.98	0.11	3	17.7

LE16 M	6.40	0.74	20	18.3
LE16 M SC	5.43	0.70	17	16.0
LE16 P	3.09	0.95	16	13.6
LE16 P SC	3.05	0.96	13	10.3
LE16 P not SC	--	--	--	--

(F) Protein-binding Distance Calculation (GT16)

Group OR Primary bias residue **, †	Mean	Std. Deviation	Count (# of sCBRs in contact ≤5 Å)	Z
GT16 A	5.86	0.68	54	70.6
GT16 A. SC	2.82	0.38	35	84.1
GT16 A not SC	3.08	0.55	19	29.0
GT16 I	1.91	0.29	8	21.0
GT16 I. SC	1.89	0.31	5	10.1
GT16 I not SC	--	--	--	--
GT16 M	1.00	0.05	5	73.0
GT16 M. SC	1.00	0.04	5	89.3
GT16 M not SC	--	--	--	--
GT16 P	1.00	0.09	9	89.6
GT16 P. SC	--	--	--	--
GT16 P not SC	1.00	0.08	6	59.9
