# Supplementary material for: Compositionally Biased Regions Within Structured Protein Domains
Source: Biomolecules. 2026 Aug 22;16(9):1222. doi: 10.3390/biom16091222 (PMC13604049; doi:10.3390/biom16091222)
Supplement: Supplementary file 1 [file biomolecules-16-01222-s001.zip › Suppl-Table-S4-pattern-words.pdf]

Suppl. Table S4: Trawling for pattern words in sCBRs associated with ligand binding *

sCBR word and secondary structure string	Count	Extra added in from trawling longer sCBRs	Notes
GGGXG, CCCCC	9	4	All but one are nucleotide-binding
GGGXG, CCCC1	4	2	All binding SAH or SAM
VVVXV, SSSSS	3	1	All binding nucleotides
VVVV, SSSS	3	1	All binding nucleotides

* This analysis was performed for sCBRs derived from the larger *astralscop40* data set, not *astralscop40R*.
